# Systematic Analysis of the Associations between Adverse Drug Reactions and Pathways

**DOI:** 10.1155/2015/670949

**Published:** 2015-10-01

**Authors:** Xiaowen Chen, Yanqiu Wang, Pingping Wang, Baofeng Lian, Chunquan Li, Jing Wang, Xia Li, Wei Jiang

**Affiliations:** College of Bioinformatics Science and Technology, Harbin Medical University, Harbin, Heilongjiang 150081, China

## Abstract

Adverse drug reactions (ADRs) are responsible for drug candidate failure during clinical trials. It is crucial to investigate biological pathways contributing to ADRs. Here, we applied a large-scale analysis to identify overrepresented ADR-pathway combinations through merging clinical phenotypic data, biological pathway data, and drug-target relations. Evaluation was performed by scientific literature review and defining a pathway-based ADR-ADR similarity measure. The results showed that our method is efficient for finding the associations between ADRs and pathways. To more systematically understand the mechanisms of ADRs, we constructed an ADR-pathway network and an ADR-ADR network. Through network analysis on biology and pharmacology, it was found that frequent ADRs were associated with more pathways than infrequent and rare ADRs. Moreover, environmental information processing pathways contributed most to the observed ADRs. Integrating the system organ class of ADRs, we found that most classes tended to interact with other classes instead of themselves. ADR classes were distributed promiscuously in all the ADR cliques. These results reflected that drug perturbation to a certain pathway can cause changes in multiple organs, rather than in one specific organ. Our work not only provides a global view of the associations between ADRs and pathways, but also is helpful to understand the mechanisms of ADRs.

## 1. Introduction

Adverse drug reactions (ADRs) are undesired phenotypic effects that occur in human organisms after medicine administration. In recent years, ADRs have gained broad public attention. ADRs not only affect quality of life for patients, but also have become a major cause of death. Furthermore, ADRs concern the economical profit of the pharmaceutical industries. ADRs are responsible for drug candidates' failure to gain FDA approvals during clinical trials. Many marketed drugs have been withdrawn due to severe ADRs. For example, Cerivastatin was withdrawn from the world market, because this drug caused 52 deaths that were attributed to rhabdomyolysis and kidney failure [1]. Therefore, the recognition of ADRs during the early phases of drug discovery is valuable for drug development and safety [2].

One of the direct reasons for ADRs is the interaction with the primary targets or off-targets [3]. While binding of drugs to their primary targets or off-targets affected pathways, perturbed pathways can cause phenotypic effects in human biological system. If the pathway that is affected by a drug influencing the phenotype is known, then we can more effectively study the drug-related phenotype. Therefore, it is essential to detect the links between ADRs and pathways by a systemic approach. Scheiber et al. predicted targets for compounds causing ADRs using a multiple-category Bayesian model. Then they used these targets that were linked to ADRs to connect ADRs to pathways [4]. Xie et al. identified the protein-ligand binding profiles of cholesteryl ester transfer protein (CETP) inhibitors. Then, the predicted targets were merged into biological pathways via a literature review, and the results clarified the molecular mechanisms of ADRs of CETP inhibitors [5]. Furthermore, Wallach et al. predicted protein-drug interactions using* in silico* docking, which was based on information in the publicly available Protein Data Bank, and identified ADR-related pathways using logistic regression [6]. These studies are limited by their dependency on the availability of a 3D structure of the drug or target. Therefore, the methods are infeasible for many drugs and drug targets without known 3D models. Additionally, these studies usually focused on one aspect of identifying the relations among drugs, pathways, and ADRs and did not consider the global map of ADRs and pathways. It is known that network biology and pharmacology provides a way of developing drugs and understanding the mechanisms of side effects. Many studies have shown the success of network analysis in understanding biology and pharmacology [7–10]. Therefore, a large-scale ADR-pathway network should be constructed, and network analysis should be used to understand the mechanisms of ADRs.

In this study, we identified overrepresented ADR-pathway associations through combining pathway information, drug-target and drug-ADR relations. We evaluated those associations through scientific literature review and defining a pathway-based ADR-ADR similarity measure. Then, a bipartite graph of ADR-pathway interactions (ADR-pathway network) was constructed. We used network analysis on biology and pharmacology to (i) analyze topological property differences between frequent, infrequent, and rare ADRs, (ii) explore ADR-ADR associations (ADR-ADR network) based on the connections in the ADR-pathway network, and (iii) analyze ADR system organ class relations and mine the cliques with biological meaning in the ADR-ADR network. Our results provided great insights into understanding the mechanisms of ADRs.

## 2. Materials and Methods

### 2.1. Drug Targets

Human protein drug targets were collected from DrugBank [11], PDSP K_i_ database [12], Matador [13], and Therapeutic Targets Database [14]. We only considered drug-target annotations with binding affinity that were lower than 10 *μ*M [15]. Finally, 3,142 drugs, 2,920 targets (Entrez Gene ID), and 17,873 interacting pairs were obtained.

### 2.2. Adverse Drug Reactions

1,450 ADRs related to 888 drugs were downloaded from the SIDER database release 1 [16]. We also obtained information concerning the frequency of drug-related ADRs, which was defined as the percentage of patients who reported the ADR after taking the drug. The frequency can be a general range (rare, infrequent, and frequent) or an exact value (e.g., 3.1%). The SIDER database provided the exact lower/upper bounds for the general frequency range. Rare is limited to the range [0, 0.001]. Infrequent is limited to the range [0.001, 0.01]. Frequent is limited to the range [0.01, 1]. We grouped one ADR into three categories (rare, infrequent, and frequent) according to the median frequency of its drugs and the lower (upper) bound of the general range.

### 2.3. Pathways

KEGG database was used to obtain the biological pathway information for this study [17]. “Global pathway” was excluded from the pathway set. According to the pathway classification of KEGG database, all the pathways were partitioned into six categories: cellular processes (CP), environmental information processing (EIP), genetic information processing (GIP), human diseases (HD), metabolism (MB), and organismal systems (OS).

### 2.4. The ADR-Pathway Network

**Figure 1:**
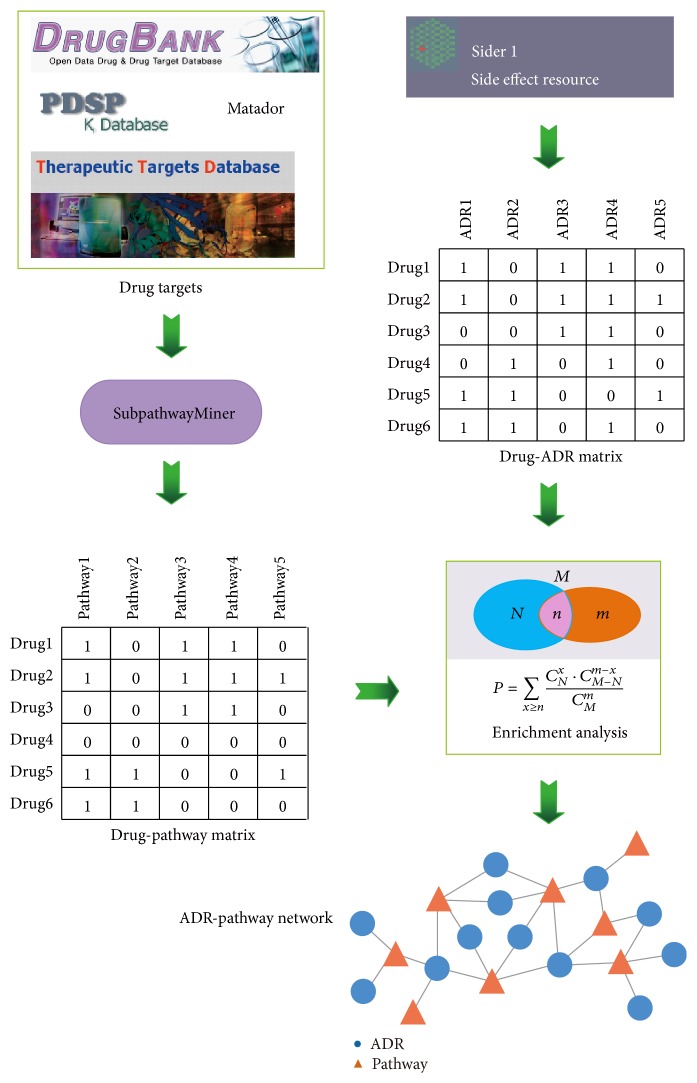
A framework for ADR-pathway network construction. Current knowledge was obtained through two data types: drug targets from DrugBank, PDSP K_i_, Matador, and Therapeutic Targets Database, and drug-induced ADRs from the SIDER database. The affected pathways for each drug were found by inputting targets of the drug into SubpathwayMiner. Then we applied enrichment analysis method to identify associations between ADRs and pathways based on drug-ADR relations and drug-pathway relations. Finally, we combined these ADR-pathway associations to construct an ADR-pathway network.

We connected ADRs to pathways through the pathway-drug and drug-ADR relations. The hypothesis is that pathways that are affected frequently by drugs causing the same ADR have higher probabilities of correlation with an ADR than pathways that are affected less frequently. Firstly, we identified drug-perturbed pathways using the SubpathwayMiner software package [18] (see Figure 1). After inputting targets of a drug, hypergeometric test was performed to obtain the statistically significant enriched pathways with *P* ⩽ 0.05. By applying this method for all the drugs, drug-pathway relations were obtained. These relations were represented by a drug-pathway matrix, in which rows represent drugs, columns represent pathways, and the *ij*th element is 1, if drug *i* perturbs pathway *j*, and is 0 otherwise. Similarly, drug-ADR relations from the SIDER database were represented by a drug-ADR matrix, whose rows represent drugs and columns represent ADRs, in which the *ij*th element is 1 if drug *i* induces ADR *j* and is 0 otherwise. Then, to identify the associations between ADRs and pathways, we used drug information to combine the drug-pathway matrix with the drug-ADR matrix. 572 drugs were involved in the above two matrices. Therefore, we used 572 drugs with 1,267 ADRs and 194 pathways to find the associations between ADRs and pathways based on enrichment analysis. An ADR was associated with a pathway, when drugs causing the ADR were significantly enriched in the set of drugs that affected the pathway. The significance of the enrichment was evaluated by the *P* values of the hypergeometric test. Finally, we constructed an ADR-pathway network consisting of ADRs and ADR-associated pathways.

### 2.5. Closeness between ADRs' Drug Sets

For any two ADRs *i* and *j*, the drug sets that induced them were denoted by *D*_*i*_ and *D*_*j*_, respectively. We calculated association score (AS) to evaluate the extent of closeness between the two drug sets *D*_*i*_ and *D*_*j*_. The AS was defined as follows:

(1) $$\begin{array}{c} AS\left(\;D_{i},D_{j}\;\right)=\frac{1}{N_{i}\times N_{j}}\overset{N_{i}}{\underset{a=1}{\sum }}\overset{N_{j}}{\underset{s=1}{\sum }}FER\left(\;a,s\;\right), \end{array}$$

where *N*_*i*_ and *N*_*j*_ denote the number of drugs in the drug sets *D*_*i*_ and *D*_*j*_, respectively. The FER (fold enrichment ratio) is used to quantify the extent of the closeness between any drug *a* in the drug set *D*_*i*_ and any drug *s* in the drug set *D*_*j*_. It is defined as FER(*a*, *s*) = *O*/*E*, *O* equals *n*_*as*_ which is the observed value, and *E* equals (*n*_*a*_ × *n*_*s*_)/*N* which is the expected value [19], where *n*_*a*_ is the number of indications related to drug *a*, *n*_*s*_ is the number of indications related to drug *s*, *n*_*as*_ is the number of indications shared by drug *a* and drug *s*, and *N* is the number of the unions of indications which related to the drug set *D*_*i*_ or the drug set *D*_*j*_. The higher the AS the more the same indications shared by the drug set *D*_*i*_ of ADR *i* and the drug set *D*_*j*_ of ADR *j*. The AS could quantify the extent of the closeness between the two drug sets.

### 2.6. Clique Identification

The CFinder software was used to detect *k*-cliques in the ADR-ADR network based on the Clique Percolation Method (CPM) [20]. A *k*-clique is a maximal complete subgraph. In this context, *k* equals 6, which is defined as the number of nodes in the subgraph.

## 3. Results and Discussion

In this paper, we reported a large-scale analysis to systematically extract and characterize ADR-pathway associations. We combined clinical phenotypic data, biological pathway data, and drug-target relations to identify overrepresented ADR-pathway associations. Effectiveness examination was performed in the following two ways: firstly, we calculated how many ADR-pathway associations were identified by our method and were concurrently found in PubMed records using a text-mining tool; second, we defined a pathway-based ADR-ADR similarity measure and validated whether this measure can be used to quantify relation between two ADRs. Then, an ADR-pathway network and an ADR-ADR network were constructed, and network analysis on biology and pharmacology was applied to effectively and systematically understand the mechanisms of ADRs.

### 3.1. Construction of the ADR-Pathway Network

**Figure 2:**
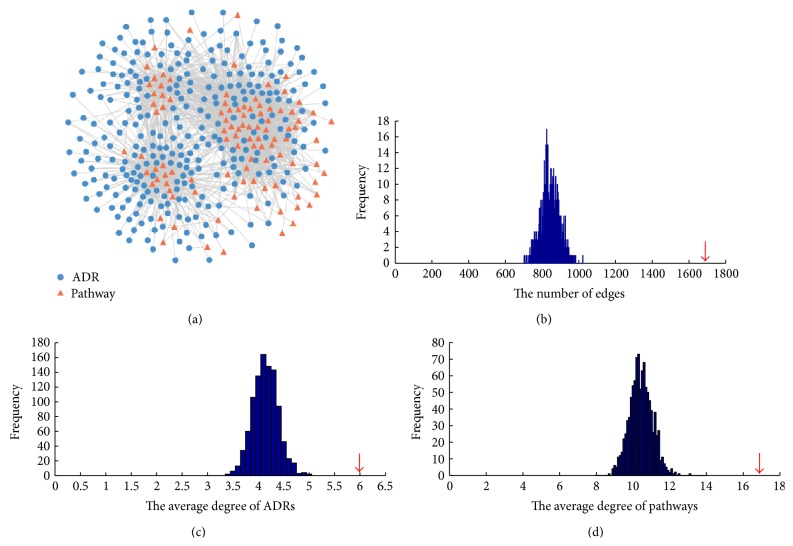
The AP network. (a) In the AP network, the blue circles correspond to ADRs, and the red triangles correspond to biological pathways. An edge is placed between an ADR node and a pathway node when drugs that induced the ADR are significantly enriched in the set of drugs that perturbed the pathway. (b) The distribution of ADR-pathway interactions in the random AP networks. The *x*-axis denotes the edge number, and the *y*-axis is the frequency of the edge numbers in random AP networks (the edge number of the real AP network is labeled in red arrow). ((c), (d)) The distributions of the average degrees of ADR and pathway nodes in the random AP networks. The red arrows denote the average degrees of ADR and pathway nodes in real AP network, respectively.

To systematically identify pathways that were associated with ADRs, we integrated ADR data for marketed drugs from the SIDER database with enriched biological pathways by drug targets (see Section 2). Initially, human targets and off-targets for 3,142 drugs were utilized to identify the statistically significant drug-perturbed pathways. After removing drugs without significantly enriched pathways, we used the 572 drugs with 1,267 ADRs and 194 pathways to find associations between ADRs and pathways (see Supplementary Figure S1 for histograms in Supplementary Material available online at http://dx.doi.org/10.1155/2015/670949). An ADR was associated with a pathway, when the drugs causing the ADR significantly overlapped with the drugs that affected the pathway (*P* ≤ 0.01) and when the number of common drugs that were shared by the ADR and the pathway was more than 10. Finally, we obtained 1,694 ADR-pathway associations covering 284 ADRs and 101 pathways (see Supplementary Dataset S1). Based on these associations, an ADR-pathway (AP) network was constructed (see Figure 2(a)).

### 3.2. Properties of the ADR-Pathway Network

Randomization tests were performed to evaluate whether the AP network was generated randomly. We permuted the relations between drugs and ADRs 1,000 times randomly, while keeping the degree of each node unchanged. The edges in the AP network were significantly denser than that in 1000 random networks (*P* < 0.001, Figure 2(b)), and the average degrees of ADR and pathway nodes in the AP network were significantly higher than that of 1000 random networks (*P* < 0.001, Figures 2(c) and 2(d)). These results reflected that the connections between ADRs and pathways had biological significance, instead of random connections.

**Figure 3:**
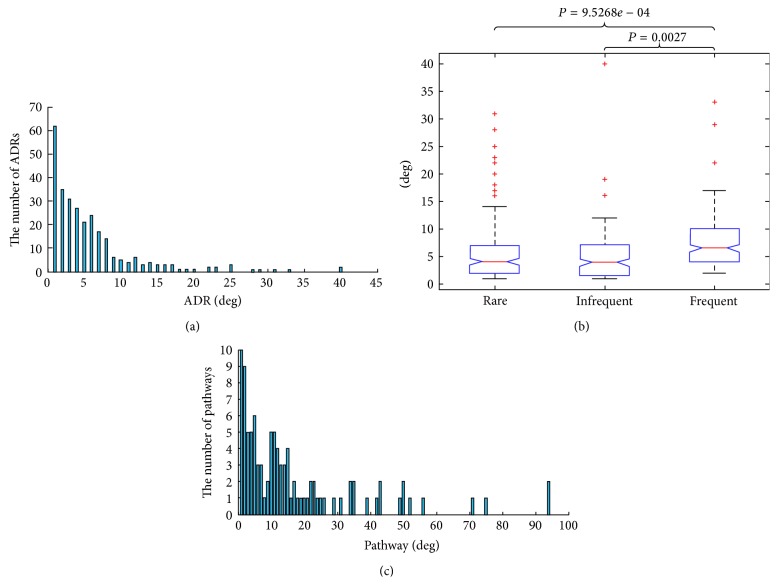
The properties of the AP network. (a) Degree distribution of the ADRs. (b) The box plot for ADR degrees in 3 frequency categories. (c) Degree distribution of the pathways.

We calculated the degree of every ADR node, which was the number of pathways that were linked to the ADR node under investigation (see Figure 3(a)). Anemia and dyspnea were the highest degree ADR nodes, which were associated with the most pathways (degree = 40). Many drugs eliciting these ADRs were antineoplastic agents, which can affect many tissues including the heart and lung [21, 22]. 78% of all the ADR nodes were linked to more than one pathway, implying that many ADRs were the compositive outcome of the alteration of multiple biological pathways. Then, we focused on the difference in the degrees of three ADR groups (rare, infrequent, and frequent ADRs), which was illustrated in Figure 3(b). The degrees of the rare ADRs, infrequent ADRs, and frequent ADRs were compared using the Kruskal-Wallis test. The degrees of these ADR classes were significantly different (*P* = 0.0033). Furthermore, we used the Wilcoxon rank-sum test to compare the degrees of frequent ADRs with the degrees of infrequent ADRs and rare ADRs, respectively. As a result, the degrees of frequent ADRs were significantly higher than that of infrequent ADRs (*P* = 0.0027) and that of rare ADRs (*P* = 9.5268 × 10^−4^). This statistical result indicated that frequent ADRs were linked to more pathways than infrequent ADRs and rare ADRs. Similarly, we calculated the degree of every pathway node in the AP network, which was the number of ADRs that were linked to the pathway under investigation (see Figure 3(c)). The linoleic acid metabolism and gap junction pathways were associated with most ADRs (degree = 94). 90% of all the pathway nodes were associated with more than one ADR, implying the disturbance of most pathways induced concurrency of multiple ADRs. Additionally, the degree distributions of ADR and pathway nodes in the AP network both followed power-law distributions with *P* = 0.8608 and *P* = 0.7956, respectively (using igraph package in *R*). This implied that the AP network displayed scale-free characteristics.

**Figure 4:**
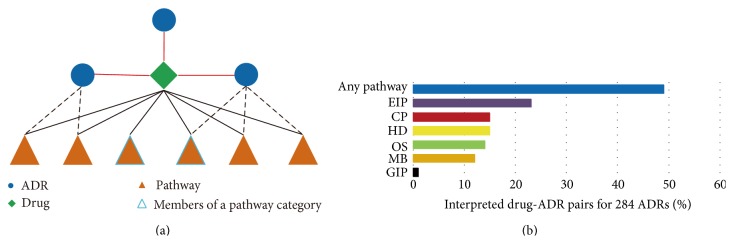
Drug-ADR pairs interpreted by pathway categories. (a) For this given drug, two of three drug-ADR pairs can be interpreted by the identified ADR-pathway associations. If we concerned only one pathway category, then only a drug-ADR pair was interpreted. (b) The contributions of any pathway and each pathway category to ADRs were quantified. Only 284 ADRs in the AP network were investigated.

The inferred ADR-pathways associations can be used to interpret an observed drug-ADR pair through searching pathways that were associated with the ADR among pathways affected by the drug. To quantify the contributions of pathway categories to ADR etiology, we classified all relevant pathways into six categories according to the KEGG database. For each pathway category, we searched the members of this pathway category among the drug-affected pathways that can interpret a drug-ADR pair (see Figure 4(a)). We found that environmental information processing (EIP) contributed most to the observed 284 ADRs in the AP network (see Figure 4(b)). This may mirror that a large number of different drugs affected EIP pathways, and the number of known targets for these drugs is high. EIP pathways respond to external environment stimuli and internal environment changes for maintaining cellular homeostasis. Therefore, ADRs perhaps are caused by the alteration of EIP pathways. Recently, Ji et al. predicted drug effects based on the change at midstage of signal transduction [23].

### 3.3. Evaluation Result

We investigated the relevant scientific literature to validate the inferred ADR-pathway associations. Firstly, each of all the ADRs in the AP network was used as a query. In order to insure that the ADR was mentioned in abstracts or titles, the “[abstract/title]” qualifier was applied in the PubMed search. Second, because pathway terms are noun phrases, it is not easy to detect pathway terms in the literature through a simple exact string match. We extracted pathway terms using a text-mining tool PathNER which used soft dictionary matching and rules to identify pathway terms in the literature [24]. Of 1,694 ADR-pathway associations identified by our method, 727 were found in the text-mining results.

To further validate the inferred ADR-pathway associations, we proposed a pathway-based ADR-ADR similarity measure method using hypergeometric enrichment. Namely, two ADRs were similar when the pathways that were associated with the two ADRs significantly overlapped based on hypergeometric test (*P* ≤ 0.01). To examine the ability of the pathway-based ADR-ADR similarity measure to quantify ADR relations, we calculated the closeness between drug sets of two ADRs (see Section 2). The background set was defined as the drug sets of two dissimilar ADRs. We found that similar ADR pairs' drug sets significantly shared more indications than the background set (*P* = 1.8261 × 10^−57^, Wilcoxon rank-sum test). This result indicated drug sets of similar ADRs were closer than that of dissimilar ADRs, implying that our method can link similar ADRs to one pathway. Therefore, our method can efficiently identify the relations between ADRs and pathways.

### 3.4. Case Study: Heart Failure-Associated Pathways

**Figure 5:**
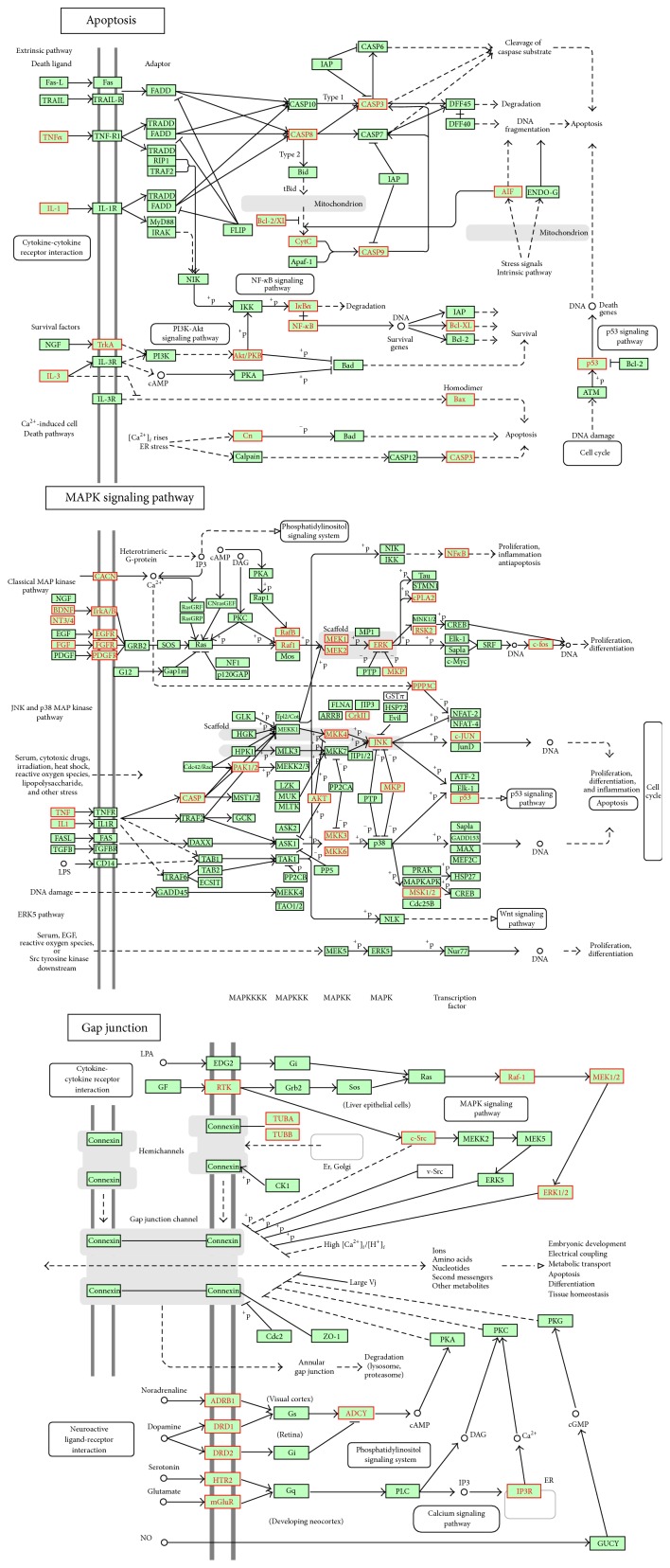
The three pathways that were associated with heart failure were shown. The red nodes represented targets of drugs that caused heart failure.

Heart failure (HF) is a condition in which the heart is unable to pump out sufficient blood to meet the need of the body. The features of HF are systolic/diastolic dysfunction and impaired electrical conduction [25]. Upon the seven identified HF-associated pathways, we can understand the molecular mechanisms of HF. It was proposed that HF was likely caused in the following three routes (see Figure 5). (1) Direct interactions with proteins like p53, TNF, IL-1, IL-3, or other proteins caused apoptosis, which was a hallmark of etiologies of HF [26]. (2) Activation of MAPK signaling pathway by undesired interactions with proteins like FGF, ERK, and JNK caused HF through regulating cardiac remodeling, cardiomyocyte apoptosis, and cardiac hypertrophy [27–29]. (3) Gating properties of gap junction can be changed by active mitogen-activating protein (MAP) kinase and c-Src, which can cause conductance decrease [25, 30]. Altered Ca2+-release channel levels via protein IP3R in gap junction may be responsible for defects in Ca2+ homeostasis, which was implicated in HF [31]. Besides, pathways including bladder cancer, colorectal cancer, toxoplasmosis, and epithelial cell signaling in helicobacter pylori infection were connected to the MAPK signaling pathway and apoptosis and were associated with HF.

### 3.5. Constructing the ADR-ADR Network

**Figure 6:**
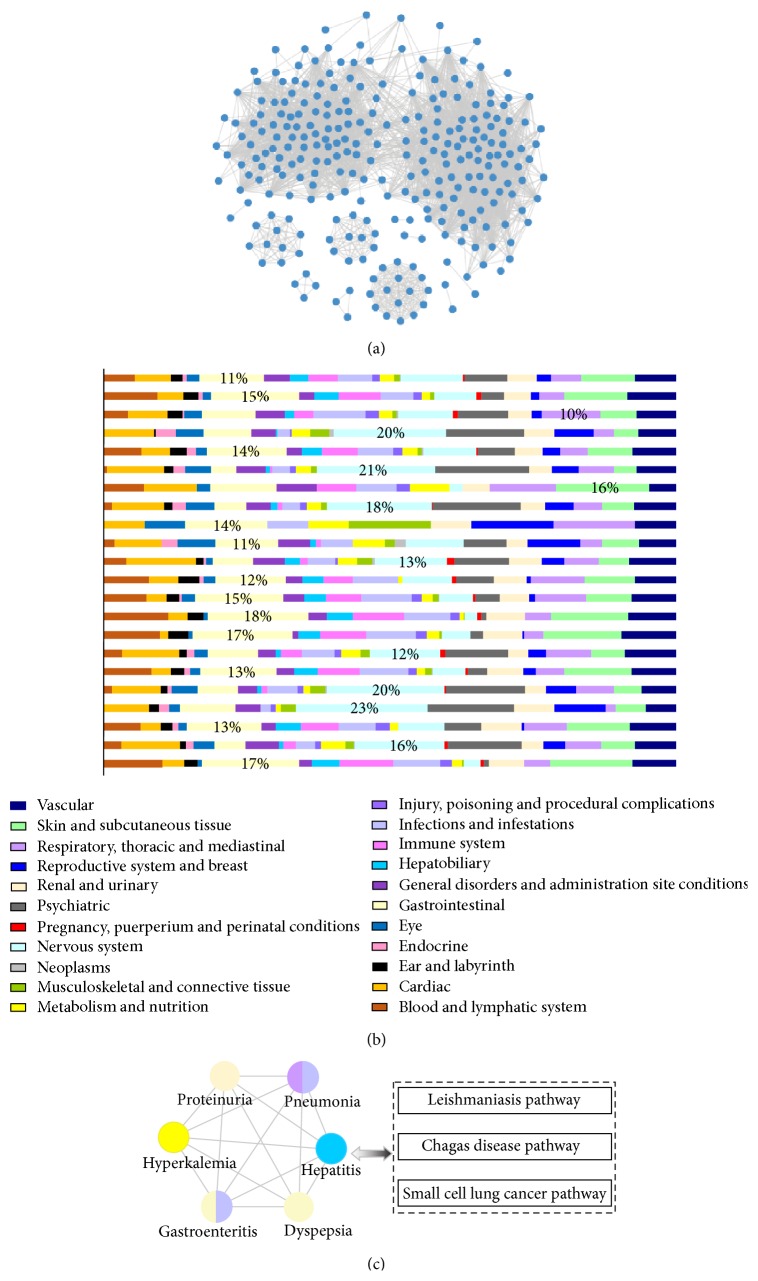
The AA network and its properties. (a) The AA network. Each node is an ADR, and two ADRs were connected by a link when the pathways that were associated with these ADRs significantly overlapped. The significance of the overlapping was also evaluated by the *P* values of the hypergeometric test (*P* ≤ 0.01). (b) The heatmap of interaction frequency between any two SOCs. The highest percentage of interaction frequency of every SOC was labeled on the corresponding color column. (c) ADR clique 20. All ADRs in this clique were associated with three identical pathways. Every ADR node was colored according to the SOCs to which the ADR belonged.

To better understand the mechanisms of ADRs, we constructed an ADR-ADR (AA) network in which two ADR nodes were connected if they were similar according to the pathway-based ADR-ADR similarity measure. The AA network contained 273 ADRs and 3,241 interactions (see Figure 6(a) and Supplementary Dataset S2). The average interaction number per ADR was 23.7. The characteristic path length, which was defined as the average number of links in the shortest path between two nodes, was 2.637. The result showed that the AA network was tightly connected. The degree distribution of ADRs followed a power-law distribution with *P* = 0.9617 (using igraph package in *R*). Like many other known biological networks, the AA network also showed scale-free characteristics.

Medical Dictionary for Regulatory Activities (MedDRA) is a standardized medical terminology that is used for adverse event reporting in the USA, European Union, and Japan [32]. MedDRA possesses a hierarchy structure that contains five levels. We obtained the top level system organ class (SOC) for ADRs. To analyze interacting ADR classes from a global view, we investigated the interaction frequency between any two SOCs in the AA network. For an ADR of one SOC (e.g., “hepatobiliary disorders”), we calculated the number of the ADR's neighborhoods which belonged to another SOC (e.g., “eye disorders”). The calculation procedure was repeated for all the ADRs in the SOC “hepatobiliary disorders” and the corresponding numbers were summed to gain the interaction frequency between “hepatobiliary disorders” and “eye disorders.” In this manner, we obtained the interaction frequency between any two SOCs for 23 SOCs involved in the AA network. Here, only one ADR “SGOT increased” in the AA network was an element of the SOC “investigations,” and the degree of the ADR equals 1. Hence, this SOC whose interaction frequency is 1 is excluded. Finally, for one SOC we calculated the fraction of interaction frequency between the SOC and each of the 22 SOCs normalized by total amount of the SOC's interaction frequency. The outcome was visualized in Figure 6(b). We found that only the three SOCs had high interaction frequency with themselves, particularly “gastrointestinal disorders,” “nervous system disorders,” and “respiratory, thoracic and mediastinal disorders.” Most SOCs (19/22) frequently interacted with other SOCs rather than themselves. Interactions between SOCs were promiscuous. It suggested that the perturbation of the pathways by drugs may affect various organs rather than a particular organ, which was consistent with the previous study [4]. Such a result also supported the effective performance of our method in identifying the associations between ADRs and pathways. Furthermore, interaction frequencies with all SOCs were different for one SOC. Except “pregnancy, puerperium and perinatal conditions” and “respiratory, thoracic and mediastinal disorders,” the remaining 20 SOCs interacted with “gastrointestinal disorders” or “nervous system disorders” more frequently than other SOCs, with 12 SOCs that interacted frequently with “gastrointestinal disorders” and 8 SOCs that interacted frequently with “nervous system disorders.” This analysis has provided useful information. When an ADR in one organ was observed, gastrointestinal organs and the nervous system were most likely affected. For example, “psychiatric disorders” frequently interacted with “nervous system disorders” which was consistent with the previous research [33]. Simultaneously, “nervous system disorders” interacted with “psychiatric disorders” more frequently than other SOCs. This result showed that ADRs from these two organs were likely to be cooccurring terms.

Finally, we used CFinder [20] to mine the cliques in the AA network (as shown in Supplementary Table S1). All ADRs in one clique were fully connected with each other. Any two connected ADRs significantly shared the same pathways according to the method of constructing the AA network. For example, ADR clique 20, which was shown in Figure 6(c), shared three pathways: the leishmaniasis pathway, Chagas disease pathway, and small cell lung cancer pathway. Subsequently, we considered the SOC distribution in ADR cliques. To this end, we calculated the number of SOCs in each clique, which spanned a range from 4 to 8. Then, for each SOC in one clique, we measured the percentage of ADRs belonging to the SOC. The ADR cliques with percentages ≥0.5 were listed in Table 1, where the largest percentage was 0.83. This result indicated that there was none of ADR clique, in which all the ADRs belonged to the same SOC. In other words, all of the ADR cliques were heterogeneous. Therefore, SOCs were distributed promiscuously in all the cliques, implying that the change in common pathways can cause unwanted phenotypic effects in different system organs. We further supported the above result that drug perturbation to a certain pathway may affect multiple organs instead of one particular organ.

### 3.6. Discussion

It is worth noting that our work can only identify pathways that are affected by a certain amount of drugs. If a pathway is affected by a very few drugs, then it is unable to associate observed ADRs with this pathway. However, this will be alleviated through completing drug-target relations. With the development of cheminformatics, more precise associations between ADRs and biological pathways will be identified. Another limitation is that examination* in vivo* of novel predicted ADR-pathway associations needs further investigation.

In this study, we proposed a new concept to identify the ADR-pathway associations using enrichment analysis based on multilevel relation. Compared with previous works, our method used existing drug-target relations. Therefore, our results do not depend on the selection and accuracy of various drug-target prediction algorithms or require structural information concerning compounds and proteins. Additionally, the network analysis method was used to systematically analyze ADR-pathway associations. The results showed that ADR-related pathways were valuable resources in elucidation of the mechanisms of ADRs and investigation of the concurrence of ADRs.

## 4. Conclusions

The inferred ADR-pathway relations can be used to elucidate the mechanisms of drug side effects. To this end, we used hypergeometric test, which is a typical method for measuring the significance of the associations between two variables, to identify ADR-pathway associations. We evaluated the performance of the method by using a text-mining tool to obtain cooccurring pathway terms with ADRs and defining a pathway-based ADR-ADR similarity measure. The results suggested that our method can be efficient for identifying associations between ADRs and pathways. Then, we constructed the ADR-pathway network based on the identified ADR-pathway associations. This network contributes to the investigation of drug-activated biological pathways that regulate phenotypic changes in cells. For example, the pathogenesis of heart failure was likely attributed to three routes: apoptosis, gap junction, and MAPK signaling pathway. Topological property analysis revealed that, on the one hand, disturbance of most pathways led to concurrency of multiple ADRs and, on the other hand, many ADRs were caused through synthetically affecting multiple biological pathways. We also found that the degrees among the rare ADRs, infrequent ADRs, and frequent ADRs were significantly different. Frequent ADRs were associated with more pathways than rare and infrequent ADRs. To uncover the reasons for this phenomenon, we calculated the number of all drugs causing one ADR and the number of total targets of these drugs. Although frequency of one ADR is defined as the percentage of patients reporting the ADR after taking drug rather than the number of drugs with the ADR, we found that frequent ADRs were linked to more drugs and drug sets eliciting frequent ADRs were linked to more targets. We further investigated the contributions of pathway categories to ADR etiology. The results showed that EIP contributed most to the observed ADRs. Subsequently, an AA network was generated according to the pathway-based ADR-ADR similarity measure. After accessing the interaction frequency between any two SOCs, we found that SOCs frequently interacted with other SOCs rather than themselves, indicating that perturbation to a certain pathway may affect multiple system organs instead of one specific organ. It was further supported by investigating the SOC distribution in all the ADR cliques. These results can provide references for side effect assessment in drug design. When an ADR in one SOC presented after drug administration, the biological characteristics of the organs that frequently interacted with the SOC should be investigated. Finally, there is a new speculation concerning ADR cliques. ADRs in one clique shared the same pathway according to the construction method of the AA network. Then, after drug treatment, the corresponding pathway was affected by this drug. The ADRs in the same clique likely cooccurred. For example, for ADR clique 20, patients with rheumatoid arthritis experienced adverse events, including dyspepsia, pneumonia, and gastroenteritis after Tacrolimus treatment [34]. Additionally, patients with hepatitis had proteinuria and hyperkalemia complications [35]. The cross talk between ADRs was established via their common pathways. The AA network and ADR cliques may be used to elucidate the concurrence of ADRs. Therefore, it would provide an entry point for investigating ADR relations through recognition of common pathways. In conclusion, this ADR and pathway study provides a global and powerful approach to discuss the molecular mechanisms of ADRs.

## Supplementary Material

Supplementary Figure S1: Statistics of the drug-ADR relations and the drug-pathway relations The number of drugs is counted for each ADR, and the number of drugs per ADR is plotted versus the number of ADRs. For example, there are 25 ADRs with 9 drugs. Similarly, the number of drugs per pathway is plotted.
Supplementary Dataset S1: The ADR-pathway pairs in the ADR-pathway network.
Supplementary Dataset S2: The ADR-ADR pairs in the ADR-ADR network.
Supplementary Table S1: All cliques in the ADR-ADR network.

## Figures and Tables

**Table 1:** The cliques with at least half of ADRs sharing the same SOC.

Clique	*r *	Common SOC
Clique_17	0.83	Skin and subcutaneous tissue
Clique_18	0.83	Skin and subcutaneous tissue
Clique_21	0.66	Skin and subcutaneous tissue
Clique_22	0.66	Skin and subcutaneous tissue
Clique_10	0.5	Blood and lymphatic system
Clique_11	0.5	Blood and lymphatic system
Clique_26	0.5	Infections and infestations
Clique_27	0.5	Respiratory, thoracic and mediastinal disorders

*r* is the percentage of the ADRs belonging to one SOC in a clique.
