# Supplementary material for: Systematic Analysis of the Associations between Adverse Drug Reactions and Pathways
Source: Biomed Res Int. 2015 Oct 1;2015:670949. doi: 10.1155/2015/670949 (PMC4606217; doi:10.1155/2015/670949)
Supplement: Supplementary file 1 — Supplementary Figure S1: Statistics of the drug-ADR relations and the drug-pathway relations The number of drugs is counted for each ADR, and the number of drugs per ADR is plotted versus the number of ADRs. For example, there are 25 ADRs with 9 drugs. Similarly, the number of drugs per pathway is plotted. Supplementary Dataset S1: The ADR-pathway pairs in the ADR-pathway network. Supplementary Dataset S2: The ADR-ADR pairs in the ADR-ADR network. Supplementary Table S1: All cliques in the ADR-ADR network. [file 670949.f1.pdf]

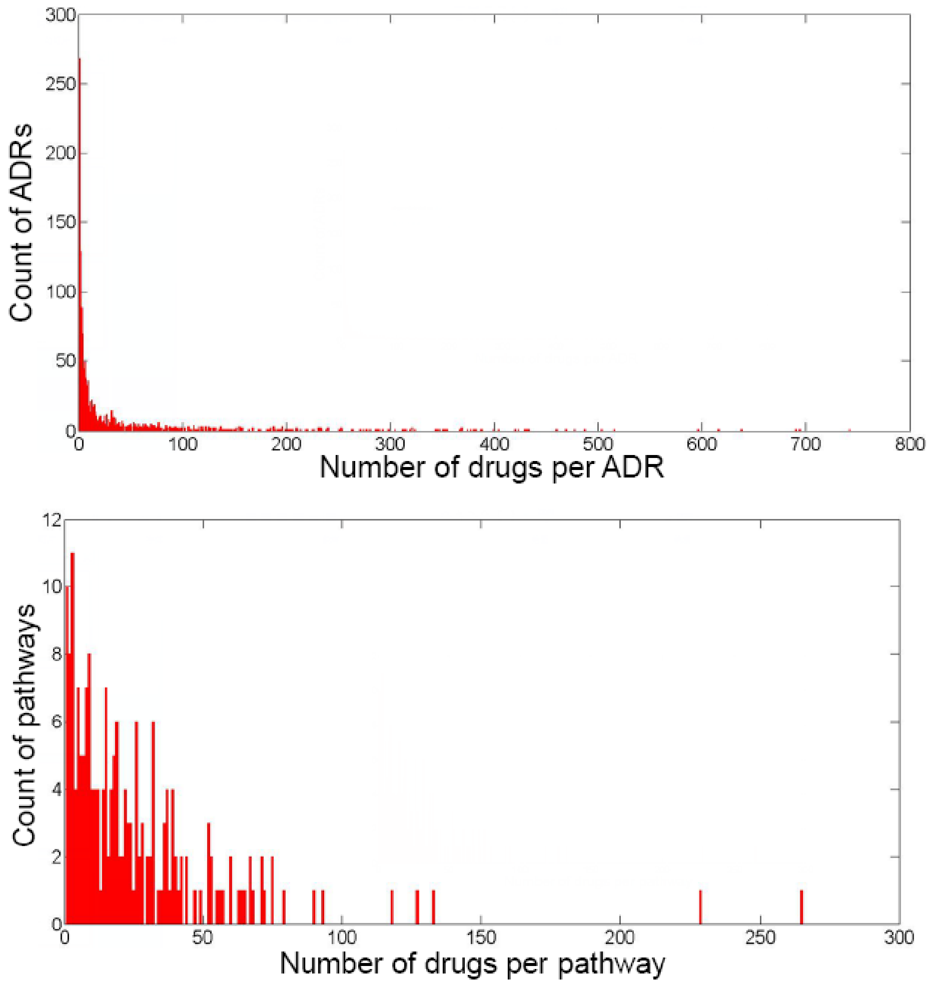

Figure S1. Statistics of the drug-ADR relations and the drug-pathway relations

Dataset S1. The ADR-pathway network

pathway code	pathway name	ADR
path:00140	Steroid hormone biosynthesis	myocardial infarction
path:00140	Steroid hormone biosynthesis	tumor
path:00140	Steroid hormone biosynthesis	thrombophlebitis
path:00140	Steroid hormone biosynthesis	breast pain
path:00140	Steroid hormone biosynthesis	laryngitis
path:00140	Steroid hormone biosynthesis	pulmonary embolism
path:00140	Steroid hormone biosynthesis	urinary frequency
path:00230	Purine metabolism	seizures
path:00230	Purine metabolism	supraventricular tachycardia
path:00232	Caffeine metabolism	tremor
path:00232	Caffeine metabolism	coma
path:00232	Caffeine metabolism	ataxia
path:00232	Caffeine metabolism	diplopia
path:00240	Pyrimidine metabolism	leukopenia
path:00240	Pyrimidine metabolism	constitutional symptoms
path:00240	Pyrimidine metabolism	hypersensitivity
path:00240	Pyrimidine metabolism	anorexia
path:00240	Pyrimidine metabolism	anemia
path:00240	Pyrimidine metabolism	pain
path:00240	Pyrimidine metabolism	neutropenia
path:00240	Pyrimidine metabolism	alopecia
path:00240	Pyrimidine metabolism	stomatitis
path:00240	Pyrimidine metabolism	myalgia
path:00240	Pyrimidine metabolism	infection
path:00240	Pyrimidine metabolism	diarrhea
path:00240	Pyrimidine metabolism	dyspnea
path:00240	Pyrimidine metabolism	thrombocytopenia
path:00380	Tryptophan metabolism	impotence
path:00380	Tryptophan metabolism	shock
path:00590	Arachidonic acid metabolism	gastrointestinal hemorrhage
path:00590	Arachidonic acid metabolism	glossitis
path:00590	Arachidonic acid metabolism	erythema multiforme
path:00590	Arachidonic acid metabolism	elevated liver enzymes
path:00590	Arachidonic acid metabolism	hematemesis
path:00590	Arachidonic acid metabolism	ulcerative stomatitis
path:00590	Arachidonic acid metabolism	eructation
path:00590	Arachidonic acid metabolism	gastritis
path:00590	Arachidonic acid metabolism	dysuria
path:00590	Arachidonic acid metabolism	hearing loss
path:00590	Arachidonic acid metabolism	heartburn
path:00590	Arachidonic acid metabolism	exfoliative dermatitis
path:00590	Arachidonic acid metabolism	ecchymosis
path:00590	Arachidonic acid metabolism	pancreatitis
path:00590	Arachidonic acid metabolism	stomatitis
path:00590	Arachidonic acid metabolism	Stevens - Johnson syndrome
path:00590	Arachidonic acid metabolism	sepsis
path:00590	Arachidonic acid metabolism	rectal hemorrhage
path:00590	Arachidonic acid metabolism	proteinuria
path:00590	Arachidonic acid metabolism	peptic ulcer
path:00590	Arachidonic acid metabolism	interstitial nephritis
path:00590	Arachidonic acid metabolism	pancytopenia
path:00590	Arachidonic acid metabolism	hematuria
path:00590	Arachidonic acid metabolism	melena
path:00590	Arachidonic acid metabolism	hepatic failure

path:00590	Arachidonic acid metabolism	jaundice
path:00590	Arachidonic acid metabolism	toxic epidermal necrolysis
path:00590	Arachidonic acid metabolism	herpes simplex
path:00590	Arachidonic acid metabolism	hepatitis
path:00590	Arachidonic acid metabolism	hemolytic anemia
path:00590	Arachidonic acid metabolism	pelvic pain
path:00590	Arachidonic acid metabolism	colitis
path:00590	Arachidonic acid metabolism	cystitis
path:00590	Arachidonic acid metabolism	agranulocytosis
path:00590	Arachidonic acid metabolism	angioedema
path:00590	Arachidonic acid metabolism	albuminuria
path:00590	Arachidonic acid metabolism	aplastic anemia
path:00590	Arachidonic acid metabolism	congestive heart failure
path:00590	Arachidonic acid metabolism	asthma
path:00591	Linoleic acid metabolism	breast pain
path:00591	Linoleic acid metabolism	menorrhagia
path:00591	Linoleic acid metabolism	migraine
path:00591	Linoleic acid metabolism	ataxia
path:00591	Linoleic acid metabolism	myocardial infarction
path:00591	Linoleic acid metabolism	liver function tests abnormal
path:00591	Linoleic acid metabolism	neck rigidity
path:00591	Linoleic acid metabolism	nervousness
path:00591	Linoleic acid metabolism	tenesmus
path:00591	Linoleic acid metabolism	neurosis
path:00591	Linoleic acid metabolism	lymphadenopathy
path:00591	Linoleic acid metabolism	otitis media
path:00591	Linoleic acid metabolism	leukopenia
path:00591	Linoleic acid metabolism	laryngitis
path:00591	Linoleic acid metabolism	carcinoma
path:00591	Linoleic acid metabolism	hemorrhoids
path:00591	Linoleic acid metabolism	tinnitus
path:00591	Linoleic acid metabolism	Dyspepsia
path:00591	Linoleic acid metabolism	jaundice
path:00591	Linoleic acid metabolism	interstitial nephritis
path:00591	Linoleic acid metabolism	colitis
path:00591	Linoleic acid metabolism	insomnia
path:00591	Linoleic acid metabolism	infection
path:00591	Linoleic acid metabolism	malaise
path:00591	Linoleic acid metabolism	syncope
path:00591	Linoleic acid metabolism	stomatitis
path:00591	Linoleic acid metabolism	acidosis
path:00591	Linoleic acid metabolism	Stevens - Johnson syndrome
path:00591	Linoleic acid metabolism	SIADH
path:00591	Linoleic acid metabolism	shock
path:00591	Linoleic acid metabolism	albuminuria
path:00591	Linoleic acid metabolism	alopecia
path:00591	Linoleic acid metabolism	rectal hemorrhage
path:00591	Linoleic acid metabolism	amblyopia
path:00591	Linoleic acid metabolism	amnesia
path:00591	Linoleic acid metabolism	pulmonary embolism
path:00591	Linoleic acid metabolism	anemia
path:00591	Linoleic acid metabolism	nightmares
path:00591	Linoleic acid metabolism	photosensitivity
path:00591	Linoleic acid metabolism	arthrosis
path:00591	Linoleic acid metabolism	phlebitis
path:00591	Linoleic acid metabolism	peripheral neuropathy

path:00591	Linoleic acid metabolism	tachycardia
path:00591	Linoleic acid metabolism	Parkinson
path:00591	Linoleic acid metabolism	paresthesia
path:00591	Linoleic acid metabolism	paralysis
path:00591	Linoleic acid metabolism	angioedema
path:00591	Linoleic acid metabolism	pancreatitis
path:00591	Linoleic acid metabolism	apnea
path:00591	Linoleic acid metabolism	chills
path:00591	Linoleic acid metabolism	nocturia
path:00591	Linoleic acid metabolism	increased salivation
path:00591	Linoleic acid metabolism	polyuria
path:00591	Linoleic acid metabolism	thrombophlebitis
path:00591	Linoleic acid metabolism	glycosuria
path:00591	Linoleic acid metabolism	constipation
path:00591	Linoleic acid metabolism	flatulence
path:00591	Linoleic acid metabolism	hiccup
path:00591	Linoleic acid metabolism	contact dermatitis
path:00591	Linoleic acid metabolism	vaginitis
path:00591	Linoleic acid metabolism	hematemesis
path:00591	Linoleic acid metabolism	vaginal hemorrhage
path:00591	Linoleic acid metabolism	urticaria
path:00591	Linoleic acid metabolism	hallucinations
path:00591	Linoleic acid metabolism	urinary frequency
path:00591	Linoleic acid metabolism	dry skin
path:00591	Linoleic acid metabolism	eructation
path:00591	Linoleic acid metabolism	glossitis
path:00591	Linoleic acid metabolism	cystitis
path:00591	Linoleic acid metabolism	ulcerative stomatitis
path:00591	Linoleic acid metabolism	dermatitis
path:00591	Linoleic acid metabolism	gastrointestinal hemorrhage
path:00591	Linoleic acid metabolism	erythema multiforme
path:00591	Linoleic acid metabolism	gastritis
path:00591	Linoleic acid metabolism	exfoliative dermatitis
path:00591	Linoleic acid metabolism	diplopia
path:00591	Linoleic acid metabolism	galactorrhea
path:00591	Linoleic acid metabolism	impotence
path:00591	Linoleic acid metabolism	ecchymosis
path:00591	Linoleic acid metabolism	dyskinesia
path:00591	Linoleic acid metabolism	coma
path:00591	Linoleic acid metabolism	toxic epidermal necrolysis
path:00591	Linoleic acid metabolism	urinary incontinence
path:00591	Linoleic acid metabolism	hypoxia
path:00591	Linoleic acid metabolism	hypothyroidism
path:00591	Linoleic acid metabolism	hypotension
path:00591	Linoleic acid metabolism	confusion
path:00591	Linoleic acid metabolism	hyperglycemia
path:00591	Linoleic acid metabolism	urinary retention
path:00591	Linoleic acid metabolism	hepatitis
path:00591	Linoleic acid metabolism	dysarthria
path:00591	Linoleic acid metabolism	tremor
path:00591	Linoleic acid metabolism	epistaxis
path:00591	Linoleic acid metabolism	herpes simplex
path:00670	One carbon pool by folate	leukopenia
path:00830	Retinol metabolism	diplopia
path:00830	Retinol metabolism	dysuria
path:00830	Retinol metabolism	shock

path:00830	Retinol metabolism	somnolence
path:00830	Retinol metabolism	dysarthria
path:00830	Retinol metabolism	pulmonary embolism
path:00830	Retinol metabolism	dry skin
path:00830	Retinol metabolism	SIADH
path:00830	Retinol metabolism	albuminuria
path:00830	Retinol metabolism	sleep disorder
path:00830	Retinol metabolism	eructation
path:00830	Retinol metabolism	amnesia
path:00830	Retinol metabolism	menstrual disorder
path:00830	Retinol metabolism	colitis
path:00830	Retinol metabolism	coma
path:00830	Retinol metabolism	hypoxia
path:00830	Retinol metabolism	hypotension
path:00830	Retinol metabolism	hiccup
path:00830	Retinol metabolism	laryngitis
path:00830	Retinol metabolism	breast pain
path:00830	Retinol metabolism	herpes simplex
path:00830	Retinol metabolism	liver function tests abnormal
path:00830	Retinol metabolism	confusion
path:00830	Retinol metabolism	otitis media
path:00830	Retinol metabolism	hepatitis
path:00830	Retinol metabolism	phlebitis
path:00830	Retinol metabolism	stomatitis
path:00830	Retinol metabolism	migraine
path:00830	Retinol metabolism	ataxia
path:00830	Retinol metabolism	neurosis
path:00830	Retinol metabolism	hallucinations
path:00830	Retinol metabolism	glossitis
path:00830	Retinol metabolism	gastrointestinal hemorrhage
path:00830	Retinol metabolism	dermatitis
path:00830	Retinol metabolism	gastritis
path:00830	Retinol metabolism	anemia
path:00830	Retinol metabolism	lymphadenopathy
path:00830	Retinol metabolism	tremor
path:00830	Retinol metabolism	urinary frequency
path:00830	Retinol metabolism	urinary incontinence
path:00830	Retinol metabolism	herpes zoster
path:00830	Retinol metabolism	tachycardia
path:00830	Retinol metabolism	ventricular fibrillation
path:00900	Terpenoid backbone biosynthesis	vertigo
path:00900	Terpenoid backbone biosynthesis	Dyspepsia
path:00900	Terpenoid backbone biosynthesis	arthralgia
path:00900	Terpenoid backbone biosynthesis	anemia
path:00900	Terpenoid backbone biosynthesis	angioedema
path:00900	Terpenoid backbone biosynthesis	Influenza
path:00900	Terpenoid backbone biosynthesis	hypersensitivity
path:00900	Terpenoid backbone biosynthesis	alopecia
path:00900	Terpenoid backbone biosynthesis	infection
path:00900	Terpenoid backbone biosynthesis	myalgia
path:00910	Nitrogen metabolism	anorexia
path:00980	Metabolism of xenobiotics by cytochrome P450	hallucinations
path:00980	Metabolism of xenobiotics by cytochrome P450	ataxia
path:00980	Metabolism of xenobiotics by cytochrome P450	diplopia
path:00980	Metabolism of xenobiotics by cytochrome P450	breast pain
path:00980	Metabolism of xenobiotics by cytochrome P450	hypotension

path:00980	Metabolism of xenobiotics by cytochrome P450	amnesia
path:00980	Metabolism of xenobiotics by cytochrome P450	glossitis
path:00980	Metabolism of xenobiotics by cytochrome P450	anemia
path:00980	Metabolism of xenobiotics by cytochrome P450	sleep disorder
path:00980	Metabolism of xenobiotics by cytochrome P450	otitis media
path:00980	Metabolism of xenobiotics by cytochrome P450	gastrointestinal hemorrhage
path:00980	Metabolism of xenobiotics by cytochrome P450	SIADH
path:00980	Metabolism of xenobiotics by cytochrome P450	herpes simplex
path:00980	Metabolism of xenobiotics by cytochrome P450	phlebitis
path:00980	Metabolism of xenobiotics by cytochrome P450	tremor
path:00980	Metabolism of xenobiotics by cytochrome P450	laryngitis
path:00980	Metabolism of xenobiotics by cytochrome P450	tinnitus
path:00980	Metabolism of xenobiotics by cytochrome P450	bradycardia
path:00980	Metabolism of xenobiotics by cytochrome P450	coma
path:00980	Metabolism of xenobiotics by cytochrome P450	dysuria
path:00980	Metabolism of xenobiotics by cytochrome P450	urinary incontinence
path:00980	Metabolism of xenobiotics by cytochrome P450	confusion
path:00980	Metabolism of xenobiotics by cytochrome P450	urinary frequency
path:00980	Metabolism of xenobiotics by cytochrome P450	eructation
path:00980	Metabolism of xenobiotics by cytochrome P450	hiccup
path:00980	Metabolism of xenobiotics by cytochrome P450	delirium
path:00980	Metabolism of xenobiotics by cytochrome P450	ventricular fibrillation
path:00980	Metabolism of xenobiotics by cytochrome P450	hyperglycemia
path:00980	Metabolism of xenobiotics by cytochrome P450	tachycardia
path:00980	Metabolism of xenobiotics by cytochrome P450	albuminuria
path:00980	Metabolism of xenobiotics by cytochrome P450	hypoxia
path:00982	Drug metabolism - cytochrome P450	laryngitis
path:00982	Drug metabolism - cytochrome P450	hypoxia
path:00982	Drug metabolism - cytochrome P450	breast enlargement
path:00982	Drug metabolism - cytochrome P450	urinary incontinence
path:00982	Drug metabolism - cytochrome P450	urinary frequency
path:00982	Drug metabolism - cytochrome P450	coma
path:00982	Drug metabolism - cytochrome P450	breast pain
path:00982	Drug metabolism - cytochrome P450	confusion
path:00982	Drug metabolism - cytochrome P450	eructation
path:00982	Drug metabolism - cytochrome P450	sleep disorder
path:00982	Drug metabolism - cytochrome P450	tremor
path:00982	Drug metabolism - cytochrome P450	SIADH
path:00982	Drug metabolism - cytochrome P450	ataxia
path:00982	Drug metabolism - cytochrome P450	somnolence
path:00982	Drug metabolism - cytochrome P450	tachycardia
path:00982	Drug metabolism - cytochrome P450	delirium
path:00982	Drug metabolism - cytochrome P450	hallucinations
path:00983	Drug metabolism - other enzymes	peripheral neuropathy
path:00983	Drug metabolism - other enzymes	pancytopenia
path:00983	Drug metabolism - other enzymes	chills
path:00983	Drug metabolism - other enzymes	sepsis
path:00983	Drug metabolism - other enzymes	confusion
path:00983	Drug metabolism - other enzymes	stomatitis
path:00983	Drug metabolism - other enzymes	dysarthria
path:00983	Drug metabolism - other enzymes	dysuria
path:00983	Drug metabolism - other enzymes	tremor
path:00983	Drug metabolism - other enzymes	hypoxia
path:00983	Drug metabolism - other enzymes	coma
path:02010	ABC transporters	dehydration
path:02010	ABC transporters	dermatitis

path:02010	ABC transporters	dyspnea
path:02010	ABC transporters	neutropenia
path:02010	ABC transporters	tumor
path:02010	ABC transporters	hepatitis
path:02010	ABC transporters	thrombocytopenia
path:02010	ABC transporters	vomiting
path:02010	ABC transporters	leukopenia
path:02010	ABC transporters	cardiac arrest
path:02010	ABC transporters	anemia
path:02010	ABC transporters	alopecia
path:03040	Spliceosome	photosensitivity
path:03040	Spliceosome	dry mouth
path:03040	Spliceosome	tremor
path:03040	Spliceosome	somnolence
path:03320	PPAR signaling pathway	jaundice
path:03320	PPAR signaling pathway	myalgia
path:03320	PPAR signaling pathway	hepatitis
path:03320	PPAR signaling pathway	rhabdomyolysis
path:04010	MAPK signaling pathway	leukopenia
path:04010	MAPK signaling pathway	petechiae
path:04010	MAPK signaling pathway	constitutional symptoms
path:04010	MAPK signaling pathway	swelling
path:04010	MAPK signaling pathway	nocturia
path:04010	MAPK signaling pathway	arthralgia
path:04010	MAPK signaling pathway	neutropenia
path:04010	MAPK signaling pathway	dehydration
path:04010	MAPK signaling pathway	pulmonary edema
path:04010	MAPK signaling pathway	constipation
path:04010	MAPK signaling pathway	thrombocytopenia
path:04010	MAPK signaling pathway	dyspnea
path:04010	MAPK signaling pathway	congestive heart failure
path:04010	MAPK signaling pathway	atrial fibrillation
path:04010	MAPK signaling pathway	neuropathy
path:04010	MAPK signaling pathway	muscle cramps
path:04010	MAPK signaling pathway	mouth ulceration
path:04010	MAPK signaling pathway	pleural effusion
path:04010	MAPK signaling pathway	cancer
path:04010	MAPK signaling pathway	sepsis
path:04010	MAPK signaling pathway	toxic epidermal necrolysis
path:04010	MAPK signaling pathway	weight loss
path:04010	MAPK signaling pathway	peripheral neuropathy
path:04010	MAPK signaling pathway	flushing
path:04010	MAPK signaling pathway	erythema multiforme
path:04010	MAPK signaling pathway	epistaxis
path:04010	MAPK signaling pathway	infection
path:04010	MAPK signaling pathway	renal failure
path:04010	MAPK signaling pathway	stomatitis
path:04010	MAPK signaling pathway	Stevens - Johnson syndrome
path:04010	MAPK signaling pathway	hemoptysis
path:04010	MAPK signaling pathway	anemia
path:04010	MAPK signaling pathway	gastrointestinal hemorrhage
path:04010	MAPK signaling pathway	heart failure
path:04012	ErbB signaling pathway	neutropenia
path:04012	ErbB signaling pathway	infection
path:04012	ErbB signaling pathway	dyspnea
path:04012	ErbB signaling pathway	stomatitis

path:04012	ErbB signaling pathway	hemorrhage
path:04020	Calcium signaling pathway	paralytic ileus
path:04020	Calcium signaling pathway	hypotension
path:04020	Calcium signaling pathway	palpitations
path:04020	Calcium signaling pathway	photophobia
path:04020	Calcium signaling pathway	nervousness
path:04020	Calcium signaling pathway	somnolence
path:04020	Calcium signaling pathway	cold extremities
path:04020	Calcium signaling pathway	neurosis
path:04020	Calcium signaling pathway	postural hypotension
path:04020	Calcium signaling pathway	constipation
path:04020	Calcium signaling pathway	grand mal
path:04020	Calcium signaling pathway	choreoathetosis
path:04020	Calcium signaling pathway	atrial fibrillation
path:04020	Calcium signaling pathway	tremor
path:04020	Calcium signaling pathway	agitation
path:04020	Calcium signaling pathway	heart block
path:04020	Calcium signaling pathway	nightmares
path:04020	Calcium signaling pathway	tardive dyskinesia
path:04020	Calcium signaling pathway	abnormal vision
path:04020	Calcium signaling pathway	blurred vision
path:04020	Calcium signaling pathway	Parkinson
path:04020	Calcium signaling pathway	paranoia
path:04020	Calcium signaling pathway	AV block
path:04020	Calcium signaling pathway	bradycardia
path:04020	Calcium signaling pathway	gynecomastia
path:04020	Calcium signaling pathway	tachycardia
path:04020	Calcium signaling pathway	dry mouth
path:04020	Calcium signaling pathway	psoriasis
path:04020	Calcium signaling pathway	priapism
path:04020	Calcium signaling pathway	fatigue
path:04020	Calcium signaling pathway	torticollis
path:04020	Calcium signaling pathway	increased salivation
path:04020	Calcium signaling pathway	sweating
path:04020	Calcium signaling pathway	dyskinesia
path:04020	Calcium signaling pathway	syncope
path:04020	Calcium signaling pathway	keratitis
path:04020	Calcium signaling pathway	lactic dehydrogenase increased
path:04020	Calcium signaling pathway	urinary frequency
path:04020	Calcium signaling pathway	eczema
path:04020	Calcium signaling pathway	supraventricular extrasystoles
path:04020	Calcium signaling pathway	insomnia
path:04020	Calcium signaling pathway	impotence
path:04020	Calcium signaling pathway	serotonin syndrome
path:04020	Calcium signaling pathway	galactorrhea
path:04020	Calcium signaling pathway	ventricular tachycardia
path:04020	Calcium signaling pathway	weight gain
path:04020	Calcium signaling pathway	nasal congestion
path:04020	Calcium signaling pathway	pyuria
path:04020	Calcium signaling pathway	urinary retention
path:04060	Cytokine-cytokine receptor interaction	hepatitis
path:04060	Cytokine-cytokine receptor interaction	pancreatitis
path:04060	Cytokine-cytokine receptor interaction	eczema
path:04060	Cytokine-cytokine receptor interaction	epistaxis
path:04060	Cytokine-cytokine receptor interaction	upper respiratory tract infection
path:04060	Cytokine-cytokine receptor interaction	pancytopenia

path:04060	Cytokine-cytokine receptor interaction	muscle cramps
path:04060	Cytokine-cytokine receptor interaction	conjunctivitis
path:04060	Cytokine-cytokine receptor interaction	neutropenia
path:04060	Cytokine-cytokine receptor interaction	myalgia
path:04060	Cytokine-cytokine receptor interaction	Dyspepsia
path:04060	Cytokine-cytokine receptor interaction	Influenza
path:04060	Cytokine-cytokine receptor interaction	arthralgia
path:04060	Cytokine-cytokine receptor interaction	infection
path:04060	Cytokine-cytokine receptor interaction	abdominal pain
path:04062	Chemokine signaling pathway	lupus
path:04062	Chemokine signaling pathway	dyspnea
path:04062	Chemokine signaling pathway	muscle cramps
path:04080	Neuroactive ligand-receptor interaction	glaucoma
path:04080	Neuroactive ligand-receptor interaction	torticollis
path:04080	Neuroactive ligand-receptor interaction	increased salivation
path:04080	Neuroactive ligand-receptor interaction	impotence
path:04080	Neuroactive ligand-receptor interaction	galactorrhea
path:04080	Neuroactive ligand-receptor interaction	ventricular extrasystoles
path:04080	Neuroactive ligand-receptor interaction	hypoventilation
path:04080	Neuroactive ligand-receptor interaction	fecal impaction
path:04080	Neuroactive ligand-receptor interaction	hypotension
path:04080	Neuroactive ligand-receptor interaction	keratitis
path:04080	Neuroactive ligand-receptor interaction	goiter
path:04080	Neuroactive ligand-receptor interaction	grand mal
path:04080	Neuroactive ligand-receptor interaction	hallucinations
path:04080	Neuroactive ligand-receptor interaction	paralytic ileus
path:04080	Neuroactive ligand-receptor interaction	heart block
path:04080	Neuroactive ligand-receptor interaction	hyperacusis
path:04080	Neuroactive ligand-receptor interaction	hiccup
path:04080	Neuroactive ligand-receptor interaction	paranoia
path:04080	Neuroactive ligand-receptor interaction	wheezing
path:04080	Neuroactive ligand-receptor interaction	tremor
path:04080	Neuroactive ligand-receptor interaction	hypothermia
path:04080	Neuroactive ligand-receptor interaction	dysarthria
path:04080	Neuroactive ligand-receptor interaction	urinary urgency
path:04080	Neuroactive ligand-receptor interaction	tardive dyskinesia
path:04080	Neuroactive ligand-receptor interaction	nervousness
path:04080	Neuroactive ligand-receptor interaction	diplopia
path:04080	Neuroactive ligand-receptor interaction	drug dependence
path:04080	Neuroactive ligand-receptor interaction	palpitations
path:04080	Neuroactive ligand-receptor interaction	dry mouth
path:04080	Neuroactive ligand-receptor interaction	tachycardia
path:04080	Neuroactive ligand-receptor interaction	insomnia
path:04080	Neuroactive ligand-receptor interaction	urinary retention
path:04080	Neuroactive ligand-receptor interaction	urinary hesitancy
path:04080	Neuroactive ligand-receptor interaction	urinary incontinence
path:04080	Neuroactive ligand-receptor interaction	dyskinesia
path:04080	Neuroactive ligand-receptor interaction	confusion
path:04080	Neuroactive ligand-receptor interaction	dysmenorrhea
path:04080	Neuroactive ligand-receptor interaction	delirium
path:04080	Neuroactive ligand-receptor interaction	laryngitis
path:04080	Neuroactive ligand-receptor interaction	labyrinthitis
path:04080	Neuroactive ligand-receptor interaction	nightmares
path:04080	Neuroactive ligand-receptor interaction	Toothache
path:04080	Neuroactive ligand-receptor interaction	nasal congestion
path:04080	Neuroactive ligand-receptor interaction	blurred vision

path:04080	Neuroactive ligand-receptor interaction	syncope
path:04080	Neuroactive ligand-receptor interaction	ventricular tachycardia
path:04080	Neuroactive ligand-receptor interaction	spasm
path:04080	Neuroactive ligand-receptor interaction	ataxia
path:04080	Neuroactive ligand-receptor interaction	stridor
path:04080	Neuroactive ligand-receptor interaction	seizures
path:04080	Neuroactive ligand-receptor interaction	sweating
path:04080	Neuroactive ligand-receptor interaction	serotonin syndrome
path:04080	Neuroactive ligand-receptor interaction	personality disorder
path:04080	Neuroactive ligand-receptor interaction	supraventricular extrasystoles
path:04080	Neuroactive ligand-receptor interaction	anxiety
path:04080	Neuroactive ligand-receptor interaction	agitation
path:04080	Neuroactive ligand-receptor interaction	postural hypotension
path:04080	Neuroactive ligand-receptor interaction	abnormal gait
path:04080	Neuroactive ligand-receptor interaction	abnormal vision
path:04080	Neuroactive ligand-receptor interaction	photophobia
path:04080	Neuroactive ligand-receptor interaction	Parkinson
path:04080	Neuroactive ligand-receptor interaction	sneezing
path:04080	Neuroactive ligand-receptor interaction	priapism
path:04080	Neuroactive ligand-receptor interaction	apnea
path:04080	Neuroactive ligand-receptor interaction	voice alteration
path:04080	Neuroactive ligand-receptor interaction	psoriasis
path:04080	Neuroactive ligand-receptor interaction	bradycardia
path:04080	Neuroactive ligand-receptor interaction	abuse
path:04080	Neuroactive ligand-receptor interaction	cardiac arrest
path:04080	Neuroactive ligand-receptor interaction	pyelonephritis
path:04080	Neuroactive ligand-receptor interaction	somnolence
path:04114	Oocyte meiosis	gynecomastia
path:04115	p53 signaling pathway	renal failure
path:04115	p53 signaling pathway	dyspnea
path:04115	p53 signaling pathway	leukopenia
path:04115	p53 signaling pathway	neutropenia
path:04115	p53 signaling pathway	anorexia
path:04115	p53 signaling pathway	congestive heart failure
path:04115	p53 signaling pathway	stomatitis
path:04115	p53 signaling pathway	chills
path:04115	p53 signaling pathway	asthenia
path:04115	p53 signaling pathway	anemia
path:04115	p53 signaling pathway	hypersensitivity
path:04115	p53 signaling pathway	diarrhea
path:04115	p53 signaling pathway	increased sweating
path:04115	p53 signaling pathway	neuropathy
path:04115	p53 signaling pathway	thrombocytopenia
path:04144	Endocytosis	congestive heart failure
path:04144	Endocytosis	decreased libido
path:04144	Endocytosis	lightheadedness
path:04144	Endocytosis	dizziness
path:04144	Endocytosis	heart block
path:04144	Endocytosis	bradycardia
path:04144	Endocytosis	flushing
path:04144	Endocytosis	pain
path:04144	Endocytosis	nausea
path:04144	Endocytosis	psoriasis
path:04144	Endocytosis	anxiety
path:04144	Endocytosis	dry eyes
path:04144	Endocytosis	fatigue

path:04144	Endocytosis	pulmonary edema
path:04144	Endocytosis	AV block
path:04144	Endocytosis	mental depression
path:04144	Endocytosis	sore throat
path:04144	Endocytosis	memory loss
path:04144	Endocytosis	nightmares
path:04144	Endocytosis	insomnia
path:04144	Endocytosis	hypokalemia
path:04144	Endocytosis	bronchospasm
path:04144	Endocytosis	fever
path:04150	mTOR signaling pathway	dyspnea
path:04210	Apoptosis	cough
path:04210	Apoptosis	thrombocytopenia
path:04210	Apoptosis	pneumonia
path:04210	Apoptosis	hypersensitivity
path:04210	Apoptosis	neutropenia
path:04210	Apoptosis	heart failure
path:04210	Apoptosis	dyspnea
path:04210	Apoptosis	Sinusitis
path:04210	Apoptosis	upper respiratory tract infection
path:04210	Apoptosis	anemia
path:04210	Apoptosis	fever
path:04210	Apoptosis	pancytopenia
path:04210	Apoptosis	infection
path:04210	Apoptosis	peripheral neuropathy
path:04210	Apoptosis	stomatitis
path:04210	Apoptosis	alopecia
path:04210	Apoptosis	conjunctivitis
path:04210	Apoptosis	edema
path:04210	Apoptosis	hematuria
path:04210	Apoptosis	congestive heart failure
path:04210	Apoptosis	leukopenia
path:04210	Apoptosis	anorexia
path:04210	Apoptosis	arthralgia
path:04270	Vascular smooth muscle contraction	postural hypotension
path:04270	Vascular smooth muscle contraction	amenorrhea
path:04270	Vascular smooth muscle contraction	impotence
path:04270	Vascular smooth muscle contraction	increased salivation
path:04270	Vascular smooth muscle contraction	hyperglycemia
path:04270	Vascular smooth muscle contraction	chest pain
path:04270	Vascular smooth muscle contraction	tardive dyskinesia
path:04270	Vascular smooth muscle contraction	paralytic ileus
path:04270	Vascular smooth muscle contraction	dry mouth
path:04270	Vascular smooth muscle contraction	nasal congestion
path:04270	Vascular smooth muscle contraction	Parkinson
path:04270	Vascular smooth muscle contraction	priapism
path:04270	Vascular smooth muscle contraction	torticollis
path:04270	Vascular smooth muscle contraction	dyskinesia
path:04270	Vascular smooth muscle contraction	tachycardia
path:04270	Vascular smooth muscle contraction	glycosuria
path:04270	Vascular smooth muscle contraction	spasm
path:04270	Vascular smooth muscle contraction	palpitations
path:04270	Vascular smooth muscle contraction	gynecomastia
path:04310	Wnt signaling pathway	anemia
path:04350	TGF-beta signaling pathway	anorexia
path:04370	VEGF signaling pathway	sepsis

path:04370	VEGF signaling pathway	aplastic anemia
path:04370	VEGF signaling pathway	alopecia
path:04370	VEGF signaling pathway	infection
path:04370	VEGF signaling pathway	rectal hemorrhage
path:04370	VEGF signaling pathway	dysuria
path:04370	VEGF signaling pathway	gastrointestinal hemorrhage
path:04370	VEGF signaling pathway	dyspnea
path:04370	VEGF signaling pathway	eosinophilia
path:04370	VEGF signaling pathway	anemia
path:04370	VEGF signaling pathway	proteinuria
path:04370	VEGF signaling pathway	leukopenia
path:04370	VEGF signaling pathway	pruritus
path:04370	VEGF signaling pathway	jaundice
path:04370	VEGF signaling pathway	interstitial nephritis
path:04370	VEGF signaling pathway	ecchymosis
path:04370	VEGF signaling pathway	Dyspepsia
path:04370	VEGF signaling pathway	eructation
path:04370	VEGF signaling pathway	erythema multiforme
path:04370	VEGF signaling pathway	thrombocytopenia
path:04370	VEGF signaling pathway	purpura
path:04370	VEGF signaling pathway	toxic epidermal necrolysis
path:04370	VEGF signaling pathway	angioedema
path:04370	VEGF signaling pathway	exfoliative dermatitis
path:04370	VEGF signaling pathway	gastritis
path:04370	VEGF signaling pathway	epistaxis
path:04370	VEGF signaling pathway	renal failure
path:04370	VEGF signaling pathway	melena
path:04370	VEGF signaling pathway	flatulence
path:04370	VEGF signaling pathway	esophagitis
path:04370	VEGF signaling pathway	hepatitis
path:04370	VEGF signaling pathway	colitis
path:04370	VEGF signaling pathway	hemolytic anemia
path:04370	VEGF signaling pathway	pancreatitis
path:04370	VEGF signaling pathway	vertigo
path:04370	VEGF signaling pathway	pancytopenia
path:04370	VEGF signaling pathway	peripheral neuropathy
path:04370	VEGF signaling pathway	hepatic failure
path:04370	VEGF signaling pathway	photosensitivity
path:04370	VEGF signaling pathway	stomatitis
path:04370	VEGF signaling pathway	hematemesis
path:04370	VEGF signaling pathway	Stevens - Johnson syndrome
path:04370	VEGF signaling pathway	peptic ulcer
path:04370	VEGF signaling pathway	ulcer
path:04370	VEGF signaling pathway	pneumonia
path:04370	VEGF signaling pathway	hearing loss
path:04370	VEGF signaling pathway	vasculitis
path:04370	VEGF signaling pathway	congestive heart failure
path:04370	VEGF signaling pathway	conjunctivitis
path:04370	VEGF signaling pathway	dry skin
path:04370	VEGF signaling pathway	hematuria
path:04370	VEGF signaling pathway	asthenia
path:04510	Focal adhesion	erythema multiforme
path:04510	Focal adhesion	stomatitis
path:04510	Focal adhesion	pancreatitis
path:04510	Focal adhesion	weight loss
path:04510	Focal adhesion	gastritis

path:04510	Focal adhesion	congestive heart failure
path:04510	Focal adhesion	dyspnea
path:04510	Focal adhesion	hemorrhage
path:04510	Focal adhesion	flushing
path:04510	Focal adhesion	exfoliative dermatitis
path:04510	Focal adhesion	anemia
path:04510	Focal adhesion	constitutional symptoms
path:04510	Focal adhesion	peripheral edema
path:04510	Focal adhesion	Vascular Disorders
path:04510	Focal adhesion	epistaxis
path:04520	Adherens junction	anemia
path:04520	Adherens junction	rash
path:04520	Adherens junction	infection
path:04520	Adherens junction	arthralgia
path:04520	Adherens junction	dyspnea
path:04520	Adherens junction	edema
path:04520	Adherens junction	asthenia
path:04520	Adherens junction	Dyspepsia
path:04520	Adherens junction	anorexia
path:04520	Adherens junction	diarrhea
path:04520	Adherens junction	constipation
path:04540	Gap junction	palpitations
path:04540	Gap junction	galactorrhea
path:04540	Gap junction	leukocytosis
path:04540	Gap junction	personality disorder
path:04540	Gap junction	hypotension
path:04540	Gap junction	skin ulcer
path:04540	Gap junction	insomnia
path:04540	Gap junction	suicide attempt
path:04540	Gap junction	breast enlargement
path:04540	Gap junction	edema
path:04540	Gap junction	congestive heart failure
path:04540	Gap junction	exfoliative dermatitis
path:04540	Gap junction	paranoia
path:04540	Gap junction	Parkinson
path:04540	Gap junction	cholecystitis
path:04540	Gap junction	paralytic ileus
path:04540	Gap junction	cardiomyopathy
path:04540	Gap junction	flushing
path:04540	Gap junction	increased salivation
path:04540	Gap junction	cold extremities
path:04540	Gap junction	peripheral edema
path:04540	Gap junction	fecal incontinence
path:04540	Gap junction	torticollis
path:04540	Gap junction	fatigue
path:04540	Gap junction	amenorrhea
path:04540	Gap junction	ventricular fibrillation
path:04540	Gap junction	ischemic colitis
path:04540	Gap junction	syncope
path:04540	Gap junction	bundle branch block
path:04540	Gap junction	impotence
path:04540	Gap junction	shortness of breath
path:04540	Gap junction	bronchospasm
path:04540	Gap junction	tachycardia
path:04540	Gap junction	weight gain
path:04540	Gap junction	confusion

path:04540	Gap junction	weakness
path:04540	Gap junction	ventricular tachycardia
path:04540	Gap junction	SIADH
path:04540	Gap junction	hypothyroidism
path:04540	Gap junction	seizures
path:04540	Gap junction	tardive dyskinesia
path:04540	Gap junction	dysphagia
path:04540	Gap junction	dyskinesia
path:04540	Gap junction	neuralgia
path:04540	Gap junction	mental depression
path:04540	Gap junction	menorrhagia
path:04540	Gap junction	hyperhidrosis
path:04540	Gap junction	anxiety
path:04540	Gap junction	psychosis
path:04540	Gap junction	tremor
path:04540	Gap junction	neurosis
path:04540	Gap junction	hypokalemia
path:04540	Gap junction	agranulocytosis
path:04540	Gap junction	gynecomastia
path:04540	Gap junction	ataxia
path:04540	Gap junction	dysmenorrhea
path:04540	Gap junction	alkaline phosphatase increased
path:04540	Gap junction	urinary incontinence
path:04540	Gap junction	sweating
path:04540	Gap junction	weight loss
path:04540	Gap junction	priapism
path:04540	Gap junction	urinary retention
path:04540	Gap junction	postural hypotension
path:04540	Gap junction	arrhythmia
path:04540	Gap junction	asthma
path:04540	Gap junction	nasal congestion
path:04540	Gap junction	heart block
path:04540	Gap junction	dizziness
path:04540	Gap junction	spasm
path:04540	Gap junction	hallucinations
path:04540	Gap junction	psoriasis
path:04540	Gap junction	dry mouth
path:04540	Gap junction	hypertension
path:04540	Gap junction	abnormal gait
path:04540	Gap junction	bradycardia
path:04540	Gap junction	eczema
path:04540	Gap junction	constipation
path:04540	Gap junction	delirium
path:04540	Gap junction	nightmares
path:04540	Gap junction	hypoglycemia
path:04540	Gap junction	goiter
path:04540	Gap junction	heart failure
path:04540	Gap junction	dyspnea
path:04540	Gap junction	lightheadedness
path:04540	Gap junction	deafness
path:04540	Gap junction	decreased libido
path:04540	Gap junction	dehydration
path:04540	Gap junction	AV block
path:04540	Gap junction	sore throat
path:04540	Gap junction	grand mal
path:04540	Gap junction	somnolence

path:04540	Gap junction	lupus
path:04540	Gap junction	blepharitis
path:04540	Gap junction	purpura
path:04610	Complement and coagulation cascades	vasculitis
path:04610	Complement and coagulation cascades	flatulence
path:04610	Complement and coagulation cascades	anemia
path:04610	Complement and coagulation cascades	acute renal failure
path:04610	Complement and coagulation cascades	hepatitis
path:04610	Complement and coagulation cascades	urticaria
path:04610	Complement and coagulation cascades	diarrhea
path:04610	Complement and coagulation cascades	pruritus
path:04610	Complement and coagulation cascades	thrombocytopenia
path:04610	Complement and coagulation cascades	renal failure
path:04614	Renin-angiotensin system	hyperuricemia
path:04614	Renin-angiotensin system	abdominal pain
path:04614	Renin-angiotensin system	weakness
path:04614	Renin-angiotensin system	hemolytic anemia
path:04614	Renin-angiotensin system	alopecia
path:04614	Renin-angiotensin system	hyponatremia
path:04614	Renin-angiotensin system	flushing
path:04614	Renin-angiotensin system	headache
path:04614	Renin-angiotensin system	impotence
path:04614	Renin-angiotensin system	hyperkalemia
path:04614	Renin-angiotensin system	hyperglycemia
path:04614	Renin-angiotensin system	gout
path:04614	Renin-angiotensin system	Stevens - Johnson syndrome
path:04614	Renin-angiotensin system	hepatitis
path:04614	Renin-angiotensin system	glycosuria
path:04614	Renin-angiotensin system	dermatitis
path:04614	Renin-angiotensin system	tinnitus
path:04614	Renin-angiotensin system	vasculitis
path:04614	Renin-angiotensin system	myalgia
path:04614	Renin-angiotensin system	pancreatitis
path:04614	Renin-angiotensin system	AV block
path:04614	Renin-angiotensin system	renal failure
path:04614	Renin-angiotensin system	palpitations
path:04614	Renin-angiotensin system	arthralgia
path:04614	Renin-angiotensin system	thrombocytopenia
path:04614	Renin-angiotensin system	bronchospasm
path:04614	Renin-angiotensin system	rash
path:04614	Renin-angiotensin system	Dyspepsia
path:04614	Renin-angiotensin system	urinary frequency
path:04614	Renin-angiotensin system	paresthesia
path:04614	Renin-angiotensin system	photosensitivity
path:04614	Renin-angiotensin system	dyspnea
path:04614	Renin-angiotensin system	purpura
path:04614	Renin-angiotensin system	constipation
path:04614	Renin-angiotensin system	muscle cramps
path:04614	Renin-angiotensin system	angina pectoris
path:04614	Renin-angiotensin system	exfoliative dermatitis
path:04614	Renin-angiotensin system	postural hypotension
path:04614	Renin-angiotensin system	asthenia
path:04614	Renin-angiotensin system	dizziness
path:04614	Renin-angiotensin system	fatigue
path:04614	Renin-angiotensin system	rhinitis
path:04614	Renin-angiotensin system	angioedema

path:04614	Renin-angiotensin system	diarrhea
path:04614	Renin-angiotensin system	erythema multiforme
path:04614	Renin-angiotensin system	vertigo
path:04614	Renin-angiotensin system	rhabdomyolysis
path:04614	Renin-angiotensin system	syncope
path:04614	Renin-angiotensin system	chest pain
path:04614	Renin-angiotensin system	renal insufficiency
path:04620	Toll-like receptor signaling pathway	purpura
path:04620	Toll-like receptor signaling pathway	anemia
path:04620	Toll-like receptor signaling pathway	anorexia
path:04620	Toll-like receptor signaling pathway	abdominal pain
path:04620	Toll-like receptor signaling pathway	muscle cramps
path:04620	Toll-like receptor signaling pathway	fever
path:04620	Toll-like receptor signaling pathway	arthritis
path:04620	Toll-like receptor signaling pathway	neutropenia
path:04620	Toll-like receptor signaling pathway	conjunctivitis
path:04620	Toll-like receptor signaling pathway	pancreatitis
path:04620	Toll-like receptor signaling pathway	upper respiratory tract infection
path:04620	Toll-like receptor signaling pathway	angioedema
path:04620	Toll-like receptor signaling pathway	epistaxis
path:04621	NOD-like receptor signaling pathway	Dyspepsia
path:04621	NOD-like receptor signaling pathway	dermatitis
path:04621	NOD-like receptor signaling pathway	neutropenia
path:04621	NOD-like receptor signaling pathway	infection
path:04621	NOD-like receptor signaling pathway	myalgia
path:04621	NOD-like receptor signaling pathway	photosensitivity
path:04621	NOD-like receptor signaling pathway	Pharyngitis
path:04621	NOD-like receptor signaling pathway	malaise
path:04621	NOD-like receptor signaling pathway	hypersensitivity
path:04621	NOD-like receptor signaling pathway	muscle cramps
path:04621	NOD-like receptor signaling pathway	arthritis
path:04621	NOD-like receptor signaling pathway	alopecia
path:04621	NOD-like receptor signaling pathway	flatulence
path:04621	NOD-like receptor signaling pathway	peripheral neuropathy
path:04621	NOD-like receptor signaling pathway	fever
path:04621	NOD-like receptor signaling pathway	hepatitis
path:04621	NOD-like receptor signaling pathway	anemia
path:04621	NOD-like receptor signaling pathway	rhinitis
path:04621	NOD-like receptor signaling pathway	abdominal pain
path:04621	NOD-like receptor signaling pathway	stomatitis
path:04621	NOD-like receptor signaling pathway	epistaxis
path:04621	NOD-like receptor signaling pathway	toxic epidermal necrolysis
path:04621	NOD-like receptor signaling pathway	vomiting
path:04621	NOD-like receptor signaling pathway	pancreatitis
path:04621	NOD-like receptor signaling pathway	thrombocytopenia
path:04621	NOD-like receptor signaling pathway	jaundice
path:04621	NOD-like receptor signaling pathway	upper respiratory tract infection
path:04621	NOD-like receptor signaling pathway	angioedema
path:04621	NOD-like receptor signaling pathway	edema
path:04621	NOD-like receptor signaling pathway	eczema
path:04621	NOD-like receptor signaling pathway	anorexia
path:04621	NOD-like receptor signaling pathway	purpura
path:04621	NOD-like receptor signaling pathway	conjunctivitis
path:04621	NOD-like receptor signaling pathway	dyspnea
path:04621	NOD-like receptor signaling pathway	Sinusitis
path:04622	RIG-I-like receptor signaling pathway	malaise

path:04622	RIG-I-like receptor signaling pathway	abdominal pain
path:04622	RIG-I-like receptor signaling pathway	conjunctivitis
path:04622	RIG-I-like receptor signaling pathway	dyspnea
path:04622	RIG-I-like receptor signaling pathway	infection
path:04622	RIG-I-like receptor signaling pathway	urinary tract infection
path:04622	RIG-I-like receptor signaling pathway	hypersensitivity
path:04622	RIG-I-like receptor signaling pathway	fever
path:04622	RIG-I-like receptor signaling pathway	Dyspepsia
path:04622	RIG-I-like receptor signaling pathway	anorexia
path:04622	RIG-I-like receptor signaling pathway	upper respiratory tract infection
path:04622	RIG-I-like receptor signaling pathway	epistaxis
path:04622	RIG-I-like receptor signaling pathway	Sinusitis
path:04630	Jak-STAT signaling pathway	upper respiratory tract infection
path:04630	Jak-STAT signaling pathway	Dyspepsia
path:04630	Jak-STAT signaling pathway	hepatitis
path:04630	Jak-STAT signaling pathway	Sinusitis
path:04630	Jak-STAT signaling pathway	fever
path:04630	Jak-STAT signaling pathway	insomnia
path:04630	Jak-STAT signaling pathway	edema
path:04630	Jak-STAT signaling pathway	angioedema
path:04630	Jak-STAT signaling pathway	Pharyngitis
path:04630	Jak-STAT signaling pathway	abdominal pain
path:04630	Jak-STAT signaling pathway	myalgia
path:04640	Hematopoietic cell lineage	hepatitis
path:04640	Hematopoietic cell lineage	hemorrhage
path:04640	Hematopoietic cell lineage	pancreatitis
path:04640	Hematopoietic cell lineage	upper respiratory tract infection
path:04640	Hematopoietic cell lineage	pancytopenia
path:04640	Hematopoietic cell lineage	thrombocytopenia
path:04650	Natural killer cell mediated cytotoxicity	ulcer
path:04650	Natural killer cell mediated cytotoxicity	fever
path:04650	Natural killer cell mediated cytotoxicity	cough
path:04650	Natural killer cell mediated cytotoxicity	flushing
path:04650	Natural killer cell mediated cytotoxicity	alopecia
path:04650	Natural killer cell mediated cytotoxicity	epistaxis
path:04650	Natural killer cell mediated cytotoxicity	dyspnea
path:04650	Natural killer cell mediated cytotoxicity	abdominal pain
path:04650	Natural killer cell mediated cytotoxicity	conjunctivitis
path:04650	Natural killer cell mediated cytotoxicity	hypokalemia
path:04650	Natural killer cell mediated cytotoxicity	hypersensitivity
path:04650	Natural killer cell mediated cytotoxicity	Influenza
path:04650	Natural killer cell mediated cytotoxicity	neutropenia
path:04650	Natural killer cell mediated cytotoxicity	infection
path:04660	T cell receptor signaling pathway	anorexia
path:04660	T cell receptor signaling pathway	angioedema
path:04660	T cell receptor signaling pathway	upper respiratory tract infection
path:04660	T cell receptor signaling pathway	pneumonia
path:04660	T cell receptor signaling pathway	Dyspepsia
path:04660	T cell receptor signaling pathway	gastroenteritis
path:04660	T cell receptor signaling pathway	epistaxis
path:04660	T cell receptor signaling pathway	infection
path:04660	T cell receptor signaling pathway	muscle cramps
path:04660	T cell receptor signaling pathway	purpura
path:04660	T cell receptor signaling pathway	hepatitis
path:04660	T cell receptor signaling pathway	arthralgia
path:04660	T cell receptor signaling pathway	dermatitis

path:04660	T cell receptor signaling pathway	Influenza
path:04660	T cell receptor signaling pathway	conjunctivitis
path:04660	T cell receptor signaling pathway	pancreatitis
path:04660	T cell receptor signaling pathway	exfoliative dermatitis
path:04660	T cell receptor signaling pathway	hypersensitivity
path:04660	T cell receptor signaling pathway	abdominal pain
path:04660	T cell receptor signaling pathway	fever
path:04660	T cell receptor signaling pathway	Sinusitis
path:04662	B cell receptor signaling pathway	arthralgia
path:04662	B cell receptor signaling pathway	dyspnea
path:04664	Fc epsilon RI signaling pathway	infection
path:04664	Fc epsilon RI signaling pathway	pancreatitis
path:04664	Fc epsilon RI signaling pathway	erythema multiforme
path:04664	Fc epsilon RI signaling pathway	conjunctivitis
path:04664	Fc epsilon RI signaling pathway	Influenza
path:04664	Fc epsilon RI signaling pathway	epistaxis
path:04664	Fc epsilon RI signaling pathway	upper respiratory tract infection
path:04664	Fc epsilon RI signaling pathway	peripheral neuropathy
path:04664	Fc epsilon RI signaling pathway	Dyspepsia
path:04670	Leukocyte transendothelial migration	flushing
path:04672	Intestinal immune network for IgA production	infection
path:04672	Intestinal immune network for IgA production	fever
path:04672	Intestinal immune network for IgA production	Pharyngitis
path:04672	Intestinal immune network for IgA production	angioedema
path:04672	Intestinal immune network for IgA production	edema
path:04672	Intestinal immune network for IgA production	Dyspepsia
path:04720	Long-term potentiation	postural hypotension
path:04720	Long-term potentiation	flushing
path:04720	Long-term potentiation	dyskinesia
path:04720	Long-term potentiation	gynecomastia
path:04720	Long-term potentiation	nasal congestion
path:04722	Neurotrophin signaling pathway	exfoliative dermatitis
path:04722	Neurotrophin signaling pathway	eosinophilia
path:04722	Neurotrophin signaling pathway	erythema multiforme
path:04722	Neurotrophin signaling pathway	leukopenia
path:04722	Neurotrophin signaling pathway	gynecomastia
path:04722	Neurotrophin signaling pathway	pancytopenia
path:04722	Neurotrophin signaling pathway	anemia
path:04722	Neurotrophin signaling pathway	jaundice
path:04722	Neurotrophin signaling pathway	purpura
path:04722	Neurotrophin signaling pathway	peripheral neuropathy
path:04730	Long-term depression	gastrointestinal hemorrhage
path:04730	Long-term depression	shock
path:04730	Long-term depression	nocturia
path:04742	Taste transduction	dyskinesia
path:04742	Taste transduction	increased sweating
path:04742	Taste transduction	impotence
path:04742	Taste transduction	nystagmus
path:04742	Taste transduction	dyspnea
path:04742	Taste transduction	insomnia
path:04742	Taste transduction	nervousness
path:04742	Taste transduction	urinary retention
path:04742	Taste transduction	lupus
path:04742	Taste transduction	postural hypotension
path:04742	Taste transduction	syncope
path:04742	Taste transduction	tremor

path:04742	Taste transduction	bradycardia
path:04742	Taste transduction	AV block
path:04742	Taste transduction	ataxia
path:04742	Taste transduction	cardiac arrest
path:04742	Taste transduction	chest pain
path:04742	Taste transduction	photophobia
path:04742	Taste transduction	apnea
path:04742	Taste transduction	somnolence
path:04742	Taste transduction	paralysis
path:04742	Taste transduction	amnesia
path:04742	Taste transduction	shock
path:04742	Taste transduction	psychosis
path:04810	Regulation of actin cytoskeleton	urinary retention
path:04810	Regulation of actin cytoskeleton	gingivitis
path:04810	Regulation of actin cytoskeleton	breast enlargement
path:04810	Regulation of actin cytoskeleton	tardive dyskinesia
path:04810	Regulation of actin cytoskeleton	weight loss
path:04810	Regulation of actin cytoskeleton	alkaline phosphatase increased
path:04810	Regulation of actin cytoskeleton	gastroesophageal reflux
path:04810	Regulation of actin cytoskeleton	galactorrhea
path:04810	Regulation of actin cytoskeleton	dyskinesia
path:04810	Regulation of actin cytoskeleton	amenorrhea
path:04810	Regulation of actin cytoskeleton	dysphagia
path:04810	Regulation of actin cytoskeleton	rhabdomyolysis
path:04810	Regulation of actin cytoskeleton	torticollis
path:04810	Regulation of actin cytoskeleton	priapism
path:04810	Regulation of actin cytoskeleton	increased salivation
path:04810	Regulation of actin cytoskeleton	eosinophilia
path:04810	Regulation of actin cytoskeleton	blepharitis
path:04810	Regulation of actin cytoskeleton	gynecomastia
path:04810	Regulation of actin cytoskeleton	glycosuria
path:04810	Regulation of actin cytoskeleton	urinary urgency
path:04810	Regulation of actin cytoskeleton	tachycardia
path:04810	Regulation of actin cytoskeleton	paralytic ileus
path:04810	Regulation of actin cytoskeleton	delirium
path:04810	Regulation of actin cytoskeleton	constipation
path:04810	Regulation of actin cytoskeleton	Parkinson
path:04910	Insulin signaling pathway	exfoliative dermatitis
path:04910	Insulin signaling pathway	gynecomastia
path:04912	GnRH signaling pathway	postural hypotension
path:04912	GnRH signaling pathway	gynecomastia
path:04912	GnRH signaling pathway	flushing
path:04912	GnRH signaling pathway	paralytic ileus
path:04914	Progesterone-mediated oocyte maturation	hypokalemia
path:04914	Progesterone-mediated oocyte maturation	pleural effusion
path:04916	Melanogenesis	nasal congestion
path:04916	Melanogenesis	lupus
path:04916	Melanogenesis	gynecomastia
path:04920	Adipocytokine signaling pathway	neutropenia
path:04920	Adipocytokine signaling pathway	hepatitis
path:04930	Type II diabetes mellitus	nocturia
path:04930	Type II diabetes mellitus	hepatitis
path:04960	Aldosterone-regulated sodium reabsorption	increased sweating
path:04960	Aldosterone-regulated sodium reabsorption	thrombocytopenia
path:04960	Aldosterone-regulated sodium reabsorption	diplopia
path:04960	Aldosterone-regulated sodium reabsorption	jaundice

path:04960	Aldosterone-regulated sodium reabsorption	hyponatremia
path:04960	Aldosterone-regulated sodium reabsorption	hypoglycemia
path:04960	Aldosterone-regulated sodium reabsorption	shock
path:04960	Aldosterone-regulated sodium reabsorption	paralysis
path:04960	Aldosterone-regulated sodium reabsorption	glycosuria
path:04960	Aldosterone-regulated sodium reabsorption	hepatic failure
path:04960	Aldosterone-regulated sodium reabsorption	hyperglycemia
path:04960	Aldosterone-regulated sodium reabsorption	albuminuria
path:04970	Salivary secretion	postural hypotension
path:04970	Salivary secretion	spasm
path:04970	Salivary secretion	weakness
path:04970	Salivary secretion	gynecomastia
path:04970	Salivary secretion	sore throat
path:04970	Salivary secretion	impotence
path:04970	Salivary secretion	increased salivation
path:04970	Salivary secretion	paralytic ileus
path:04970	Salivary secretion	palpitations
path:04970	Salivary secretion	priapism
path:04970	Salivary secretion	dry mouth
path:04970	Salivary secretion	Parkinson
path:04970	Salivary secretion	glycosuria
path:04970	Salivary secretion	bradycardia
path:04970	Salivary secretion	nightmares
path:04970	Salivary secretion	tardive dyskinesia
path:04970	Salivary secretion	lightheadedness
path:04970	Salivary secretion	nasal congestion
path:04970	Salivary secretion	urinary retention
path:04970	Salivary secretion	AV block
path:04970	Salivary secretion	mental depression
path:04970	Salivary secretion	memory loss
path:04970	Salivary secretion	heart block
path:04970	Salivary secretion	tachycardia
path:04970	Salivary secretion	sweating
path:04970	Salivary secretion	galactorrhea
path:04971	Gastric acid secretion	paralytic ileus
path:04971	Gastric acid secretion	Parkinson
path:04971	Gastric acid secretion	tardive dyskinesia
path:04971	Gastric acid secretion	gynecomastia
path:04971	Gastric acid secretion	nasal congestion
path:04972	Pancreatic secretion	postural hypotension
path:04972	Pancreatic secretion	nasal congestion
path:04974	Protein digestion and absorption	hyperuricemia
path:04974	Protein digestion and absorption	Stevens - Johnson syndrome
path:04974	Protein digestion and absorption	hypersensitivity
path:04974	Protein digestion and absorption	vasculitis
path:04974	Protein digestion and absorption	pancreatitis
path:04974	Protein digestion and absorption	jaundice
path:04974	Protein digestion and absorption	hemolytic anemia
path:05010	Alzheimer's disease	leukopenia
path:05010	Alzheimer's disease	hepatitis
path:05010	Alzheimer's disease	angioedema
path:05010	Alzheimer's disease	jaundice
path:05010	Alzheimer's disease	gynecomastia
path:05012	Parkinson's disease	diarrhea
path:05012	Parkinson's disease	tachycardia
path:05012	Parkinson's disease	agitation

path:05012	Parkinson's disease	impotence
path:05014	Amyotrophic lateral sclerosis (ALS)	anorexia
path:05014	Amyotrophic lateral sclerosis (ALS)	hepatic failure
path:05014	Amyotrophic lateral sclerosis (ALS)	stomatitis
path:05014	Amyotrophic lateral sclerosis (ALS)	hyperkalemia
path:05014	Amyotrophic lateral sclerosis (ALS)	acute renal failure
path:05014	Amyotrophic lateral sclerosis (ALS)	leukopenia
path:05014	Amyotrophic lateral sclerosis (ALS)	neuropathy
path:05014	Amyotrophic lateral sclerosis (ALS)	sepsis
path:05014	Amyotrophic lateral sclerosis (ALS)	nausea
path:05014	Amyotrophic lateral sclerosis (ALS)	dyspnea
path:05014	Amyotrophic lateral sclerosis (ALS)	constitutional symptoms
path:05014	Amyotrophic lateral sclerosis (ALS)	peripheral neuropathy
path:05014	Amyotrophic lateral sclerosis (ALS)	neutropenia
path:05014	Amyotrophic lateral sclerosis (ALS)	fever
path:05014	Amyotrophic lateral sclerosis (ALS)	hypotension
path:05014	Amyotrophic lateral sclerosis (ALS)	seizures
path:05016	Huntington's disease	dyspnea
path:05016	Huntington's disease	anemia
path:05016	Huntington's disease	myocardial infarction
path:05020	Prion diseases	epistaxis
path:05020	Prion diseases	anorexia
path:05020	Prion diseases	pancreatitis
path:05020	Prion diseases	alopecia
path:05020	Prion diseases	dyspnea
path:05020	Prion diseases	edema
path:05020	Prion diseases	hematuria
path:05020	Prion diseases	congestive heart failure
path:05020	Prion diseases	erythema multiforme
path:05020	Prion diseases	infection
path:05020	Prion diseases	hemorrhage
path:05020	Prion diseases	stomatitis
path:05020	Prion diseases	arthralgia
path:05020	Prion diseases	anemia
path:05020	Prion diseases	peripheral neuropathy
path:05020	Prion diseases	purpura
path:05020	Prion diseases	fever
path:05110	Vibrio cholerae infection	postural hypotension
path:05120	Epithelial cell signaling in Helicobacter pylori infection	hepatitis
path:05120	Epithelial cell signaling in Helicobacter pylori infection	thrombocytopenia
path:05120	Epithelial cell signaling in Helicobacter pylori infection	sepsis
path:05120	Epithelial cell signaling in Helicobacter pylori infection	pneumonia
path:05120	Epithelial cell signaling in Helicobacter pylori infection	epistaxis
path:05120	Epithelial cell signaling in Helicobacter pylori infection	cough
path:05120	Epithelial cell signaling in Helicobacter pylori infection	hematuria
path:05120	Epithelial cell signaling in Helicobacter pylori infection	dyspnea
path:05120	Epithelial cell signaling in Helicobacter pylori infection	alopecia
path:05120	Epithelial cell signaling in Helicobacter pylori infection	constitutional symptoms
path:05120	Epithelial cell signaling in Helicobacter pylori infection	anemia
path:05120	Epithelial cell signaling in Helicobacter pylori infection	toxic epidermal necrolysis
path:05120	Epithelial cell signaling in Helicobacter pylori infection	neuropathy
path:05120	Epithelial cell signaling in Helicobacter pylori infection	dermatitis
path:05120	Epithelial cell signaling in Helicobacter pylori infection	anaphylaxis
path:05120	Epithelial cell signaling in Helicobacter pylori infection	flushing
path:05120	Epithelial cell signaling in Helicobacter pylori infection	asthenia
path:05120	Epithelial cell signaling in Helicobacter pylori infection	neutropenia

path:05120	Epithelial cell signaling in Helicobacter pylori infection	conjunctivitis
path:05120	Epithelial cell signaling in Helicobacter pylori infection	diarrhea
path:05120	Epithelial cell signaling in Helicobacter pylori infection	leukopenia
path:05120	Epithelial cell signaling in Helicobacter pylori infection	abdominal pain
path:05120	Epithelial cell signaling in Helicobacter pylori infection	upper respiratory tract infection
path:05120	Epithelial cell signaling in Helicobacter pylori infection	hypokalemia
path:05120	Epithelial cell signaling in Helicobacter pylori infection	gastrointestinal hemorrhage
path:05120	Epithelial cell signaling in Helicobacter pylori infection	Dyspepsia
path:05120	Epithelial cell signaling in Helicobacter pylori infection	Pharyngitis
path:05120	Epithelial cell signaling in Helicobacter pylori infection	chills
path:05120	Epithelial cell signaling in Helicobacter pylori infection	pleural effusion
path:05120	Epithelial cell signaling in Helicobacter pylori infection	erythema multiforme
path:05120	Epithelial cell signaling in Helicobacter pylori infection	chest pain
path:05120	Epithelial cell signaling in Helicobacter pylori infection	malaise
path:05120	Epithelial cell signaling in Helicobacter pylori infection	peripheral neuropathy
path:05120	Epithelial cell signaling in Helicobacter pylori infection	congestive heart failure
path:05120	Epithelial cell signaling in Helicobacter pylori infection	infection
path:05120	Epithelial cell signaling in Helicobacter pylori infection	rash
path:05120	Epithelial cell signaling in Helicobacter pylori infection	myalgia
path:05120	Epithelial cell signaling in Helicobacter pylori infection	heart failure
path:05120	Epithelial cell signaling in Helicobacter pylori infection	pancreatitis
path:05120	Epithelial cell signaling in Helicobacter pylori infection	stomatitis
path:05120	Epithelial cell signaling in Helicobacter pylori infection	anorexia
path:05120	Epithelial cell signaling in Helicobacter pylori infection	hypersensitivity
path:05131	Shigellosis	abdominal pain
path:05131	Shigellosis	hematuria
path:05131	Shigellosis	vertigo
path:05131	Shigellosis	purpura
path:05131	Shigellosis	angioedema
path:05131	Shigellosis	Dyspepsia
path:05131	Shigellosis	anaphylaxis
path:05131	Shigellosis	conjunctivitis
path:05131	Shigellosis	anorexia
path:05131	Shigellosis	dyspnea
path:05131	Shigellosis	malaise
path:05131	Shigellosis	edema
path:05131	Shigellosis	hepatitis
path:05131	Shigellosis	fever
path:05131	Shigellosis	epistaxis
path:05131	Shigellosis	pancreatitis
path:05131	Shigellosis	alopecia
path:05131	Shigellosis	anemia
path:05131	Shigellosis	jaundice
path:05131	Shigellosis	flushing
path:05140	Leishmaniasis	rectal hemorrhage
path:05140	Leishmaniasis	sepsis
path:05140	Leishmaniasis	gastroenteritis
path:05140	Leishmaniasis	glossitis
path:05140	Leishmaniasis	toxic epidermal necrolysis
path:05140	Leishmaniasis	purpura
path:05140	Leishmaniasis	cystitis
path:05140	Leishmaniasis	lymphadenopathy
path:05140	Leishmaniasis	pruritus
path:05140	Leishmaniasis	melena
path:05140	Leishmaniasis	thrombocytopenia
path:05140	Leishmaniasis	hepatitis

path:05140	Leishmaniasis	anxiety
path:05140	Leishmaniasis	dyspnea
path:05140	Leishmaniasis	gastrointestinal hemorrhage
path:05140	Leishmaniasis	Dyspepsia
path:05140	Leishmaniasis	anemia
path:05140	Leishmaniasis	hemorrhage
path:05140	Leishmaniasis	malaise
path:05140	Leishmaniasis	anorexia
path:05140	Leishmaniasis	gastritis
path:05140	Leishmaniasis	aplastic anemia
path:05140	Leishmaniasis	renal failure
path:05140	Leishmaniasis	myocardial infarction
path:05140	Leishmaniasis	vasculitis
path:05140	Leishmaniasis	insomnia
path:05140	Leishmaniasis	eructation
path:05140	Leishmaniasis	edema
path:05140	Leishmaniasis	agranulocytosis
path:05140	Leishmaniasis	hepatic failure
path:05140	Leishmaniasis	hypersensitivity
path:05140	Leishmaniasis	Stevens - Johnson syndrome
path:05140	Leishmaniasis	pancreatitis
path:05140	Leishmaniasis	acute renal failure
path:05140	Leishmaniasis	erythema multiforme
path:05140	Leishmaniasis	photosensitivity
path:05140	Leishmaniasis	hematemesis
path:05140	Leishmaniasis	epistaxis
path:05140	Leishmaniasis	esophagitis
path:05140	Leishmaniasis	herpes simplex
path:05140	Leishmaniasis	heartburn
path:05140	Leishmaniasis	colitis
path:05140	Leishmaniasis	pancytopenia
path:05140	Leishmaniasis	hearing loss
path:05140	Leishmaniasis	abdominal pain
path:05140	Leishmaniasis	hyperkalemia
path:05140	Leishmaniasis	exfoliative dermatitis
path:05140	Leishmaniasis	hyperglycemia
path:05140	Leishmaniasis	stomatitis
path:05140	Leishmaniasis	cellulitis
path:05140	Leishmaniasis	peptic ulcer
path:05140	Leishmaniasis	infection
path:05140	Leishmaniasis	fever
path:05140	Leishmaniasis	tremor
path:05140	Leishmaniasis	vertigo
path:05140	Leishmaniasis	proteinuria
path:05140	Leishmaniasis	hemolytic anemia
path:05140	Leishmaniasis	dysuria
path:05140	Leishmaniasis	ecchymosis
path:05140	Leishmaniasis	hematuria
path:05140	Leishmaniasis	anaphylaxis
path:05140	Leishmaniasis	congestive heart failure
path:05140	Leishmaniasis	eczema
path:05140	Leishmaniasis	flatulence
path:05140	Leishmaniasis	asthma
path:05140	Leishmaniasis	angioedema
path:05140	Leishmaniasis	conjunctivitis
path:05140	Leishmaniasis	pneumonia

path:05140	Leishmaniasis	jaundice
path:05140	Leishmaniasis	interstitial nephritis
path:05140	Leishmaniasis	ulcer
path:05140	Leishmaniasis	eosinophilia
path:05140	Leishmaniasis	alopecia
path:05140	Leishmaniasis	palpitations
path:05140	Leishmaniasis	tinnitus
path:05142	Chagas disease	proteinuria
path:05142	Chagas disease	muscle cramps
path:05142	Chagas disease	bronchitis
path:05142	Chagas disease	hypoglycemia
path:05142	Chagas disease	cough
path:05142	Chagas disease	arrhythmia
path:05142	Chagas disease	hemolytic anemia
path:05142	Chagas disease	chest pain
path:05142	Chagas disease	arthritis
path:05142	Chagas disease	gout
path:05142	Chagas disease	hyponatremia
path:05142	Chagas disease	pancreatitis
path:05142	Chagas disease	palpitations
path:05142	Chagas disease	neutropenia
path:05142	Chagas disease	conjunctivitis
path:05142	Chagas disease	pneumonia
path:05142	Chagas disease	hyperkalemia
path:05142	Chagas disease	epistaxis
path:05142	Chagas disease	hypotension
path:05142	Chagas disease	Dyspepsia
path:05142	Chagas disease	acute renal failure
path:05142	Chagas disease	flushing
path:05142	Chagas disease	angina pectoris
path:05142	Chagas disease	abdominal pain
path:05142	Chagas disease	malaise
path:05142	Chagas disease	rhinitis
path:05142	Chagas disease	upper respiratory tract infection
path:05142	Chagas disease	Influenza
path:05142	Chagas disease	Sinusitis
path:05142	Chagas disease	anemia
path:05142	Chagas disease	hepatitis
path:05142	Chagas disease	dyspnea
path:05142	Chagas disease	gastroenteritis
path:05142	Chagas disease	toxic epidermal necrolysis
path:05144	Malaria	abdominal pain
path:05144	Malaria	peripheral neuropathy
path:05144	Malaria	ulcer
path:05144	Malaria	hypersensitivity
path:05144	Malaria	flatulence
path:05144	Malaria	eczema
path:05144	Malaria	infection
path:05144	Malaria	purpura
path:05144	Malaria	Dyspepsia
path:05145	Toxoplasmosis	pleural effusion
path:05145	Toxoplasmosis	atrial fibrillation
path:05145	Toxoplasmosis	toxic epidermal necrolysis
path:05145	Toxoplasmosis	esophagitis
path:05145	Toxoplasmosis	neutropenia
path:05145	Toxoplasmosis	dehydration

path:05145	Toxoplasmosis	jaundice
path:05145	Toxoplasmosis	epistaxis
path:05145	Toxoplasmosis	gastroenteritis
path:05145	Toxoplasmosis	conjunctivitis
path:05145	Toxoplasmosis	arthralgia
path:05145	Toxoplasmosis	sepsis
path:05145	Toxoplasmosis	leukopenia
path:05145	Toxoplasmosis	thrombocytopenia
path:05145	Toxoplasmosis	ecchymosis
path:05145	Toxoplasmosis	hematuria
path:05145	Toxoplasmosis	alopecia
path:05145	Toxoplasmosis	pneumonia
path:05145	Toxoplasmosis	herpes simplex
path:05145	Toxoplasmosis	abdominal pain
path:05145	Toxoplasmosis	hypersensitivity
path:05145	Toxoplasmosis	ulcer
path:05145	Toxoplasmosis	pancytopenia
path:05145	Toxoplasmosis	stomatitis
path:05145	Toxoplasmosis	gastritis
path:05145	Toxoplasmosis	urinary tract infection
path:05145	Toxoplasmosis	anorexia
path:05145	Toxoplasmosis	erythema
path:05145	Toxoplasmosis	gastrointestinal hemorrhage
path:05145	Toxoplasmosis	neuropathy
path:05145	Toxoplasmosis	congestive heart failure
path:05145	Toxoplasmosis	infection
path:05145	Toxoplasmosis	heart failure
path:05145	Toxoplasmosis	hypokalemia
path:05145	Toxoplasmosis	upper respiratory tract infection
path:05145	Toxoplasmosis	weight loss
path:05145	Toxoplasmosis	hepatitis
path:05145	Toxoplasmosis	pancreatitis
path:05145	Toxoplasmosis	dermatitis
path:05145	Toxoplasmosis	anemia
path:05145	Toxoplasmosis	pruritus
path:05145	Toxoplasmosis	fever
path:05145	Toxoplasmosis	peripheral neuropathy
path:05146	Amoebiasis	flushing
path:05146	Amoebiasis	upper respiratory tract infection
path:05146	Amoebiasis	hypokalemia
path:05146	Amoebiasis	pulmonary edema
path:05146	Amoebiasis	proteinuria
path:05160	Hepatitis C	arthritis
path:05160	Hepatitis C	neutropenia
path:05160	Hepatitis C	pancreatitis
path:05160	Hepatitis C	fever
path:05160	Hepatitis C	anorexia
path:05160	Hepatitis C	stomatitis
path:05160	Hepatitis C	abdominal pain
path:05160	Hepatitis C	dyspnea
path:05160	Hepatitis C	infection
path:05160	Hepatitis C	alopecia
path:05200	Pathways in cancer	erythema multiforme
path:05200	Pathways in cancer	pancreatitis
path:05200	Pathways in cancer	conjunctivitis
path:05200	Pathways in cancer	pleural effusion

path:05200	Pathways in cancer	hematemesis
path:05200	Pathways in cancer	interstitial nephritis
path:05200	Pathways in cancer	pneumonia
path:05200	Pathways in cancer	alopecia
path:05200	Pathways in cancer	weight loss
path:05200	Pathways in cancer	congestive heart failure
path:05200	Pathways in cancer	Stevens - Johnson syndrome
path:05200	Pathways in cancer	hepatic failure
path:05200	Pathways in cancer	interstitial pneumonitis
path:05200	Pathways in cancer	erythema
path:05200	Pathways in cancer	edema
path:05200	Pathways in cancer	renal failure
path:05200	Pathways in cancer	hypersensitivity
path:05200	Pathways in cancer	dyspnea
path:05200	Pathways in cancer	pulmonary edema
path:05200	Pathways in cancer	toxic epidermal necrolysis
path:05200	Pathways in cancer	anorexia
path:05200	Pathways in cancer	anemia
path:05200	Pathways in cancer	infection
path:05200	Pathways in cancer	fever
path:05200	Pathways in cancer	gastrointestinal hemorrhage
path:05200	Pathways in cancer	exfoliative dermatitis
path:05200	Pathways in cancer	Dyspepsia
path:05200	Pathways in cancer	hypokalemia
path:05200	Pathways in cancer	rectal hemorrhage
path:05200	Pathways in cancer	dry skin
path:05200	Pathways in cancer	peripheral edema
path:05200	Pathways in cancer	melena
path:05200	Pathways in cancer	cancer
path:05200	Pathways in cancer	ulcer
path:05200	Pathways in cancer	hearing loss
path:05200	Pathways in cancer	vasculitis
path:05200	Pathways in cancer	gastritis
path:05200	Pathways in cancer	asthenia
path:05200	Pathways in cancer	thrombocytopenia
path:05200	Pathways in cancer	constitutional symptoms
path:05200	Pathways in cancer	hematuria
path:05200	Pathways in cancer	leukopenia
path:05200	Pathways in cancer	stomatitis
path:05200	Pathways in cancer	peripheral neuropathy
path:05200	Pathways in cancer	neutropenia
path:05200	Pathways in cancer	SGOT increased
path:05200	Pathways in cancer	sepsis
path:05200	Pathways in cancer	proteinuria
path:05200	Pathways in cancer	hemorrhage
path:05200	Pathways in cancer	aplastic anemia
path:05210	Colorectal cancer	asthenia
path:05210	Colorectal cancer	infection
path:05210	Colorectal cancer	heart failure
path:05210	Colorectal cancer	peripheral neuropathy
path:05210	Colorectal cancer	stomatitis
path:05210	Colorectal cancer	toxic epidermal necrolysis
path:05210	Colorectal cancer	chills
path:05210	Colorectal cancer	fever
path:05210	Colorectal cancer	Stevens - Johnson syndrome
path:05210	Colorectal cancer	constipation

path:05210	Colorectal cancer	hemorrhage
path:05210	Colorectal cancer	dyspnea
path:05210	Colorectal cancer	erythema multiforme
path:05210	Colorectal cancer	leukopenia
path:05210	Colorectal cancer	weakness
path:05210	Colorectal cancer	anorexia
path:05210	Colorectal cancer	neuropathy
path:05210	Colorectal cancer	neutropenia
path:05210	Colorectal cancer	thrombocytopenia
path:05210	Colorectal cancer	cough
path:05210	Colorectal cancer	anemia
path:05210	Colorectal cancer	hypersensitivity
path:05210	Colorectal cancer	gastrointestinal hemorrhage
path:05210	Colorectal cancer	alopecia
path:05210	Colorectal cancer	renal failure
path:05210	Colorectal cancer	malaise
path:05210	Colorectal cancer	myalgia
path:05210	Colorectal cancer	congestive heart failure
path:05210	Colorectal cancer	constitutional symptoms
path:05211	Renal cell carcinoma	anemia
path:05211	Renal cell carcinoma	infection
path:05211	Renal cell carcinoma	Dyspepsia
path:05211	Renal cell carcinoma	hypertension
path:05211	Renal cell carcinoma	asthenia
path:05211	Renal cell carcinoma	leukopenia
path:05211	Renal cell carcinoma	erythema multiforme
path:05211	Renal cell carcinoma	arthralgia
path:05211	Renal cell carcinoma	pancreatitis
path:05211	Renal cell carcinoma	dyspnea
path:05211	Renal cell carcinoma	Sinusitis
path:05211	Renal cell carcinoma	alopecia
path:05212	Pancreatic cancer	asthenia
path:05212	Pancreatic cancer	weight loss
path:05212	Pancreatic cancer	stomatitis
path:05212	Pancreatic cancer	hemorrhage
path:05212	Pancreatic cancer	alopecia
path:05212	Pancreatic cancer	arthritis
path:05212	Pancreatic cancer	arthralgia
path:05212	Pancreatic cancer	anorexia
path:05212	Pancreatic cancer	anemia
path:05212	Pancreatic cancer	congestive heart failure
path:05212	Pancreatic cancer	malaise
path:05212	Pancreatic cancer	leukopenia
path:05212	Pancreatic cancer	cough
path:05212	Pancreatic cancer	constitutional symptoms
path:05212	Pancreatic cancer	pancreatitis
path:05212	Pancreatic cancer	myalgia
path:05212	Pancreatic cancer	edema
path:05212	Pancreatic cancer	dyspnea
path:05212	Pancreatic cancer	thrombocytopenia
path:05212	Pancreatic cancer	infection
path:05212	Pancreatic cancer	fatigue
path:05212	Pancreatic cancer	fever
path:05213	Endometrial cancer	leukopenia
path:05213	Endometrial cancer	Vascular Disorders
path:05213	Endometrial cancer	erythema

path:05213	Endometrial cancer	pancreatitis
path:05213	Endometrial cancer	weight loss
path:05213	Endometrial cancer	asthenia
path:05213	Endometrial cancer	cough
path:05213	Endometrial cancer	constitutional symptoms
path:05213	Endometrial cancer	stomatitis
path:05213	Endometrial cancer	pain
path:05213	Endometrial cancer	alopecia
path:05213	Endometrial cancer	anorexia
path:05213	Endometrial cancer	anemia
path:05213	Endometrial cancer	dermatitis
path:05213	Endometrial cancer	infection
path:05213	Endometrial cancer	dyspnea
path:05213	Endometrial cancer	neutropenia
path:05213	Endometrial cancer	neuropathy
path:05213	Endometrial cancer	hemorrhage
path:05213	Endometrial cancer	flushing
path:05213	Endometrial cancer	congestive heart failure
path:05213	Endometrial cancer	peripheral edema
path:05214	Glioma	neutropenia
path:05214	Glioma	stomatitis
path:05214	Glioma	leukopenia
path:05214	Glioma	erythema
path:05214	Glioma	gynecomastia
path:05214	Glioma	constitutional symptoms
path:05214	Glioma	anemia
path:05214	Glioma	peripheral neuropathy
path:05214	Glioma	lupus
path:05214	Glioma	congestive heart failure
path:05215	Prostate cancer	leukopenia
path:05215	Prostate cancer	anorexia
path:05215	Prostate cancer	cancer
path:05215	Prostate cancer	anemia
path:05215	Prostate cancer	edema
path:05215	Prostate cancer	neutropenia
path:05215	Prostate cancer	stomatitis
path:05215	Prostate cancer	dyspnea
path:05215	Prostate cancer	constitutional symptoms
path:05215	Prostate cancer	congestive heart failure
path:05215	Prostate cancer	pleural effusion
path:05216	Thyroid cancer	infection
path:05216	Thyroid cancer	hypersensitivity
path:05216	Thyroid cancer	stomatitis
path:05216	Thyroid cancer	edema
path:05216	Thyroid cancer	leukopenia
path:05216	Thyroid cancer	peripheral edema
path:05216	Thyroid cancer	anemia
path:05216	Thyroid cancer	neutropenia
path:05216	Thyroid cancer	erythema
path:05216	Thyroid cancer	hemorrhage
path:05216	Thyroid cancer	constitutional symptoms
path:05216	Thyroid cancer	dermatitis
path:05216	Thyroid cancer	pancreatitis
path:05216	Thyroid cancer	dyspnea
path:05216	Thyroid cancer	pain
path:05216	Thyroid cancer	asthenia

path:05216	Thyroid cancer	anorexia
path:05216	Thyroid cancer	congestive heart failure
path:05218	Melanoma	stomatitis
path:05218	Melanoma	leukopenia
path:05218	Melanoma	infection
path:05218	Melanoma	congestive heart failure
path:05218	Melanoma	dyspnea
path:05218	Melanoma	asthenia
path:05218	Melanoma	anemia
path:05218	Melanoma	pancreatitis
path:05218	Melanoma	neutropenia
path:05218	Melanoma	fever
path:05218	Melanoma	pain
path:05218	Melanoma	hemorrhage
path:05218	Melanoma	cough
path:05218	Melanoma	constitutional symptoms
path:05218	Melanoma	anorexia
path:05219	Bladder cancer	dyspnea
path:05219	Bladder cancer	vomiting
path:05219	Bladder cancer	dermatitis
path:05219	Bladder cancer	Dyspepsia
path:05219	Bladder cancer	stomatitis
path:05219	Bladder cancer	malaise
path:05219	Bladder cancer	anemia
path:05219	Bladder cancer	rash
path:05219	Bladder cancer	hemorrhage
path:05219	Bladder cancer	flushing
path:05219	Bladder cancer	myalgia
path:05219	Bladder cancer	constitutional symptoms
path:05219	Bladder cancer	erythema multiforme
path:05219	Bladder cancer	pleural effusion
path:05219	Bladder cancer	congestive heart failure
path:05219	Bladder cancer	fever
path:05219	Bladder cancer	Vascular Disorders
path:05219	Bladder cancer	abdominal pain
path:05219	Bladder cancer	pancreatitis
path:05219	Bladder cancer	anorexia
path:05219	Bladder cancer	asthenia
path:05219	Bladder cancer	hypersensitivity
path:05219	Bladder cancer	Stevens - Johnson syndrome
path:05219	Bladder cancer	viral infection
path:05219	Bladder cancer	infection
path:05219	Bladder cancer	peripheral neuropathy
path:05219	Bladder cancer	fatigue
path:05219	Bladder cancer	heart failure
path:05219	Bladder cancer	alopecia
path:05219	Bladder cancer	pulmonary edema
path:05219	Bladder cancer	neuropathy
path:05219	Bladder cancer	leukopenia
path:05219	Bladder cancer	toxic epidermal necrolysis
path:05219	Bladder cancer	epistaxis
path:05219	Bladder cancer	exfoliative dermatitis
path:05220	Chronic myeloid leukemia	asthenia
path:05220	Chronic myeloid leukemia	anorexia
path:05220	Chronic myeloid leukemia	anemia
path:05220	Chronic myeloid leukemia	dyspnea

path:05220	Chronic myeloid leukemia	fever
path:05220	Chronic myeloid leukemia	neutropenia
path:05220	Chronic myeloid leukemia	pancreatitis
path:05220	Chronic myeloid leukemia	stomatitis
path:05221	Acute myeloid leukemia	anemia
path:05221	Acute myeloid leukemia	dyspnea
path:05221	Acute myeloid leukemia	hemorrhage
path:05222	Small cell lung cancer	esophagitis
path:05222	Small cell lung cancer	exfoliative dermatitis
path:05222	Small cell lung cancer	hepatic failure
path:05222	Small cell lung cancer	erythema multiforme
path:05222	Small cell lung cancer	dysuria
path:05222	Small cell lung cancer	alopecia
path:05222	Small cell lung cancer	thrombocytopenia
path:05222	Small cell lung cancer	flatulence
path:05222	Small cell lung cancer	interstitial nephritis
path:05222	Small cell lung cancer	gastritis
path:05222	Small cell lung cancer	dyspnea
path:05222	Small cell lung cancer	anaphylaxis
path:05222	Small cell lung cancer	Stevens - Johnson syndrome
path:05222	Small cell lung cancer	hemorrhage
path:05222	Small cell lung cancer	eructation
path:05222	Small cell lung cancer	stomatitis
path:05222	Small cell lung cancer	sepsis
path:05222	Small cell lung cancer	tinnitus
path:05222	Small cell lung cancer	agranulocytosis
path:05222	Small cell lung cancer	ecchymosis
path:05222	Small cell lung cancer	asthenia
path:05222	Small cell lung cancer	pneumonia
path:05222	Small cell lung cancer	hematemesis
path:05222	Small cell lung cancer	hepatitis
path:05222	Small cell lung cancer	leukopenia
path:05222	Small cell lung cancer	coma
path:05222	Small cell lung cancer	nephrotic syndrome
path:05222	Small cell lung cancer	gastrointestinal hemorrhage
path:05222	Small cell lung cancer	gastroenteritis
path:05222	Small cell lung cancer	toxic epidermal necrolysis
path:05222	Small cell lung cancer	pancreatitis
path:05222	Small cell lung cancer	herpes simplex
path:05222	Small cell lung cancer	hemolytic anemia
path:05222	Small cell lung cancer	hypertension
path:05222	Small cell lung cancer	aplastic anemia
path:05222	Small cell lung cancer	proteinuria
path:05222	Small cell lung cancer	cystitis
path:05222	Small cell lung cancer	congestive heart failure
path:05222	Small cell lung cancer	ulcer
path:05222	Small cell lung cancer	renal failure
path:05222	Small cell lung cancer	hypercholesterolemia
path:05222	Small cell lung cancer	anemia
path:05222	Small cell lung cancer	hypersensitivity
path:05222	Small cell lung cancer	hematuria
path:05222	Small cell lung cancer	peptic ulcer
path:05222	Small cell lung cancer	hyperkalemia
path:05222	Small cell lung cancer	pulmonary edema
path:05222	Small cell lung cancer	lymphadenopathy
path:05222	Small cell lung cancer	angioedema

path:05222	Small cell lung cancer	Dyspepsia
path:05222	Small cell lung cancer	rectal hemorrhage
path:05222	Small cell lung cancer	pancytopenia
path:05222	Small cell lung cancer	colitis
path:05222	Small cell lung cancer	melena
path:05222	Small cell lung cancer	hearing loss
path:05222	Small cell lung cancer	vasculitis
path:05223	Non-small cell lung cancer	pleural effusion
path:05223	Non-small cell lung cancer	congestive heart failure
path:05223	Non-small cell lung cancer	stomatitis
path:05223	Non-small cell lung cancer	erythema
path:05223	Non-small cell lung cancer	anemia
path:05223	Non-small cell lung cancer	infection
path:05223	Non-small cell lung cancer	neutropenia
path:05223	Non-small cell lung cancer	dyspnea
path:05223	Non-small cell lung cancer	dermatitis
path:05223	Non-small cell lung cancer	hemorrhage
path:05223	Non-small cell lung cancer	constitutional symptoms
path:05223	Non-small cell lung cancer	neuropathy
path:05310	Asthma	Sinusitis
path:05310	Asthma	upper respiratory tract infection
path:05310	Asthma	Pharyngitis
path:05310	Asthma	fever
path:05310	Asthma	bronchitis
path:05320	Autoimmune thyroid disease	fever
path:05320	Autoimmune thyroid disease	edema
path:05322	Systemic lupus erythematosus	anorexia
path:05330	Allograft rejection	abdominal pain
path:05330	Allograft rejection	bronchitis
path:05330	Allograft rejection	hypersensitivity
path:05330	Allograft rejection	fever
path:05330	Allograft rejection	Dyspepsia
path:05330	Allograft rejection	conjunctivitis
path:05330	Allograft rejection	Pharyngitis
path:05330	Allograft rejection	infection
path:05330	Allograft rejection	epistaxis
path:05330	Allograft rejection	Sinusitis
path:05330	Allograft rejection	upper respiratory tract infection
path:05332	Graft-versus-host disease	infection
path:05332	Graft-versus-host disease	hemorrhage
path:05332	Graft-versus-host disease	malaise
path:05332	Graft-versus-host disease	fever
path:05332	Graft-versus-host disease	anemia
path:05332	Graft-versus-host disease	anorexia
path:05410	Hypertrophic cardiomyopathy (HCM)	hemolytic anemia
path:05410	Hypertrophic cardiomyopathy (HCM)	flushing
path:05410	Hypertrophic cardiomyopathy (HCM)	cerebrovascular accident
path:05410	Hypertrophic cardiomyopathy (HCM)	hyponatremia
path:05410	Hypertrophic cardiomyopathy (HCM)	muscle cramps
path:05410	Hypertrophic cardiomyopathy (HCM)	hepatitis
path:05410	Hypertrophic cardiomyopathy (HCM)	Stevens - Johnson syndrome
path:05412	Arrhythmogenic right ventricular cardiomyopathy (ARVC)	gastrointestinal hemorrhage
path:05414	Dilated cardiomyopathy	mental depression
path:05414	Dilated cardiomyopathy	shortness of breath
path:05414	Dilated cardiomyopathy	AV block
path:05414	Dilated cardiomyopathy	postural hypotension

path:05414	Dilated cardiomyopathy	muscle cramps
path:05414	Dilated cardiomyopathy	bradycardia
path:05414	Dilated cardiomyopathy	nasal congestion
path:05416	Viral myocarditis	leukopenia
path:05416	Viral myocarditis	flushing
path:05416	Viral myocarditis	cough
path:05416	Viral myocarditis	infection
path:05416	Viral myocarditis	anemia
path:05416	Viral myocarditis	stomatitis
path:05416	Viral myocarditis	dyspnea
path:05416	Viral myocarditis	headache
path:05416	Viral myocarditis	anorexia
path:05416	Viral myocarditis	myalgia
path:05416	Viral myocarditis	dermatitis
path:05416	Viral myocarditis	hematuria
path:05416	Viral myocarditis	hypersensitivity

Dataset S2. The ADR-ADR network

ADR	ADR
abdominal pain	epistaxis
abdominal pain	conjunctivitis
abdominal pain	upper respiratory tract infection
abdominal pain	fever
abdominal pain	pancreatitis
abdominal pain	hypersensitivity
abdominal pain	malaise
abdominal pain	purpura
abdominal pain	hepatitis
abdominal pain	infection
abdominal pain	muscle cramps
abdominal pain	alopecia
abdominal pain	angioedema
abdominal pain	arthritis
abdominal pain	gastroenteritis
abdominal pain	rhinitis
abdominal pain	anorexia
abdominal pain	myalgia
abdominal pain	pneumonia
abdominal pain	eczema
abdominal pain	dermatitis
abdominal pain	toxic epidermal necrolysis
abnormal gait	dysmenorrhea
abnormal gait	goiter
abnormal gait	personality disorder
abnormal gait	grand mal
abnormal gait	paranoia
abnormal gait	ventricular tachycardia
abnormal gait	anxiety
abnormal gait	psoriasis
abnormal gait	seizures
abnormal gait	spasm
abnormal gait	sweating
abnormal gait	delirium
abnormal gait	heart block
abnormal gait	torticollis
abnormal gait	dry mouth
abnormal gait	galactorrhea
abnormal gait	hallucinations
abnormal gait	nightmares
abnormal gait	priapism
abnormal gait	syncope
abnormal gait	urinary incontinence
abnormal gait	confusion
abnormal gait	increased salivation
abnormal gait	somnolence
abnormal gait	tardive dyskinesia
abnormal gait	urinary retention
abnormal gait	ataxia
abnormal gait	bradycardia
abnormal gait	dyskinesia
abnormal gait	hypotension

abnormal gait	insomnia
abnormal gait	palpitations
abnormal gait	paralytic ileus
abnormal gait	impotence
abnormal gait	nasal congestion
abnormal vision	blurred vision
abnormal vision	keratitis
abnormal vision	serotonin syndrome
abnormal vision	supraventricular extrasystoles
abnormal vision	agitation
abnormal vision	grand mal
abnormal vision	paranoia
abnormal vision	photophobia
abnormal vision	ventricular tachycardia
abnormal vision	nervousness
abnormal vision	psoriasis
abnormal vision	sweating
abnormal vision	heart block
abnormal vision	torticollis
abnormal vision	dry mouth
abnormal vision	galactorrhea
abnormal vision	nightmares
abnormal vision	priapism
abnormal vision	syncope
abnormal vision	increased salivation
abnormal vision	somnolence
abnormal vision	tardive dyskinesia
abnormal vision	urinary retention
abnormal vision	bradycardia
abnormal vision	dyskinesia
abnormal vision	hypotension
abnormal vision	insomnia
abnormal vision	palpitations
abnormal vision	paralytic ileus
abnormal vision	impotence
abnormal vision	nasal congestion
abuse	drug dependence
abuse	fecal impaction
abuse	glaucoma
abuse	hyperacusis
abuse	hypothermia
abuse	hypoventilation
abuse	labyrinthitis
abuse	pyelonephritis
abuse	sneezing
abuse	stridor
abuse	urinary hesitancy
abuse	ventricular extrasystoles
abuse	voice alteration
abuse	wheezing
acidosis	amblyopia
acidosis	arthrosis
acidosis	carcinoma
acidosis	contact dermatitis

acidosis	hemorrhoids
acidosis	neck rigidity
acidosis	polyuria
acidosis	tenesmus
acidosis	vaginal hemorrhage
acidosis	vaginitis
acute renal failure	hyperkalemia
acute renal failure	pruritus
agitation	blurred vision
agitation	keratitis
agitation	serotonin syndrome
agitation	supraventricular extrasystoles
agitation	impotence
agitation	tachycardia
agitation	grand mal
agitation	paranoia
agitation	photophobia
agitation	ventricular tachycardia
agitation	nervousness
agitation	psoriasis
agitation	sweating
agitation	heart block
agitation	torticollis
agitation	dry mouth
agitation	galactorrhea
agitation	nightmares
agitation	priapism
agitation	syncope
agranulocytosis	asthma
agranulocytosis	cystitis
agranulocytosis	peptic ulcer
agranulocytosis	exfoliative dermatitis
agranulocytosis	aplastic anemia
agranulocytosis	hearing loss
agranulocytosis	melena
agranulocytosis	colitis
agranulocytosis	ecchymosis
agranulocytosis	hematemesis
agranulocytosis	interstitial nephritis
agranulocytosis	rectal hemorrhage
agranulocytosis	dysuria
agranulocytosis	hepatic failure
agranulocytosis	herpes simplex
agranulocytosis	proteinuria
agranulocytosis	heartburn
agranulocytosis	eructation
agranulocytosis	hemolytic anemia
agranulocytosis	gastritis
agranulocytosis	congestive heart failure
agranulocytosis	pancytopenia
agranulocytosis	sepsis
agranulocytosis	hypertension
agranulocytosis	hematuria
agranulocytosis	toxic epidermal necrolysis

agranulocytosis	anaphylaxis
agranulocytosis	anxiety
agranulocytosis	esophagitis
agranulocytosis	lymphadenopathy
agranulocytosis	angioedema
agranulocytosis	gastrointestinal hemorrhage
albuminuria	glossitis
albuminuria	diplopia
albuminuria	herpes simplex
albuminuria	otitis media
albuminuria	phlebitis
albuminuria	eructation
albuminuria	amnesia
albuminuria	hiccup
albuminuria	breast pain
albuminuria	hypoxia
albuminuria	gastrointestinal hemorrhage
albuminuria	colitis
albuminuria	hallucinations
albuminuria	hyperglycemia
albuminuria	laryngitis
albuminuria	shock
albuminuria	urinary incontinence
albuminuria	coma
albuminuria	confusion
albuminuria	dysuria
albuminuria	urinary frequency
albuminuria	liver function tests abnormal
albuminuria	migraine
albuminuria	ulcerative stomatitis
albuminuria	ataxia
albuminuria	hypotension
albuminuria	gastritis
albuminuria	paralysis
albuminuria	pulmonary embolism
albuminuria	sleep disorder
albuminuria	ventricular fibrillation
albuminuria	tachycardia
alkaline phosphatase increased	blepharitis
alkaline phosphatase increased	dysphagia
alkaline phosphatase increased	amenorrhea
alkaline phosphatase increased	breast enlargement
alkaline phosphatase increased	delirium
alkaline phosphatase increased	torticollis
alkaline phosphatase increased	galactorrhea
alkaline phosphatase increased	priapism
alkaline phosphatase increased	increased salivation
alkaline phosphatase increased	tardive dyskinesia
alkaline phosphatase increased	urinary retention
alkaline phosphatase increased	constipation
alkaline phosphatase increased	dyskinesia
alkaline phosphatase increased	paralytic ileus
alkaline phosphatase increased	weight loss
alopecia	infection

alopecia	thrombocytopenia
alopecia	anemia
alopecia	dyspnea
alopecia	pancreatitis
alopecia	stomatitis
alopecia	toxic epidermal necrolysis
alopecia	leukopenia
alopecia	hematuria
alopecia	erythema multiforme
alopecia	hypersensitivity
alopecia	asthenia
alopecia	anorexia
alopecia	malaise
alopecia	congestive heart failure
alopecia	peripheral neuropathy
alopecia	pneumonia
alopecia	vertigo
alopecia	ulcer
alopecia	myalgia
alopecia	fever
alopecia	epistaxis
alopecia	dermatitis
alopecia	conjunctivitis
alopecia	ecchymosis
alopecia	hematemesis
alopecia	interstitial nephritis
alopecia	photosensitivity
alopecia	rectal hemorrhage
alopecia	anaphylaxis
alopecia	esophagitis
alopecia	angioedema
alopecia	gastrointestinal hemorrhage
alopecia	cough
alopecia	gastritis
alopecia	renal failure
alopecia	neutropenia
alopecia	arthralgia
alopecia	flatulence
alopecia	heart failure
alopecia	vasculitis
alopecia	hemorrhage
alopecia	sepsis
alopecia	hepatitis
alopecia	exfoliative dermatitis
alopecia	aplastic anemia
alopecia	hearing loss
alopecia	melena
alopecia	tinnitus
alopecia	vomiting
amblyopia	arthrosis
amblyopia	carcinoma
amblyopia	contact dermatitis
amblyopia	hemorrhoids
amblyopia	neck rigidity

amblyopia	polyuria
amblyopia	tenesmus
amblyopia	vaginal hemorrhage
amblyopia	vaginitis
amenorrhea	torticollis
amenorrhea	priapism
amenorrhea	increased salivation
amenorrhea	tardive dyskinesia
amenorrhea	dyskinesia
amenorrhea	paralytic ileus
amenorrhea	blepharitis
amenorrhea	dysphagia
amenorrhea	tachycardia
amenorrhea	breast enlargement
amenorrhea	gynecomastia
amenorrhea	spasm
amenorrhea	delirium
amenorrhea	dry mouth
amenorrhea	galactorrhea
amenorrhea	glycosuria
amnesia	ataxia
amnesia	otitis media
amnesia	phlebitis
amnesia	hiccup
amnesia	tremor
amnesia	breast pain
amnesia	glossitis
amnesia	hypoxia
amnesia	diplopia
amnesia	hallucinations
amnesia	laryngitis
amnesia	shock
amnesia	urinary incontinence
amnesia	coma
amnesia	confusion
amnesia	herpes simplex
amnesia	urinary frequency
amnesia	liver function tests abnormal
amnesia	migraine
amnesia	eructation
amnesia	hypotension
amnesia	apnea
amnesia	paralysis
amnesia	pulmonary embolism
amnesia	sleep disorder
amnesia	ventricular fibrillation
amnesia	tachycardia
amnesia	dry skin
amnesia	dysarthria
amnesia	lymphadenopathy
amnesia	nervousness
amnesia	neurosis
amnesia	gastrointestinal hemorrhage
anaphylaxis	hematuria

anaphylaxis	pneumonia
anaphylaxis	hepatitis
anaphylaxis	sepsis
anaphylaxis	malaise
anaphylaxis	pancreatitis
anaphylaxis	toxic epidermal necrolysis
anaphylaxis	cystitis
anaphylaxis	esophagitis
anaphylaxis	lymphadenopathy
anaphylaxis	peptic ulcer
anaphylaxis	angioedema
anaphylaxis	gastrointestinal hemorrhage
anemia	stomatitis
anemia	leukopenia
anemia	dyspnea
anemia	congestive heart failure
anemia	anorexia
anemia	asthenia
anemia	constitutional symptoms
anemia	infection
anemia	neutropenia
anemia	hemorrhage
anemia	thrombocytopenia
anemia	toxic epidermal necrolysis
anemia	pancreatitis
anemia	erythema multiforme
anemia	hematuria
anemia	malaise
anemia	dermatitis
anemia	peripheral neuropathy
anemia	gastrointestinal hemorrhage
anemia	hypersensitivity
anemia	cough
anemia	gastritis
anemia	neuropathy
anemia	pneumonia
anemia	renal failure
anemia	erythema
anemia	epistaxis
anemia	pleural effusion
angina pectoris	gout
angina pectoris	rhinitis
angina pectoris	hyponatremia
angina pectoris	chest pain
angina pectoris	hyperkalemia
angina pectoris	hemolytic anemia
angina pectoris	palpitations
angina pectoris	muscle cramps
angioedema	hepatitis
angioedema	vertigo
angioedema	purpura
angioedema	jaundice
angioedema	colitis
angioedema	ecchymosis

angioedema	hematemesis
angioedema	interstitial nephritis
angioedema	photosensitivity
angioedema	rectal hemorrhage
angioedema	exfoliative dermatitis
angioedema	cystitis
angioedema	peptic ulcer
angioedema	pancreatitis
angioedema	flatulence
angioedema	eructation
angioedema	hemolytic anemia
angioedema	aplastic anemia
angioedema	hearing loss
angioedema	melena
angioedema	tinnitus
angioedema	gastritis
angioedema	toxic epidermal necrolysis
angioedema	epistaxis
angioedema	dysuria
angioedema	hepatic failure
angioedema	herpes simplex
angioedema	proteinuria
angioedema	vasculitis
angioedema	hematuria
angioedema	esophagitis
angioedema	lymphadenopathy
angioedema	conjunctivitis
angioedema	erythema multiforme
anorexia	fever
anorexia	stomatitis
anorexia	infection
anorexia	dyspnea
anorexia	hypersensitivity
anorexia	neutropenia
anorexia	asthenia
anorexia	constitutional symptoms
anorexia	leukopenia
anorexia	malaise
anorexia	pancreatitis
anorexia	congestive heart failure
anorexia	edema
anorexia	hematuria
anorexia	cough
anorexia	neuropathy
anorexia	conjunctivitis
anorexia	hemorrhage
anorexia	thrombocytopenia
anorexia	dermatitis
anxiety	insomnia
anxiety	psoriasis
anxiety	heart block
anxiety	nightmares
anxiety	decreased libido
anxiety	dysmenorrhea

anxiety	goiter
anxiety	personality disorder
anxiety	bradycardia
anxiety	palpitations
anxiety	asthma
anxiety	bronchospasm
anxiety	dizziness
anxiety	grand mal
anxiety	lightheadedness
anxiety	paranoia
anxiety	sore throat
anxiety	ventricular tachycardia
anxiety	tremor
anxiety	mental depression
anxiety	seizures
anxiety	spasm
anxiety	sweating
aplastic anemia	hearing loss
aplastic anemia	melena
aplastic anemia	hematemesis
aplastic anemia	interstitial nephritis
aplastic anemia	rectal hemorrhage
aplastic anemia	hepatic failure
aplastic anemia	proteinuria
aplastic anemia	peptic ulcer
aplastic anemia	gastritis
aplastic anemia	sepsis
aplastic anemia	hematuria
aplastic anemia	exfoliative dermatitis
aplastic anemia	toxic epidermal necrolysis
aplastic anemia	colitis
aplastic anemia	ecchymosis
aplastic anemia	gastrointestinal hemorrhage
aplastic anemia	dysuria
aplastic anemia	ulcer
aplastic anemia	vasculitis
aplastic anemia	erythema multiforme
aplastic anemia	eructation
aplastic anemia	hemolytic anemia
aplastic anemia	pneumonia
aplastic anemia	renal failure
aplastic anemia	cystitis
aplastic anemia	esophagitis
aplastic anemia	pancytopenia
aplastic anemia	congestive heart failure
aplastic anemia	pancreatitis
aplastic anemia	flatulence
aplastic anemia	herpes simplex
aplastic anemia	heartburn
aplastic anemia	stomatitis
aplastic anemia	thrombocytopenia
aplastic anemia	asthma
aplastic anemia	hepatitis
apnea	nervousness

apnea	syncope
apnea	urinary retention
apnea	ataxia
apnea	dyskinesia
apnea	insomnia
apnea	impotence
apnea	tremor
apnea	cardiac arrest
apnea	paralysis
apnea	photophobia
apnea	dysarthria
apnea	hiccup
apnea	diplopia
apnea	galactorrhea
apnea	hallucinations
apnea	laryngitis
apnea	nightmares
apnea	shock
apnea	urinary incontinence
arrhythmia	hypoglycemia
arrhythmia	hypotension
arrhythmia	palpitations
arthralgia	infection
arthritis	pancreatitis
arthritis	rhinitis
arthritis	muscle cramps
arthritis	malaise
arthrosis	carcinoma
arthrosis	contact dermatitis
arthrosis	hemorrhoids
arthrosis	neck rigidity
arthrosis	polyuria
arthrosis	tenesmus
arthrosis	vaginal hemorrhage
arthrosis	vaginitis
asthenia	dyspnea
asthenia	leukopenia
asthenia	pancreatitis
asthenia	congestive heart failure
asthenia	stomatitis
asthenia	constitutional symptoms
asthenia	erythema multiforme
asthenia	hemorrhage
asthenia	renal failure
asthenia	rash
asthenia	infection
asthenia	thrombocytopenia
asthenia	cough
asthenia	neuropathy
asthenia	toxic epidermal necrolysis
asthenia	diarrhea
asthenia	vasculitis
asthenia	neutropenia
asthma	heartburn

asthma	exfoliative dermatitis
asthma	cystitis
asthma	peptic ulcer
asthma	glossitis
asthma	hearing loss
asthma	melena
asthma	colitis
asthma	ecchymosis
asthma	eczema
asthma	hematemesis
asthma	interstitial nephritis
asthma	rectal hemorrhage
ataxia	tremor
ataxia	hallucinations
ataxia	urinary incontinence
ataxia	confusion
ataxia	diplopia
ataxia	laryngitis
ataxia	tachycardia
ataxia	coma
ataxia	somnolence
ataxia	hiccup
ataxia	hypotension
ataxia	breast pain
ataxia	delirium
ataxia	hypoxia
ataxia	syncope
ataxia	otitis media
ataxia	phlebitis
ataxia	sleep disorder
ataxia	ventricular fibrillation
ataxia	urinary frequency
ataxia	urinary retention
ataxia	bradycardia
ataxia	dyskinesia
ataxia	eructation
ataxia	insomnia
ataxia	dysarthria
ataxia	nervousness
ataxia	neurosis
ataxia	impotence
ataxia	glossitis
ataxia	dysmenorrhea
ataxia	goiter
ataxia	hypothyroidism
ataxia	liver function tests abnormal
ataxia	menorrhagia
ataxia	migraine
ataxia	personality disorder
ataxia	psychosis
ataxia	galactorrhea
ataxia	nightmares
ataxia	shock
atrial fibrillation	dehydration

AV block	bradycardia
AV block	postural hypotension
AV block	mental depression
AV block	heart block
AV block	impotence
AV block	fatigue
AV block	nightmares
AV block	syncope
AV block	bronchospasm
AV block	dizziness
AV block	lightheadedness
AV block	sore throat
AV block	urinary retention
AV block	insomnia
AV block	palpitations
AV block	psoriasis
AV block	sweating
AV block	weakness
AV block	nasal congestion
AV block	dry mouth
AV block	galactorrhea
AV block	priapism
AV block	cold extremities
AV block	decreased libido
AV block	memory loss
AV block	psychosis
AV block	shortness of breath
AV block	weight gain
AV block	increased salivation
AV block	somnolence
AV block	tardive dyskinesia
blepharitis	dysphagia
blepharitis	breast enlargement
blepharitis	delirium
blepharitis	torticollis
blepharitis	galactorrhea
blepharitis	priapism
blepharitis	increased salivation
blepharitis	tardive dyskinesia
blepharitis	urinary retention
blepharitis	constipation
blepharitis	dyskinesia
blepharitis	paralytic ileus
blepharitis	weight loss
blurred vision	keratitis
blurred vision	serotonin syndrome
blurred vision	supraventricular extrasystoles
blurred vision	grand mal
blurred vision	paranoia
blurred vision	photophobia
blurred vision	ventricular tachycardia
blurred vision	nervousness
blurred vision	psoriasis
blurred vision	sweating

blurred vision	heart block
blurred vision	torticollis
blurred vision	dry mouth
blurred vision	galactorrhea
blurred vision	nightmares
blurred vision	priapism
blurred vision	syncope
blurred vision	increased salivation
blurred vision	somnolence
blurred vision	tardive dyskinesia
blurred vision	urinary retention
blurred vision	bradycardia
blurred vision	dyskinesia
blurred vision	hypotension
blurred vision	insomnia
blurred vision	palpitations
blurred vision	paralytic ileus
blurred vision	impotence
blurred vision	nasal congestion
bradycardia	heart block
bradycardia	nightmares
bradycardia	urinary retention
bradycardia	mental depression
bradycardia	psoriasis
bradycardia	sweating
bradycardia	postural hypotension
bradycardia	insomnia
bradycardia	impotence
bradycardia	nasal congestion
bradycardia	dry mouth
bradycardia	galactorrhea
bradycardia	priapism
bradycardia	syncope
bradycardia	tachycardia
bradycardia	grand mal
bradycardia	lightheadedness
bradycardia	paranoia
bradycardia	photophobia
bradycardia	sore throat
bradycardia	ventricular tachycardia
bradycardia	tremor
bradycardia	increased salivation
bradycardia	somnolence
bradycardia	tardive dyskinesia
bradycardia	dyskinesia
bradycardia	hypotension
bradycardia	palpitations
bradycardia	paralytic ileus
bradycardia	nervousness
bradycardia	spasm
bradycardia	delirium
bradycardia	torticollis
bradycardia	cold extremities
bradycardia	decreased libido

bradycardia	dysmenorrhea
bradycardia	goiter
bradycardia	keratitis
bradycardia	memory loss
bradycardia	personality disorder
bradycardia	psychosis
bradycardia	serotonin syndrome
bradycardia	shortness of breath
bradycardia	supraventricular extrasystoles
bradycardia	weight gain
bradycardia	fatigue
bradycardia	hallucinations
bradycardia	urinary incontinence
breast enlargement	delirium
breast enlargement	dysphagia
breast enlargement	tachycardia
breast enlargement	torticollis
breast enlargement	galactorrhea
breast enlargement	hallucinations
breast enlargement	priapism
breast enlargement	urinary incontinence
breast pain	laryngitis
breast pain	urinary frequency
breast pain	hypoxia
breast pain	hallucinations
breast pain	urinary incontinence
breast pain	coma
breast pain	confusion
breast pain	otitis media
breast pain	phlebitis
breast pain	pulmonary embolism
breast pain	sleep disorder
breast pain	eructation
breast pain	hiccup
breast pain	tachycardia
breast pain	tremor
breast pain	glossitis
breast pain	diplopia
breast pain	herpes simplex
breast pain	liver function tests abnormal
breast pain	migraine
breast pain	thrombophlebitis
breast pain	hypotension
breast pain	ventricular fibrillation
bronchitis	upper respiratory tract infection
bronchospasm	dizziness
bronchospasm	fatigue
bronchospasm	decreased libido
bronchospasm	lightheadedness
bronchospasm	sore throat
bronchospasm	mental depression
bronchospasm	psoriasis
bronchospasm	weakness
bronchospasm	flushing

bronchospasm	heart block
bronchospasm	nightmares
bronchospasm	syncope
bundle branch block	cardiomyopathy
bundle branch block	cholecystitis
bundle branch block	deafness
bundle branch block	fecal incontinence
bundle branch block	hyperhidrosis
bundle branch block	ischemic colitis
bundle branch block	leukocytosis
bundle branch block	neuralgia
bundle branch block	skin ulcer
bundle branch block	suicide attempt
cancer	pleural effusion
cancer	constitutional symptoms
cancer	pulmonary edema
carcinoma	contact dermatitis
carcinoma	hemorrhoids
carcinoma	neck rigidity
carcinoma	polyuria
carcinoma	tenesmus
carcinoma	vaginal hemorrhage
carcinoma	vaginitis
cardiac arrest	photophobia
cardiac arrest	nervousness
cardiac arrest	syncope
cardiomyopathy	cholecystitis
cardiomyopathy	deafness
cardiomyopathy	fecal incontinence
cardiomyopathy	hyperhidrosis
cardiomyopathy	ischemic colitis
cardiomyopathy	leukocytosis
cardiomyopathy	neuralgia
cardiomyopathy	skin ulcer
cardiomyopathy	suicide attempt
chest pain	gout
chest pain	palpitations
chest pain	rhinitis
chest pain	impotence
chills	stomatitis
chills	peripheral neuropathy
chills	neuropathy
chills	malaise
cholecystitis	deafness
cholecystitis	fecal incontinence
cholecystitis	hyperhidrosis
cholecystitis	ischemic colitis
cholecystitis	leukocytosis
cholecystitis	neuralgia
cholecystitis	skin ulcer
cholecystitis	suicide attempt
choreoathetosis	lactic dehydrogenase increased
choreoathetosis	pyuria
cold extremities	weight gain

cold extremities	grand mal
cold extremities	paranoia
cold extremities	ventricular tachycardia
cold extremities	neurosis
cold extremities	psoriasis
cold extremities	sweating
cold extremities	heart block
cold extremities	torticollis
cold extremities	dry mouth
cold extremities	eczema
cold extremities	fatigue
cold extremities	galactorrhea
cold extremities	nightmares
cold extremities	priapism
cold extremities	syncope
cold extremities	increased salivation
cold extremities	somnolence
cold extremities	tardive dyskinesia
cold extremities	urinary retention
cold extremities	constipation
cold extremities	dyskinesia
cold extremities	hypotension
cold extremities	insomnia
cold extremities	palpitations
cold extremities	paralytic ileus
cold extremities	impotence
cold extremities	nasal congestion
colitis	eructation
colitis	gastritis
colitis	ecchymosis
colitis	hematemesis
colitis	interstitial nephritis
colitis	rectal hemorrhage
colitis	dysuria
colitis	herpes simplex
colitis	gastrointestinal hemorrhage
colitis	cystitis
colitis	lymphadenopathy
colitis	peptic ulcer
colitis	glossitis
colitis	hearing loss
colitis	melena
colitis	hepatitis
colitis	exfoliative dermatitis
colitis	toxic epidermal necrolysis
colitis	flatulence
colitis	hepatic failure
colitis	proteinuria
colitis	hemolytic anemia
colitis	erythema multiforme
colitis	dry skin
colitis	esophagitis
colitis	stomatitis
colitis	pancytopenia

colitis	sepsis
colitis	hematuria
colitis	tinnitus
colitis	jaundice
colitis	photosensitivity
colitis	heartburn
colitis	liver function tests abnormal
colitis	migraine
colitis	ulcerative stomatitis
colitis	coma
colitis	ulcer
colitis	vasculitis
colitis	pancreatitis
colitis	otitis media
colitis	phlebitis
colitis	pulmonary embolism
colitis	pneumonia
colitis	renal failure
coma	hypoxia
coma	tremor
coma	confusion
coma	eructation
coma	diplopia
coma	hallucinations
coma	laryngitis
coma	urinary incontinence
coma	otitis media
coma	phlebitis
coma	sleep disorder
coma	dysuria
coma	herpes simplex
coma	urinary frequency
coma	dysarthria
coma	hiccup
coma	lymphadenopathy
coma	glossitis
coma	tinnitus
coma	tachycardia
coma	liver function tests abnormal
coma	migraine
coma	gastrointestinal hemorrhage
confusion	hallucinations
confusion	urinary incontinence
confusion	tremor
confusion	hypoxia
confusion	laryngitis
confusion	tachycardia
confusion	dysarthria
confusion	hiccup
confusion	hypotension
confusion	delirium
confusion	diplopia
confusion	otitis media
confusion	phlebitis

confusion	sleep disorder
confusion	ventricular fibrillation
confusion	somnolence
confusion	urinary frequency
confusion	eructation
confusion	neurosis
confusion	glossitis
confusion	galactorrhea
confusion	nightmares
confusion	syncope
confusion	dysmenorrhea
confusion	goiter
confusion	hypothyroidism
confusion	liver function tests abnormal
confusion	menorrhagia
confusion	migraine
confusion	personality disorder
confusion	dysuria
confusion	herpes simplex
confusion	increased salivation
confusion	urinary retention
congestive heart failure	stomatitis
congestive heart failure	constitutional symptoms
congestive heart failure	leukopenia
congestive heart failure	hemorrhage
congestive heart failure	dyspnea
congestive heart failure	toxic epidermal necrolysis
congestive heart failure	heart failure
congestive heart failure	hematuria
congestive heart failure	neuropathy
congestive heart failure	erythema multiforme
congestive heart failure	erythema
congestive heart failure	sepsis
congestive heart failure	pleural effusion
congestive heart failure	weight loss
congestive heart failure	thrombocytopenia
congestive heart failure	pancreatitis
congestive heart failure	neutropenia
congestive heart failure	infection
congestive heart failure	gastrointestinal hemorrhage
congestive heart failure	gastritis
congestive heart failure	pneumonia
congestive heart failure	renal failure
congestive heart failure	hearing loss
congestive heart failure	melena
congestive heart failure	peripheral edema
congestive heart failure	peripheral neuropathy
congestive heart failure	exfoliative dermatitis
congestive heart failure	ecchymosis
congestive heart failure	hematemesis
congestive heart failure	interstitial nephritis
congestive heart failure	pulmonary edema
congestive heart failure	rectal hemorrhage
congestive heart failure	esophagitis

congestive heart failure	peptic ulcer
congestive heart failure	hypersensitivity
congestive heart failure	edema
congestive heart failure	cough
congestive heart failure	hepatic failure
congestive heart failure	proteinuria
congestive heart failure	ulcer
congestive heart failure	pancytopenia
congestive heart failure	pain
conjunctivitis	epistaxis
conjunctivitis	upper respiratory tract infection
conjunctivitis	pneumonia
conjunctivitis	infection
conjunctivitis	pancreatitis
conjunctivitis	hypersensitivity
conjunctivitis	fever
conjunctivitis	hematuria
conjunctivitis	toxic epidermal necrolysis
conjunctivitis	ulcer
conjunctivitis	hepatitis
conjunctivitis	malaise
conjunctivitis	purpura
conjunctivitis	gastroenteritis
conjunctivitis	neutropenia
conjunctivitis	peripheral neuropathy
conjunctivitis	thrombocytopenia
conjunctivitis	muscle cramps
conjunctivitis	pancytopenia
conjunctivitis	sepsis
constipation	galactorrhea
constipation	syncope
constipation	increased salivation
constipation	urinary retention
constipation	dyskinesia
constipation	neurosis
constipation	weakness
constipation	impotence
constipation	torticollis
constipation	tachycardia
constipation	dysphagia
constipation	hypothyroidism
constipation	menorrhagia
constipation	paresthesia
constipation	weight gain
constipation	fatigue
constipation	glycosuria
constipation	nightmares
constipation	priapism
constitutional symptoms	stomatitis
constitutional symptoms	leukopenia
constitutional symptoms	neutropenia
constitutional symptoms	dyspnea
constitutional symptoms	neuropathy
constitutional symptoms	hemorrhage

constitutional symptoms	pleural effusion
constitutional symptoms	erythema
constitutional symptoms	infection
constitutional symptoms	pain
constitutional symptoms	peripheral edema
constitutional symptoms	weight loss
constitutional symptoms	peripheral neuropathy
constitutional symptoms	cough
constitutional symptoms	heart failure
contact dermatitis	hemorrhoids
contact dermatitis	neck rigidity
contact dermatitis	polyuria
contact dermatitis	tenesmus
contact dermatitis	vaginal hemorrhage
contact dermatitis	vaginitis
cough	dyspnea
cough	infection
cough	leukopenia
cough	neutropenia
cough	stomatitis
cough	flushing
cough	malaise
cystitis	ecchymosis
cystitis	hematemesis
cystitis	interstitial nephritis
cystitis	rectal hemorrhage
cystitis	herpes simplex
cystitis	eructation
cystitis	gastritis
cystitis	lymphadenopathy
cystitis	peptic ulcer
cystitis	exfoliative dermatitis
cystitis	toxic epidermal necrolysis
cystitis	glossitis
cystitis	hearing loss
cystitis	melena
cystitis	tinnitus
cystitis	gastrointestinal hemorrhage
cystitis	erythema multiforme
cystitis	dysuria
cystitis	flatulence
cystitis	hepatic failure
cystitis	proteinuria
cystitis	heartburn
cystitis	ulcerative stomatitis
cystitis	hemolytic anemia
cystitis	hepatitis
cystitis	pancytopenia
cystitis	sepsis
cystitis	hematuria
cystitis	jaundice
cystitis	pancreatitis
cystitis	esophagitis
cystitis	myocardial infarction

cystitis	stomatitis
deafness	fecal incontinence
deafness	hyperhidrosis
deafness	ischemic colitis
deafness	leukocytosis
deafness	neuralgia
deafness	skin ulcer
deafness	suicide attempt
decreased libido	dizziness
decreased libido	lightheadedness
decreased libido	sore throat
decreased libido	mental depression
decreased libido	psoriasis
decreased libido	heart block
decreased libido	fatigue
decreased libido	nightmares
decreased libido	hypokalemia
decreased libido	insomnia
dehydration	heart failure
dehydration	weight loss
delirium	tachycardia
delirium	hallucinations
delirium	urinary incontinence
delirium	tremor
delirium	torticollis
delirium	galactorrhea
delirium	laryngitis
delirium	priapism
delirium	increased salivation
delirium	somnolence
delirium	tardive dyskinesia
delirium	urinary retention
delirium	dysmenorrhea
delirium	dysphagia
delirium	goiter
delirium	personality disorder
delirium	urinary urgency
delirium	dyskinesia
delirium	hypotension
delirium	paralytic ileus
delirium	grand mal
delirium	paranoia
delirium	sleep disorder
delirium	ventricular fibrillation
delirium	ventricular tachycardia
dermatitis	stomatitis
dermatitis	infection
dermatitis	pancreatitis
dermatitis	hepatitis
dermatitis	neuropathy
dermatitis	vomiting
dermatitis	erythema
dermatitis	leukopenia
dermatitis	hypersensitivity

dermatitis	rash
dermatitis	myalgia
dermatitis	pleural effusion
diarrhea	rash
diarrhea	thrombocytopenia
diplopia	hiccup
diplopia	hallucinations
diplopia	laryngitis
diplopia	urinary incontinence
diplopia	tremor
diplopia	otitis media
diplopia	phlebitis
diplopia	hypotension
diplopia	dysarthria
diplopia	tachycardia
diplopia	glossitis
diplopia	hypoxia
diplopia	hyperglycemia
diplopia	shock
diplopia	liver function tests abnormal
diplopia	migraine
diplopia	herpes simplex
diplopia	urinary frequency
diplopia	eructation
diplopia	paralysis
diplopia	pulmonary embolism
diplopia	sleep disorder
diplopia	ventricular fibrillation
dizziness	fatigue
dizziness	lightheadedness
dizziness	sore throat
dizziness	mental depression
dizziness	psoriasis
dizziness	weakness
dizziness	flushing
dizziness	heart block
dizziness	nightmares
dizziness	syncope
drug dependence	fecal impaction
drug dependence	glaucoma
drug dependence	hyperacusis
drug dependence	hypothermia
drug dependence	hypoventilation
drug dependence	labyrinthitis
drug dependence	pyelonephritis
drug dependence	sneezing
drug dependence	stridor
drug dependence	urinary hesitancy
drug dependence	ventricular extrasystoles
drug dependence	voice alteration
drug dependence	wheezing
dry mouth	priapism
dry mouth	increased salivation
dry mouth	tardive dyskinesia

dry mouth	spasm
dry mouth	sweating
dry mouth	palpitations
dry mouth	paralytic ileus
dry mouth	heart block
dry mouth	torticollis
dry mouth	impotence
dry mouth	nasal congestion
dry mouth	tachycardia
dry mouth	galactorrhea
dry mouth	nightmares
dry mouth	postural hypotension
dry mouth	grand mal
dry mouth	paranoia
dry mouth	ventricular tachycardia
dry mouth	somnolence
dry mouth	urinary retention
dry mouth	dyskinesia
dry mouth	psoriasis
dry mouth	tremor
dry mouth	syncope
dry mouth	dysmenorrhea
dry mouth	goiter
dry mouth	keratitis
dry mouth	personality disorder
dry mouth	serotonin syndrome
dry mouth	supraventricular extrasystoles
dry mouth	weight gain
dry mouth	gynecomastia
dry mouth	hypotension
dry mouth	insomnia
dry mouth	lightheadedness
dry mouth	photophobia
dry mouth	sore throat
dry skin	gastritis
dry skin	gastrointestinal hemorrhage
dry skin	hematemesis
dry skin	interstitial nephritis
dry skin	rectal hemorrhage
dry skin	liver function tests abnormal
dry skin	migraine
dry skin	eructation
dry skin	otitis media
dry skin	phlebitis
dry skin	pulmonary embolism
dry skin	exfoliative dermatitis
dry skin	toxic epidermal necrolysis
dry skin	dysarthria
dry skin	hiccup
dry skin	lymphadenopathy
dry skin	neurosis
dry skin	stomatitis
dysarthria	hiccup
dysarthria	tremor

dysarthria	hypoxia
dysarthria	hallucinations
dysarthria	laryngitis
dysarthria	urinary incontinence
dysarthria	liver function tests abnormal
dysarthria	migraine
dysarthria	hypotension
dysarthria	otitis media
dysarthria	phlebitis
dysarthria	pulmonary embolism
dysarthria	tachycardia
dysarthria	lymphadenopathy
dysarthria	nervousness
dysarthria	neurosis
dyskinesia	increased salivation
dyskinesia	urinary retention
dyskinesia	torticollis
dyskinesia	galactorrhea
dyskinesia	priapism
dyskinesia	syncope
dyskinesia	impotence
dyskinesia	tachycardia
dyskinesia	tardive dyskinesia
dyskinesia	nervousness
dyskinesia	postural hypotension
dyskinesia	insomnia
dyskinesia	paralytic ileus
dyskinesia	nasal congestion
dyskinesia	nightmares
dyskinesia	grand mal
dyskinesia	paranoia
dyskinesia	photophobia
dyskinesia	ventricular tachycardia
dyskinesia	tremor
dyskinesia	somnolence
dyskinesia	hypotension
dyskinesia	palpitations
dyskinesia	gynecomastia
dyskinesia	neurosis
dyskinesia	psoriasis
dyskinesia	spasm
dyskinesia	sweating
dyskinesia	heart block
dyskinesia	dysmenorrhea
dyskinesia	dysphagia
dyskinesia	goiter
dyskinesia	hypothyroidism
dyskinesia	keratitis
dyskinesia	menorrhagia
dyskinesia	personality disorder
dyskinesia	psychosis
dyskinesia	serotonin syndrome
dyskinesia	supraventricular extrasystoles
dyskinesia	urinary urgency

dyskinesia	weight gain
dyskinesia	glycosuria
dyskinesia	hallucinations
dyskinesia	urinary incontinence
dysmenorrhea	goiter
dysmenorrhea	personality disorder
dysmenorrhea	grand mal
dysmenorrhea	paranoia
dysmenorrhea	ventricular tachycardia
dysmenorrhea	psoriasis
dysmenorrhea	seizures
dysmenorrhea	spasm
dysmenorrhea	sweating
dysmenorrhea	heart block
dysmenorrhea	torticollis
dysmenorrhea	galactorrhea
dysmenorrhea	hallucinations
dysmenorrhea	nightmares
dysmenorrhea	priapism
dysmenorrhea	syncope
dysmenorrhea	urinary incontinence
dysmenorrhea	increased salivation
dysmenorrhea	somnolence
dysmenorrhea	tardive dyskinesia
dysmenorrhea	urinary retention
dysmenorrhea	hypotension
dysmenorrhea	insomnia
dysmenorrhea	palpitations
dysmenorrhea	paralytic ileus
dysmenorrhea	impotence
dysmenorrhea	nasal congestion
Dyspepsia	abdominal pain
Dyspepsia	conjunctivitis
Dyspepsia	epistaxis
Dyspepsia	angioedema
Dyspepsia	infection
Dyspepsia	pancreatitis
Dyspepsia	toxic epidermal necrolysis
Dyspepsia	alopecia
Dyspepsia	erythema multiforme
Dyspepsia	malaise
Dyspepsia	hepatitis
Dyspepsia	hypersensitivity
Dyspepsia	Sinusitis
Dyspepsia	pneumonia
Dyspepsia	vertigo
Dyspepsia	flatulence
Dyspepsia	upper respiratory tract infection
Dyspepsia	exfoliative dermatitis
Dyspepsia	Influenza
Dyspepsia	Pharyngitis
Dyspepsia	hematemesis
Dyspepsia	interstitial nephritis
Dyspepsia	photosensitivity

Dyspepsia	rectal hemorrhage
Dyspepsia	purpura
Dyspepsia	anaphylaxis
Dyspepsia	rash
Dyspepsia	asthenia
Dyspepsia	Stevens - Johnson syndrome
Dyspepsia	myalgia
Dyspepsia	proteinuria
Dyspepsia	ulcer
Dyspepsia	vasculitis
Dyspepsia	aplastic anemia
Dyspepsia	gastroenteritis
Dyspepsia	hearing loss
Dyspepsia	hyperkalemia
Dyspepsia	melena
Dyspepsia	tinnitus
Dyspepsia	rhinitis
dysphagia	torticollis
dysphagia	galactorrhea
dysphagia	priapism
dysphagia	increased salivation
dysphagia	tardive dyskinesia
dysphagia	urinary retention
dysphagia	paralytic ileus
dysphagia	weight loss
dyspnea	stomatitis
dyspnea	neutropenia
dyspnea	infection
dyspnea	leukopenia
dyspnea	hemorrhage
dyspnea	thrombocytopenia
dyspnea	pancreatitis
dyspnea	edema
dyspnea	erythema multiforme
dyspnea	hypersensitivity
dyspnea	neuropathy
dyspnea	renal failure
dyspnea	hematuria
dyspnea	malaise
dyspnea	toxic epidermal necrolysis
dyspnea	hyperkalemia
dyspnea	peripheral edema
dysuria	eructation
dysuria	herpes simplex
dysuria	peptic ulcer
dysuria	gastrointestinal hemorrhage
dysuria	gastritis
dysuria	glossitis
dysuria	hearing loss
dysuria	melena
dysuria	pancytopenia
dysuria	sepsis
dysuria	ecchymosis
dysuria	hematemesis

dysuria	interstitial nephritis
dysuria	rectal hemorrhage
dysuria	hepatic failure
dysuria	proteinuria
dysuria	hemolytic anemia
dysuria	esophagitis
dysuria	lymphadenopathy
dysuria	hypoxia
dysuria	tinnitus
dysuria	hematuria
dysuria	stomatitis
dysuria	tremor
dysuria	heartburn
dysuria	exfoliative dermatitis
dysuria	toxic epidermal necrolysis
dysuria	hepatitis
dysuria	flatulence
dysuria	ulcer
dysuria	vasculitis
ecchymosis	gastritis
ecchymosis	hematemesis
ecchymosis	interstitial nephritis
ecchymosis	rectal hemorrhage
ecchymosis	toxic epidermal necrolysis
ecchymosis	herpes simplex
ecchymosis	gastrointestinal hemorrhage
ecchymosis	esophagitis
ecchymosis	peptic ulcer
ecchymosis	eructation
ecchymosis	hearing loss
ecchymosis	melena
ecchymosis	pancytopenia
ecchymosis	sepsis
ecchymosis	hematuria
ecchymosis	jaundice
ecchymosis	hepatitis
ecchymosis	exfoliative dermatitis
ecchymosis	flatulence
ecchymosis	hepatic failure
ecchymosis	proteinuria
ecchymosis	ulcer
ecchymosis	hemolytic anemia
ecchymosis	pancreatitis
ecchymosis	erythema multiforme
ecchymosis	pneumonia
ecchymosis	lymphadenopathy
ecchymosis	pruritus
ecchymosis	stomatitis
ecchymosis	gastroenteritis
ecchymosis	glossitis
ecchymosis	tinnitus
ecchymosis	photosensitivity
ecchymosis	heartburn
ecchymosis	ulcerative stomatitis

ecchymosis	vasculitis
ecchymosis	renal failure
ecchymosis	thrombocytopenia
eczema	purpura
eczema	weight gain
eczema	flatulence
eczema	insomnia
eczema	palpitations
eczema	grand mal
eczema	paranoia
eczema	ventricular tachycardia
edema	fever
edema	hematuria
edema	purpura
edema	infection
elevated liver enzymes	pelvic pain
eosinophilia	pancytopenia
eosinophilia	purpura
eosinophilia	jaundice
eosinophilia	exfoliative dermatitis
eosinophilia	esophagitis
eosinophilia	peptic ulcer
eosinophilia	pruritus
epistaxis	pancreatitis
epistaxis	infection
epistaxis	upper respiratory tract infection
epistaxis	toxic epidermal necrolysis
epistaxis	malaise
epistaxis	erythema multiforme
epistaxis	peripheral neuropathy
epistaxis	fever
epistaxis	purpura
epistaxis	pneumonia
epistaxis	hepatitis
epistaxis	hypersensitivity
epistaxis	muscle cramps
epistaxis	gastroenteritis
epistaxis	hematuria
epistaxis	jaundice
eructation	herpes simplex
eructation	glossitis
eructation	gastrointestinal hemorrhage
eructation	gastritis
eructation	hematemesis
eructation	interstitial nephritis
eructation	rectal hemorrhage
eructation	lymphadenopathy
eructation	peptic ulcer
eructation	hearing loss
eructation	hypoxia
eructation	melena
eructation	tinnitus
eructation	hallucinations
eructation	laryngitis

eructation	urinary incontinence
eructation	otitis media
eructation	phlebitis
eructation	sleep disorder
eructation	tremor
eructation	flatulence
eructation	hepatic failure
eructation	proteinuria
eructation	urinary frequency
eructation	exfoliative dermatitis
eructation	toxic epidermal necrolysis
eructation	hemolytic anemia
eructation	esophagitis
eructation	hiccup
eructation	hepatitis
eructation	erythema multiforme
eructation	pancytopenia
eructation	sepsis
eructation	hematuria
eructation	tachycardia
eructation	heartburn
eructation	liver function tests abnormal
eructation	migraine
eructation	ulcerative stomatitis
eructation	hyperglycemia
eructation	photosensitivity
eructation	jaundice
eructation	stomatitis
erythema	neutropenia
erythema	stomatitis
erythema	peripheral edema
erythema	leukopenia
erythema	hemorrhage
erythema	pleural effusion
erythema	weight loss
erythema	neuropathy
erythema multiforme	exfoliative dermatitis
erythema multiforme	toxic epidermal necrolysis
erythema multiforme	pancreatitis
erythema multiforme	peripheral neuropathy
erythema multiforme	hematemesis
erythema multiforme	interstitial nephritis
erythema multiforme	rectal hemorrhage
erythema multiforme	gastrointestinal hemorrhage
erythema multiforme	gastritis
erythema multiforme	renal failure
erythema multiforme	hearing loss
erythema multiforme	melena
erythema multiforme	sepsis
erythema multiforme	stomatitis
erythema multiforme	hematuria
erythema multiforme	peptic ulcer
erythema multiforme	leukopenia
erythema multiforme	hepatic failure

erythema multiforme	proteinuria
erythema multiforme	vasculitis
erythema multiforme	thrombocytopenia
erythema multiforme	infection
erythema multiforme	tinnitus
erythema multiforme	hemolytic anemia
erythema multiforme	hemorrhage
erythema multiforme	pneumonia
erythema multiforme	photosensitivity
erythema multiforme	pulmonary edema
erythema multiforme	pancytopenia
esophagitis	ulcer
esophagitis	gastritis
esophagitis	pneumonia
esophagitis	pancytopenia
esophagitis	sepsis
esophagitis	hematuria
esophagitis	peptic ulcer
esophagitis	pruritus
esophagitis	toxic epidermal necrolysis
esophagitis	gastroenteritis
esophagitis	hearing loss
esophagitis	melena
esophagitis	gastrointestinal hemorrhage
esophagitis	hematemesis
esophagitis	interstitial nephritis
esophagitis	rectal hemorrhage
esophagitis	thrombocytopenia
esophagitis	flatulence
esophagitis	hepatic failure
esophagitis	herpes simplex
esophagitis	proteinuria
esophagitis	vasculitis
esophagitis	hemolytic anemia
esophagitis	renal failure
esophagitis	hepatitis
esophagitis	jaundice
esophagitis	pancreatitis
esophagitis	exfoliative dermatitis
esophagitis	lymphadenopathy
esophagitis	stomatitis
exfoliative dermatitis	hematemesis
exfoliative dermatitis	interstitial nephritis
exfoliative dermatitis	rectal hemorrhage
exfoliative dermatitis	gastritis
exfoliative dermatitis	hearing loss
exfoliative dermatitis	melena
exfoliative dermatitis	pancreatitis
exfoliative dermatitis	toxic epidermal necrolysis
exfoliative dermatitis	peptic ulcer
exfoliative dermatitis	hepatic failure
exfoliative dermatitis	proteinuria
exfoliative dermatitis	vasculitis
exfoliative dermatitis	purpura

exfoliative dermatitis	hemolytic anemia
exfoliative dermatitis	tinnitus
exfoliative dermatitis	pneumonia
exfoliative dermatitis	renal failure
exfoliative dermatitis	photosensitivity
exfoliative dermatitis	gastrointestinal hemorrhage
exfoliative dermatitis	pancytopenia
exfoliative dermatitis	sepsis
exfoliative dermatitis	hematuria
exfoliative dermatitis	flatulence
exfoliative dermatitis	herpes simplex
exfoliative dermatitis	ulcer
exfoliative dermatitis	lymphadenopathy
exfoliative dermatitis	jaundice
exfoliative dermatitis	hepatitis
fatigue	psoriasis
fatigue	heart block
fatigue	nightmares
fatigue	syncope
fatigue	weight gain
fatigue	insomnia
fatigue	palpitations
fatigue	flushing
fatigue	grand mal
fatigue	lightheadedness
fatigue	paranoia
fatigue	sore throat
fatigue	ventricular tachycardia
fecal impaction	glaucoma
fecal impaction	hyperacusis
fecal impaction	hypothermia
fecal impaction	hypoventilation
fecal impaction	labyrinthitis
fecal impaction	pyelonephritis
fecal impaction	sneezing
fecal impaction	stridor
fecal impaction	urinary hesitancy
fecal impaction	ventricular extrasystoles
fecal impaction	voice alteration
fecal impaction	wheezing
fecal incontinence	hyperhidrosis
fecal incontinence	ischemic colitis
fecal incontinence	leukocytosis
fecal incontinence	neuralgia
fecal incontinence	skin ulcer
fecal incontinence	suicide attempt
fever	infection
fever	malaise
fever	hypersensitivity
fever	upper respiratory tract infection
fever	pancreatitis
fever	stomatitis
flatulence	hematemesis
flatulence	interstitial nephritis

flatulence	photosensitivity
flatulence	rectal hemorrhage
flatulence	ulcer
flatulence	vasculitis
flatulence	toxic epidermal necrolysis
flatulence	hepatitis
flatulence	lymphadenopathy
flatulence	peptic ulcer
flatulence	pruritus
flatulence	gastritis
flatulence	renal failure
flatulence	thrombocytopenia
flatulence	hearing loss
flatulence	melena
flatulence	tinnitus
flatulence	purpura
flatulence	jaundice
flatulence	urticaria
flatulence	hepatic failure
flatulence	herpes simplex
flatulence	proteinuria
flatulence	gastrointestinal hemorrhage
flushing	hypokalemia
flushing	pulmonary edema
galactorrhea	increased salivation
galactorrhea	urinary retention
galactorrhea	tachycardia
galactorrhea	nightmares
galactorrhea	priapism
galactorrhea	tardive dyskinesia
galactorrhea	sweating
galactorrhea	paralytic ileus
galactorrhea	heart block
galactorrhea	torticollis
galactorrhea	impotence
galactorrhea	syncope
galactorrhea	grand mal
galactorrhea	paranoia
galactorrhea	ventricular tachycardia
galactorrhea	hypotension
galactorrhea	insomnia
galactorrhea	palpitations
galactorrhea	nervousness
galactorrhea	neurosis
galactorrhea	psoriasis
galactorrhea	spasm
galactorrhea	nasal congestion
galactorrhea	postural hypotension
galactorrhea	tremor
galactorrhea	glycosuria
galactorrhea	hallucinations
galactorrhea	urinary incontinence
galactorrhea	goiter
galactorrhea	hypothyroidism

galactorrhea	keratitis
galactorrhea	menorrhagia
galactorrhea	personality disorder
galactorrhea	serotonin syndrome
galactorrhea	supraventricular extrasystoles
galactorrhea	urinary urgency
galactorrhea	weight gain
galactorrhea	gynecomastia
galactorrhea	somnolence
galactorrhea	lightheadedness
galactorrhea	photophobia
galactorrhea	sore throat
gastritis	hematemesis
gastritis	interstitial nephritis
gastritis	rectal hemorrhage
gastritis	gastrointestinal hemorrhage
gastritis	herpes simplex
gastritis	hearing loss
gastritis	melena
gastritis	toxic epidermal necrolysis
gastritis	stomatitis
gastritis	sepsis
gastritis	hematuria
gastritis	lymphadenopathy
gastritis	peptic ulcer
gastritis	hepatic failure
gastritis	proteinuria
gastritis	ulcer
gastritis	pancreatitis
gastritis	glossitis
gastritis	pneumonia
gastritis	pancytopenia
gastritis	hepatitis
gastritis	vasculitis
gastritis	jaundice
gastritis	hemolytic anemia
gastritis	pruritus
gastritis	renal failure
gastritis	gastroenteritis
gastritis	tinnitus
gastritis	heartburn
gastritis	liver function tests abnormal
gastritis	migraine
gastritis	ulcerative stomatitis
gastritis	thrombocytopenia
gastritis	photosensitivity
gastroenteritis	pneumonia
gastroenteritis	hepatitis
gastroenteritis	hyperkalemia
gastroenteritis	toxic epidermal necrolysis
gastroenteritis	pancreatitis
gastroenteritis	herpes simplex
gastroenteritis	proteinuria
gastroenteritis	ulcer

gastroenteritis	hemolytic anemia
gastroenteritis	hypersensitivity
gastroenteritis	pancytopenia
gastroenteritis	sepsis
gastroenteritis	hematuria
gastroesophageal reflux	gingivitis
gastrointestinal hemorrhage	toxic epidermal necrolysis
gastrointestinal hemorrhage	herpes simplex
gastrointestinal hemorrhage	sepsis
gastrointestinal hemorrhage	hematemesis
gastrointestinal hemorrhage	interstitial nephritis
gastrointestinal hemorrhage	rectal hemorrhage
gastrointestinal hemorrhage	glossitis
gastrointestinal hemorrhage	hearing loss
gastrointestinal hemorrhage	melena
gastrointestinal hemorrhage	hematuria
gastrointestinal hemorrhage	stomatitis
gastrointestinal hemorrhage	pneumonia
gastrointestinal hemorrhage	renal failure
gastrointestinal hemorrhage	lymphadenopathy
gastrointestinal hemorrhage	peptic ulcer
gastrointestinal hemorrhage	thrombocytopenia
gastrointestinal hemorrhage	hepatic failure
gastrointestinal hemorrhage	proteinuria
gastrointestinal hemorrhage	ulcer
gastrointestinal hemorrhage	tinnitus
gastrointestinal hemorrhage	peripheral neuropathy
gastrointestinal hemorrhage	otitis media
gastrointestinal hemorrhage	phlebitis
gastrointestinal hemorrhage	hepatitis
gastrointestinal hemorrhage	pancytopenia
gastrointestinal hemorrhage	leukopenia
gastrointestinal hemorrhage	heart failure
gastrointestinal hemorrhage	vasculitis
gastrointestinal hemorrhage	hiccup
gastrointestinal hemorrhage	nocturia
gastrointestinal hemorrhage	pruritus
glaucoma	hyperacusis
glaucoma	hypothermia
glaucoma	hypoventilation
glaucoma	labyrinthitis
glaucoma	pyelonephritis
glaucoma	sneezing
glaucoma	stridor
glaucoma	urinary hesitancy
glaucoma	ventricular extrasystoles
glaucoma	voice alteration
glaucoma	wheezing
glossitis	herpes simplex
glossitis	otitis media
glossitis	phlebitis
glossitis	hiccup
glossitis	lymphadenopathy
glossitis	tremor

glossitis	hypoxia
glossitis	tinnitus
glossitis	hallucinations
glossitis	hematemesis
glossitis	hyperglycemia
glossitis	interstitial nephritis
glossitis	laryngitis
glossitis	rectal hemorrhage
glossitis	urinary incontinence
glossitis	urinary frequency
glossitis	heartburn
glossitis	liver function tests abnormal
glossitis	migraine
glossitis	ulcerative stomatitis
glossitis	hypotension
glossitis	pulmonary embolism
glossitis	sleep disorder
glossitis	ventricular fibrillation
glossitis	tachycardia
glossitis	hepatitis
glycosuria	hyperglycemia
glycosuria	increased salivation
glycosuria	impotence
glycosuria	tachycardia
glycosuria	priapism
glycosuria	paresthesia
glycosuria	tardive dyskinesia
glycosuria	urinary retention
glycosuria	palpitations
glycosuria	paralytic ileus
glycosuria	paralysis
glycosuria	rhabdomyolysis
goiter	personality disorder
goiter	grand mal
goiter	paranoia
goiter	ventricular tachycardia
goiter	psoriasis
goiter	seizures
goiter	spasm
goiter	sweating
goiter	heart block
goiter	torticollis
goiter	hallucinations
goiter	nightmares
goiter	priapism
goiter	syncope
goiter	urinary incontinence
goiter	increased salivation
goiter	somnolence
goiter	tardive dyskinesia
goiter	urinary retention
goiter	hypotension
goiter	insomnia
goiter	palpitations

goiter	paralytic ileus
goiter	impotence
goiter	nasal congestion
gout	rhinitis
gout	hyponatremia
gout	hyperkalemia
gout	hemolytic anemia
gout	palpitations
gout	muscle cramps
grand mal	paranoia
grand mal	ventricular tachycardia
grand mal	psoriasis
grand mal	sweating
grand mal	heart block
grand mal	torticollis
grand mal	nightmares
grand mal	priapism
grand mal	syncope
grand mal	increased salivation
grand mal	somnolence
grand mal	tardive dyskinesia
grand mal	urinary retention
grand mal	hypotension
grand mal	insomnia
grand mal	palpitations
grand mal	paralytic ileus
grand mal	keratitis
grand mal	personality disorder
grand mal	serotonin syndrome
grand mal	supraventricular extrasystoles
grand mal	weight gain
grand mal	impotence
grand mal	nasal congestion
grand mal	tachycardia
grand mal	postural hypotension
grand mal	tremor
grand mal	photophobia
grand mal	nervousness
grand mal	neurosis
grand mal	seizures
grand mal	spasm
grand mal	hallucinations
grand mal	urinary incontinence
gynecomastia	paralytic ileus
gynecomastia	tardive dyskinesia
gynecomastia	nasal congestion
gynecomastia	priapism
gynecomastia	increased salivation
gynecomastia	torticollis
gynecomastia	postural hypotension
gynecomastia	urinary retention
gynecomastia	tachycardia
gynecomastia	spasm
gynecomastia	sweating

hallucinations	urinary incontinence
hallucinations	tachycardia
hallucinations	laryngitis
hallucinations	tremor
hallucinations	hiccup
hallucinations	hypotension
hallucinations	hypoxia
hallucinations	otitis media
hallucinations	phlebitis
hallucinations	sleep disorder
hallucinations	ventricular fibrillation
hallucinations	somnolence
hallucinations	urinary frequency
hallucinations	neurosis
hallucinations	nightmares
hallucinations	syncope
hallucinations	hypothyroidism
hallucinations	liver function tests abnormal
hallucinations	menorrhagia
hallucinations	migraine
hallucinations	personality disorder
hallucinations	herpes simplex
hallucinations	increased salivation
hallucinations	urinary retention
hallucinations	insomnia
hallucinations	paranoia
hallucinations	pulmonary embolism
hallucinations	ventricular tachycardia
hearing loss	melena
hearing loss	hematemesis
hearing loss	interstitial nephritis
hearing loss	rectal hemorrhage
hearing loss	hepatic failure
hearing loss	proteinuria
hearing loss	peptic ulcer
hearing loss	sepsis
hearing loss	hematuria
hearing loss	toxic epidermal necrolysis
hearing loss	ulcer
hearing loss	vasculitis
hearing loss	hemolytic anemia
hearing loss	pneumonia
hearing loss	renal failure
hearing loss	pancytopenia
hearing loss	pancreatitis
hearing loss	herpes simplex
hearing loss	heartburn
hearing loss	stomatitis
hearing loss	thrombocytopenia
hearing loss	hepatitis
heart block	nightmares
heart block	psoriasis
heart block	sweating
heart block	priapism

heart block	increased salivation
heart block	tardive dyskinesia
heart block	urinary retention
heart block	lightheadedness
heart block	paranoia
heart block	sore throat
heart block	ventricular tachycardia
heart block	insomnia
heart block	palpitations
heart block	paralytic ileus
heart block	mental depression
heart block	spasm
heart block	impotence
heart block	nasal congestion
heart block	tachycardia
heart block	postural hypotension
heart block	torticollis
heart block	syncope
heart block	somnolence
heart block	keratitis
heart block	memory loss
heart block	personality disorder
heart block	serotonin syndrome
heart block	supraventricular extrasystoles
heart block	weight gain
heart block	hypotension
heart block	photophobia
heart failure	neuropathy
heart failure	peripheral neuropathy
heart failure	toxic epidermal necrolysis
heart failure	pleural effusion
heart failure	leukopenia
heart failure	thrombocytopenia
heart failure	hypersensitivity
heart failure	stomatitis
heart failure	infection
heart failure	neutropenia
heartburn	peptic ulcer
heartburn	melena
heartburn	hematemesis
heartburn	interstitial nephritis
heartburn	rectal hemorrhage
heartburn	hepatic failure
heartburn	herpes simplex
heartburn	proteinuria
heartburn	hemolytic anemia
heartburn	pancytopenia
heartburn	sepsis
hematemesis	interstitial nephritis
hematemesis	rectal hemorrhage
hematemesis	melena
hematemesis	toxic epidermal necrolysis
hematemesis	hepatic failure
hematemesis	proteinuria

hematemesis	peptic ulcer
hematemesis	sepsis
hematemesis	hematuria
hematemesis	herpes simplex
hematemesis	ulcer
hematemesis	vasculitis
hematemesis	hemolytic anemia
hematemesis	pancreatitis
hematemesis	pneumonia
hematemesis	renal failure
hematemesis	lymphadenopathy
hematemesis	stomatitis
hematemesis	pancytopenia
hematemesis	tinnitus
hematemesis	jaundice
hematemesis	hepatitis
hematemesis	photosensitivity
hematemesis	ulcerative stomatitis
hematemesis	thrombocytopenia
hematuria	pneumonia
hematuria	sepsis
hematuria	melena
hematuria	stomatitis
hematuria	toxic epidermal necrolysis
hematuria	interstitial nephritis
hematuria	rectal hemorrhage
hematuria	pancytopenia
hematuria	peptic ulcer
hematuria	hepatic failure
hematuria	proteinuria
hematuria	ulcer
hematuria	pancreatitis
hematuria	thrombocytopenia
hematuria	hypersensitivity
hematuria	herpes simplex
hematuria	vasculitis
hematuria	hepatitis
hematuria	peripheral neuropathy
hematuria	jaundice
hematuria	pruritus
hematuria	leukopenia
hematuria	hemolytic anemia
hematuria	infection
hematuria	renal failure
hematuria	vertigo
hemolytic anemia	proteinuria
hemolytic anemia	vasculitis
hemolytic anemia	peptic ulcer
hemolytic anemia	hyperkalemia
hemolytic anemia	melena
hemolytic anemia	hepatitis
hemolytic anemia	interstitial nephritis
hemolytic anemia	rectal hemorrhage
hemolytic anemia	pancreatitis

hemolytic anemia	hepatic failure
hemolytic anemia	toxic epidermal necrolysis
hemolytic anemia	hyponatremia
hemolytic anemia	pneumonia
hemolytic anemia	renal failure
hemolytic anemia	pancytopenia
hemolytic anemia	sepsis
hemolytic anemia	tinnitus
hemolytic anemia	vertigo
hemolytic anemia	hyperuricemia
hemolytic anemia	photosensitivity
hemolytic anemia	jaundice
hemoptysis	mouth ulceration
hemoptysis	petechiae
hemoptysis	swelling
hemorrhage	stomatitis
hemorrhage	infection
hemorrhage	pancreatitis
hemorrhage	peripheral edema
hemorrhoids	neck rigidity
hemorrhoids	polyuria
hemorrhoids	tenesmus
hemorrhoids	vaginal hemorrhage
hemorrhoids	vaginitis
hepatic failure	melena
hepatic failure	sepsis
hepatic failure	interstitial nephritis
hepatic failure	rectal hemorrhage
hepatic failure	proteinuria
hepatic failure	peptic ulcer
hepatic failure	ulcer
hepatic failure	vasculitis
hepatic failure	toxic epidermal necrolysis
hepatic failure	pneumonia
hepatic failure	renal failure
hepatic failure	pancytopenia
hepatic failure	thrombocytopenia
hepatic failure	hyperkalemia
hepatic failure	stomatitis
hepatic failure	jaundice
hepatic failure	herpes simplex
hepatitis	jaundice
hepatitis	pancreatitis
hepatitis	toxic epidermal necrolysis
hepatitis	pneumonia
hepatitis	herpes simplex
hepatitis	thrombocytopenia
hepatitis	pancytopenia
hepatitis	interstitial nephritis
hepatitis	photosensitivity
hepatitis	rectal hemorrhage
hepatitis	lymphadenopathy
hepatitis	peptic ulcer
hepatitis	pruritus

hepatitis	upper respiratory tract infection
hepatitis	proteinuria
hepatitis	vasculitis
hepatitis	muscle cramps
hepatitis	sepsis
hepatitis	hyperkalemia
hepatitis	melena
hepatitis	tinnitus
hepatitis	vertigo
herpes simplex	lymphadenopathy
herpes simplex	tinnitus
herpes simplex	interstitial nephritis
herpes simplex	rectal hemorrhage
herpes simplex	otitis media
herpes simplex	phlebitis
herpes simplex	toxic epidermal necrolysis
herpes simplex	hiccup
herpes simplex	peptic ulcer
herpes simplex	pancytopenia
herpes simplex	sepsis
herpes simplex	hypoxia
herpes simplex	melena
herpes simplex	stomatitis
herpes simplex	jaundice
herpes simplex	tremor
herpes simplex	hyperglycemia
herpes simplex	laryngitis
herpes simplex	urinary incontinence
herpes simplex	liver function tests abnormal
herpes simplex	migraine
herpes simplex	ulcerative stomatitis
herpes simplex	proteinuria
herpes simplex	ulcer
herpes simplex	urinary frequency
herpes zoster	menstrual disorder
hiccup	laryngitis
hiccup	urinary incontinence
hiccup	hypotension
hiccup	otitis media
hiccup	phlebitis
hiccup	tachycardia
hiccup	tremor
hiccup	hypoxia
hiccup	urinary frequency
hiccup	liver function tests abnormal
hiccup	migraine
hiccup	pulmonary embolism
hiccup	sleep disorder
hiccup	ventricular fibrillation
hiccup	lymphadenopathy
hiccup	nervousness
hiccup	neurosis
hyperacusis	hypothermia
hyperacusis	hypoventilation

hyperacusis	labyrinthitis
hyperacusis	pyelonephritis
hyperacusis	sneezing
hyperacusis	stridor
hyperacusis	urinary hesitancy
hyperacusis	ventricular extrasystoles
hyperacusis	voice alteration
hyperacusis	wheezing
hypercholesterolemia	nephrotic syndrome
hyperglycemia	tinnitus
hyperglycemia	photosensitivity
hyperglycemia	paresthesia
hyperglycemia	urinary frequency
hyperglycemia	palpitations
hyperglycemia	otitis media
hyperglycemia	paralysis
hyperglycemia	phlebitis
hyperhidrosis	ischemic colitis
hyperhidrosis	leukocytosis
hyperhidrosis	neuralgia
hyperhidrosis	skin ulcer
hyperhidrosis	suicide attempt
hyperkalemia	tinnitus
hyperkalemia	proteinuria
hyperkalemia	vasculitis
hyperkalemia	palpitations
hyperkalemia	pneumonia
hyperkalemia	renal failure
hyperkalemia	rhinitis
hyperkalemia	sepsis
hypersensitivity	infection
hypersensitivity	pneumonia
hypersensitivity	thrombocytopenia
hypersensitivity	ulcer
hypersensitivity	stomatitis
hypersensitivity	toxic epidermal necrolysis
hypersensitivity	leukopenia
hypersensitivity	myalgia
hypersensitivity	peripheral neuropathy
hypersensitivity	neutropenia
hypersensitivity	malaise
hyperuricemia	vasculitis
hypoglycemia	hyponatremia
hypokalemia	pleural effusion
hypokalemia	pulmonary edema
hyponatremia	muscle cramps
hyponatremia	rhinitis
hypotension	urinary incontinence
hypotension	tachycardia
hypotension	neurosis
hypotension	tremor
hypotension	laryngitis
hypotension	nightmares
hypotension	syncope

hypotension	otitis media
hypotension	paranoia
hypotension	phlebitis
hypotension	ventricular fibrillation
hypotension	ventricular tachycardia
hypotension	increased salivation
hypotension	somnolence
hypotension	urinary frequency
hypotension	urinary retention
hypotension	insomnia
hypotension	palpitations
hypotension	nervousness
hypotension	psoriasis
hypotension	seizures
hypotension	sweating
hypotension	impotence
hypotension	hypoxia
hypotension	torticollis
hypotension	hypothyroidism
hypotension	keratitis
hypotension	liver function tests abnormal
hypotension	menorrhagia
hypotension	migraine
hypotension	personality disorder
hypotension	serotonin syndrome
hypotension	supraventricular extrasystoles
hypotension	weight gain
hypotension	priapism
hypothermia	hypoventilation
hypothermia	labyrinthitis
hypothermia	pyelonephritis
hypothermia	sneezing
hypothermia	stridor
hypothermia	urinary hesitancy
hypothermia	ventricular extrasystoles
hypothermia	voice alteration
hypothermia	wheezing
hypothyroidism	menorrhagia
hypothyroidism	neurosis
hypothyroidism	nightmares
hypothyroidism	syncope
hypothyroidism	urinary incontinence
hypothyroidism	increased salivation
hypothyroidism	urinary retention
hypothyroidism	insomnia
hypothyroidism	impotence
hypoventilation	labyrinthitis
hypoventilation	pyelonephritis
hypoventilation	sneezing
hypoventilation	stridor
hypoventilation	urinary hesitancy
hypoventilation	ventricular extrasystoles
hypoventilation	voice alteration
hypoventilation	wheezing

hypoxia	tremor
hypoxia	laryngitis
hypoxia	urinary incontinence
hypoxia	urinary frequency
hypoxia	otitis media
hypoxia	phlebitis
hypoxia	sleep disorder
hypoxia	tachycardia
hypoxia	liver function tests abnormal
hypoxia	migraine
hypoxia	pulmonary embolism
hypoxia	ventricular fibrillation
impotence	syncope
impotence	increased salivation
impotence	urinary retention
impotence	tachycardia
impotence	palpitations
impotence	postural hypotension
impotence	nightmares
impotence	priapism
impotence	nervousness
impotence	spasm
impotence	sweating
impotence	tardive dyskinesia
impotence	insomnia
impotence	paralytic ileus
impotence	torticollis
impotence	nasal congestion
impotence	paranoia
impotence	photophobia
impotence	ventricular tachycardia
impotence	somnolence
impotence	tremor
impotence	neurosis
impotence	psoriasis
impotence	weakness
impotence	keratitis
impotence	menorrhagia
impotence	paresthesia
impotence	personality disorder
impotence	psychosis
impotence	serotonin syndrome
impotence	supraventricular extrasystoles
impotence	weight gain
increased salivation	priapism
increased salivation	tachycardia
increased salivation	tardive dyskinesia
increased salivation	urinary retention
increased salivation	paralytic ileus
increased salivation	torticollis
increased salivation	nightmares
increased salivation	spasm
increased salivation	sweating
increased salivation	palpitations

increased salivation	nasal congestion
increased salivation	syncope
increased salivation	postural hypotension
increased salivation	paranoia
increased salivation	ventricular tachycardia
increased salivation	insomnia
increased salivation	nervousness
increased salivation	neurosis
increased salivation	psoriasis
increased salivation	tremor
increased salivation	urinary incontinence
increased salivation	keratitis
increased salivation	menorrhagia
increased salivation	personality disorder
increased salivation	serotonin syndrome
increased salivation	supraventricular extrasystoles
increased salivation	urinary urgency
increased salivation	weight gain
increased salivation	somnolence
increased sweating	paralysis
increased sweating	shock
infection	stomatitis
infection	pancreatitis
infection	peripheral neuropathy
infection	leukopenia
infection	neutropenia
infection	malaise
infection	toxic epidermal necrolysis
infection	myalgia
infection	ulcer
infection	neuropathy
infection	pneumonia
infection	thrombocytopenia
Influenza	conjunctivitis
Influenza	epistaxis
Influenza	upper respiratory tract infection
Influenza	abdominal pain
insomnia	nightmares
insomnia	syncope
insomnia	urinary retention
insomnia	nervousness
insomnia	psoriasis
insomnia	tremor
insomnia	paranoia
insomnia	photophobia
insomnia	ventricular tachycardia
insomnia	somnolence
insomnia	palpitations
insomnia	neurosis
insomnia	sweating
insomnia	torticollis
insomnia	tachycardia
insomnia	keratitis
insomnia	menorrhagia

insomnia	personality disorder
insomnia	psychosis
insomnia	serotonin syndrome
insomnia	supraventricular extrasystoles
insomnia	weight gain
insomnia	priapism
insomnia	urinary incontinence
insomnia	postural hypotension
interstitial nephritis	rectal hemorrhage
interstitial nephritis	melena
interstitial nephritis	toxic epidermal necrolysis
interstitial nephritis	proteinuria
interstitial nephritis	peptic ulcer
interstitial nephritis	sepsis
interstitial nephritis	ulcer
interstitial nephritis	vasculitis
interstitial nephritis	pancreatitis
interstitial nephritis	pneumonia
interstitial nephritis	renal failure
interstitial nephritis	lymphadenopathy
interstitial nephritis	stomatitis
interstitial nephritis	pancytopenia
interstitial nephritis	tinnitus
interstitial nephritis	jaundice
interstitial nephritis	photosensitivity
interstitial nephritis	ulcerative stomatitis
interstitial nephritis	thrombocytopenia
ischemic colitis	leukocytosis
ischemic colitis	neuralgia
ischemic colitis	skin ulcer
ischemic colitis	suicide attempt
jaundice	toxic epidermal necrolysis
jaundice	photosensitivity
jaundice	rectal hemorrhage
jaundice	pancytopenia
jaundice	purpura
jaundice	pancreatitis
jaundice	peptic ulcer
jaundice	pruritus
keratitis	serotonin syndrome
keratitis	supraventricular extrasystoles
keratitis	paranoia
keratitis	photophobia
keratitis	ventricular tachycardia
keratitis	nervousness
keratitis	psoriasis
keratitis	sweating
keratitis	torticollis
keratitis	nightmares
keratitis	priapism
keratitis	syncope
keratitis	somnolence
keratitis	tardive dyskinesia
keratitis	urinary retention

keratitis	palpitations
keratitis	paralytic ileus
keratitis	nasal congestion
labyrinthitis	pyelonephritis
labyrinthitis	sneezing
labyrinthitis	stridor
labyrinthitis	urinary hesitancy
labyrinthitis	ventricular extrasystoles
labyrinthitis	voice alteration
labyrinthitis	wheezing
lactic dehydrogenase increased	pyuria
laryngitis	urinary incontinence
laryngitis	urinary frequency
laryngitis	tachycardia
laryngitis	tremor
laryngitis	otitis media
laryngitis	phlebitis
laryngitis	pulmonary embolism
laryngitis	sleep disorder
laryngitis	liver function tests abnormal
laryngitis	migraine
laryngitis	thrombophlebitis
laryngitis	somnolence
laryngitis	ventricular fibrillation
leukocytosis	neuralgia
leukocytosis	skin ulcer
leukocytosis	suicide attempt
leukopenia	stomatitis
leukopenia	neutropenia
leukopenia	peripheral neuropathy
leukopenia	thrombocytopenia
leukopenia	neuropathy
leukopenia	toxic epidermal necrolysis
leukopenia	sepsis
leukopenia	pleural effusion
leukopenia	pneumonia
leukopenia	renal failure
lightheadedness	sore throat
lightheadedness	mental depression
lightheadedness	nightmares
lightheadedness	memory loss
lightheadedness	psoriasis
lightheadedness	spasm
lightheadedness	sweating
lightheadedness	weakness
lightheadedness	priapism
liver function tests abnormal	migraine
liver function tests abnormal	otitis media
liver function tests abnormal	phlebitis
liver function tests abnormal	pulmonary embolism
liver function tests abnormal	lymphadenopathy
liver function tests abnormal	neurosis
liver function tests abnormal	shock
liver function tests abnormal	urinary incontinence

liver function tests abnormal	urinary frequency
lupus	psychosis
lymphadenopathy	tinnitus
lymphadenopathy	rectal hemorrhage
lymphadenopathy	migraine
lymphadenopathy	otitis media
lymphadenopathy	phlebitis
lymphadenopathy	pulmonary embolism
lymphadenopathy	tremor
lymphadenopathy	toxic epidermal necrolysis
lymphadenopathy	myocardial infarction
lymphadenopathy	neurosis
lymphadenopathy	peptic ulcer
lymphadenopathy	stomatitis
malaise	toxic epidermal necrolysis
malaise	pancreatitis
malaise	myalgia
melena	rectal hemorrhage
melena	proteinuria
melena	peptic ulcer
melena	sepsis
melena	toxic epidermal necrolysis
melena	ulcer
melena	vasculitis
melena	pneumonia
melena	renal failure
melena	pancytopenia
melena	pancreatitis
melena	stomatitis
melena	thrombocytopenia
memory loss	sore throat
memory loss	mental depression
memory loss	nightmares
menorrhagia	neurosis
menorrhagia	nightmares
menorrhagia	syncope
menorrhagia	urinary incontinence
menorrhagia	urinary retention
mental depression	sore throat
mental depression	nightmares
mental depression	shortness of breath
mental depression	nasal congestion
mental depression	postural hypotension
mental depression	psoriasis
mental depression	spasm
mental depression	sweating
mental depression	weakness
migraine	otitis media
migraine	phlebitis
migraine	pulmonary embolism
migraine	neurosis
migraine	shock
migraine	urinary incontinence
migraine	urinary frequency

mouth ulceration	petechiae
mouth ulceration	swelling
muscle cramps	rhinitis
muscle cramps	upper respiratory tract infection
myalgia	rash
myalgia	thrombocytopenia
myocardial infarction	thrombophlebitis
myocardial infarction	pulmonary embolism
nasal congestion	postural hypotension
nasal congestion	tardive dyskinesia
nasal congestion	paralytic ileus
nasal congestion	priapism
nasal congestion	spasm
nasal congestion	sweating
nasal congestion	palpitations
nasal congestion	torticollis
nasal congestion	nightmares
nasal congestion	paranoia
nasal congestion	ventricular tachycardia
nasal congestion	tachycardia
nasal congestion	urinary retention
nasal congestion	psoriasis
nasal congestion	personality disorder
nasal congestion	serotonin syndrome
nasal congestion	shortness of breath
nasal congestion	supraventricular extrasystoles
nasal congestion	weight gain
neck rigidity	polyuria
neck rigidity	tenesmus
neck rigidity	vaginal hemorrhage
neck rigidity	vaginitis
nervousness	syncope
nervousness	urinary retention
nervousness	photophobia
nervousness	tremor
nervousness	nightmares
nervousness	somnolence
nervousness	serotonin syndrome
nervousness	supraventricular extrasystoles
nervousness	paralysis
nervousness	paranoia
nervousness	ventricular tachycardia
nervousness	tachycardia
nervousness	postural hypotension
nervousness	neurosis
nervousness	psoriasis
nervousness	sweating
neuralgia	skin ulcer
neuralgia	suicide attempt
neuropathy	stomatitis
neuropathy	neutropenia
neuropathy	pleural effusion
neuropathy	peripheral neuropathy
neuropathy	toxic epidermal necrolysis

neuropathy	sepsis
neuropathy	thrombocytopenia
neurosis	tachycardia
neurosis	tremor
neurosis	nightmares
neurosis	syncope
neurosis	urinary incontinence
neurosis	somnolence
neurosis	urinary frequency
neurosis	urinary retention
neurosis	weight gain
neurosis	otitis media
neurosis	paranoia
neurosis	phlebitis
neurosis	pulmonary embolism
neurosis	ventricular fibrillation
neurosis	ventricular tachycardia
neurosis	psoriasis
neurosis	sweating
neutropenia	stomatitis
neutropenia	thrombocytopenia
neutropenia	pleural effusion
neutropenia	peripheral neuropathy
nightmares	urinary retention
nightmares	psoriasis
nightmares	sweating
nightmares	tachycardia
nightmares	priapism
nightmares	syncope
nightmares	paranoia
nightmares	sore throat
nightmares	ventricular tachycardia
nightmares	tardive dyskinesia
nightmares	palpitations
nightmares	paralytic ileus
nightmares	spasm
nightmares	torticollis
nightmares	postural hypotension
nightmares	tremor
nightmares	urinary incontinence
nightmares	personality disorder
nightmares	serotonin syndrome
nightmares	supraventricular extrasystoles
nightmares	weight gain
nightmares	somnolence
nightmares	photophobia
otitis media	phlebitis
otitis media	urinary incontinence
otitis media	urinary frequency
otitis media	tachycardia
otitis media	tremor
otitis media	pulmonary embolism
otitis media	sleep disorder
otitis media	ventricular fibrillation

otitis media	tinnitus
otitis media	shock
palpitations	priapism
palpitations	tardive dyskinesia
palpitations	spasm
palpitations	sweating
palpitations	postural hypotension
palpitations	paralytic ileus
palpitations	torticollis
palpitations	syncope
palpitations	tachycardia
palpitations	paranoia
palpitations	ventricular tachycardia
palpitations	urinary retention
palpitations	psoriasis
palpitations	weakness
palpitations	personality disorder
palpitations	serotonin syndrome
palpitations	supraventricular extrasystoles
palpitations	weight gain
palpitations	tremor
pancreatitis	toxic epidermal necrolysis
pancreatitis	stomatitis
pancreatitis	pneumonia
pancreatitis	rectal hemorrhage
pancreatitis	purpura
pancreatitis	proteinuria
pancreatitis	vasculitis
pancreatitis	pancytopenia
pancreatitis	sepsis
pancreatitis	upper respiratory tract infection
pancreatitis	peptic ulcer
pancreatitis	thrombocytopenia
pancreatitis	photosensitivity
pancytopenia	sepsis
pancytopenia	peptic ulcer
pancytopenia	pneumonia
pancytopenia	rectal hemorrhage
pancytopenia	proteinuria
pancytopenia	ulcer
pancytopenia	thrombocytopenia
pancytopenia	pruritus
pancytopenia	toxic epidermal necrolysis
pancytopenia	stomatitis
paralysis	shock
paralysis	syncope
paralytic ileus	tardive dyskinesia
paralytic ileus	priapism
paralytic ileus	torticollis
paralytic ileus	tachycardia
paralytic ileus	urinary retention
paralytic ileus	spasm
paralytic ileus	sweating
paralytic ileus	postural hypotension

paralytic ileus	paranoia
paralytic ileus	ventricular tachycardia
paralytic ileus	psoriasis
paralytic ileus	personality disorder
paralytic ileus	serotonin syndrome
paralytic ileus	supraventricular extrasystoles
paralytic ileus	urinary urgency
paralytic ileus	weight gain
paralytic ileus	syncope
paranoia	ventricular tachycardia
paranoia	psoriasis
paranoia	sweating
paranoia	torticollis
paranoia	priapism
paranoia	syncope
paranoia	somnolence
paranoia	tardive dyskinesia
paranoia	urinary retention
paranoia	personality disorder
paranoia	serotonin syndrome
paranoia	supraventricular extrasystoles
paranoia	weight gain
paranoia	tachycardia
paranoia	postural hypotension
paranoia	tremor
paranoia	photophobia
paranoia	seizures
paranoia	spasm
paranoia	urinary incontinence
paresthesia	tinnitus
paresthesia	photosensitivity
paresthesia	syncope
paresthesia	urinary frequency
Parkinson	increased salivation
Parkinson	tardive dyskinesia
Parkinson	paralytic ileus
Parkinson	galactorrhea
Parkinson	priapism
Parkinson	tachycardia
Parkinson	urinary retention
Parkinson	dyskinesia
Parkinson	torticollis
Parkinson	dry mouth
Parkinson	nightmares
Parkinson	impotence
Parkinson	nasal congestion
Parkinson	spasm
Parkinson	sweating
Parkinson	palpitations
Parkinson	gynecomastia
Parkinson	heart block
Parkinson	glycosuria
Parkinson	syncope
Parkinson	amenorrhea

Parkinson	grand mal
Parkinson	paranoia
Parkinson	ventricular tachycardia
Parkinson	postural hypotension
Parkinson	bradycardia
Parkinson	constipation
Parkinson	hypotension
Parkinson	insomnia
Parkinson	nervousness
Parkinson	neurosis
Parkinson	psoriasis
Parkinson	delirium
Parkinson	abnormal gait
Parkinson	abnormal vision
Parkinson	alkaline phosphatase increased
Parkinson	blepharitis
Parkinson	blurred vision
Parkinson	cold extremities
Parkinson	dysmenorrhea
Parkinson	dysphagia
Parkinson	goiter
Parkinson	hypothyroidism
Parkinson	keratitis
Parkinson	menorrhagia
Parkinson	personality disorder
Parkinson	serotonin syndrome
Parkinson	supraventricular extrasystoles
Parkinson	urinary urgency
Parkinson	weight gain
Parkinson	hallucinations
Parkinson	urinary incontinence
Parkinson	tremor
peptic ulcer	rectal hemorrhage
peptic ulcer	proteinuria
peptic ulcer	sepsis
peptic ulcer	toxic epidermal necrolysis
peptic ulcer	ulcer
peptic ulcer	vasculitis
peptic ulcer	pneumonia
peptic ulcer	renal failure
peptic ulcer	pruritus
peptic ulcer	stomatitis
peripheral edema	weight loss
peripheral neuropathy	stomatitis
peripheral neuropathy	toxic epidermal necrolysis
peripheral neuropathy	sepsis
peripheral neuropathy	thrombocytopenia
peripheral neuropathy	pleural effusion
peripheral neuropathy	pneumonia
personality disorder	ventricular tachycardia
personality disorder	psoriasis
personality disorder	seizures
personality disorder	spasm
personality disorder	sweating

personality disorder	torticollis
personality disorder	priapism
personality disorder	syncope
personality disorder	urinary incontinence
personality disorder	somnolence
personality disorder	tardive dyskinesia
personality disorder	urinary retention
petechiae	swelling
Pharyngitis	upper respiratory tract infection
Pharyngitis	Sinusitis
Pharyngitis	fever
Pharyngitis	abdominal pain
Pharyngitis	bronchitis
phlebitis	urinary incontinence
phlebitis	urinary frequency
phlebitis	tachycardia
phlebitis	tremor
phlebitis	pulmonary embolism
phlebitis	sleep disorder
phlebitis	ventricular fibrillation
phlebitis	tinnitus
phlebitis	shock
photophobia	syncope
photophobia	somnolence
photophobia	urinary retention
photophobia	serotonin syndrome
photophobia	supraventricular extrasystoles
photophobia	postural hypotension
photophobia	tremor
photophobia	ventricular tachycardia
photophobia	psoriasis
photophobia	sweating
photophobia	torticollis
photophobia	priapism
photosensitivity	purpura
photosensitivity	tinnitus
photosensitivity	vertigo
photosensitivity	rectal hemorrhage
photosensitivity	toxic epidermal necrolysis
photosensitivity	vasculitis
photosensitivity	rhinitis
photosensitivity	renal failure
photosensitivity	thrombocytopenia
pleural effusion	toxic epidermal necrolysis
pleural effusion	stomatitis
pleural effusion	sepsis
pleural effusion	pulmonary edema
pneumonia	toxic epidermal necrolysis
pneumonia	sepsis
pneumonia	proteinuria
pneumonia	ulcer
pneumonia	thrombocytopenia
pneumonia	rectal hemorrhage
pneumonia	vasculitis

pneumonia	pruritus
pneumonia	renal failure
pneumonia	upper respiratory tract infection
pneumonia	stomatitis
polyuria	tenesmus
polyuria	vaginal hemorrhage
polyuria	vaginitis
postural hypotension	priapism
postural hypotension	syncope
postural hypotension	spasm
postural hypotension	sweating
postural hypotension	tardive dyskinesia
postural hypotension	urinary retention
postural hypotension	torticollis
postural hypotension	ventricular tachycardia
postural hypotension	tachycardia
postural hypotension	somnolence
postural hypotension	psoriasis
postural hypotension	weakness
priapism	tardive dyskinesia
priapism	torticollis
priapism	tachycardia
priapism	urinary retention
priapism	spasm
priapism	sweating
priapism	ventricular tachycardia
priapism	psoriasis
priapism	syncope
priapism	serotonin syndrome
priapism	supraventricular extrasystoles
priapism	urinary urgency
priapism	weight gain
priapism	somnolence
priapism	sore throat
proteinuria	rectal hemorrhage
proteinuria	toxic epidermal necrolysis
proteinuria	sepsis
proteinuria	ulcer
proteinuria	vasculitis
proteinuria	renal failure
proteinuria	pulmonary edema
pruritus	thrombocytopenia
pruritus	ulcer
pruritus	vasculitis
pruritus	renal failure
pruritus	sepsis
pruritus	toxic epidermal necrolysis
psoriasis	ventricular tachycardia
psoriasis	sweating
psoriasis	torticollis
psoriasis	syncope
psoriasis	somnolence
psoriasis	tardive dyskinesia
psoriasis	urinary retention

psoriasis	serotonin syndrome
psoriasis	supraventricular extrasystoles
psoriasis	weight gain
psoriasis	sore throat
psoriasis	tachycardia
psoriasis	tremor
psoriasis	seizures
psoriasis	spasm
psychosis	syncope
psychosis	somnolence
psychosis	urinary retention
pulmonary edema	toxic epidermal necrolysis
pulmonary edema	renal failure
pulmonary embolism	urinary frequency
pulmonary embolism	thrombophlebitis
pulmonary embolism	shock
pulmonary embolism	urinary incontinence
purpura	vertigo
pyelonephritis	sneezing
pyelonephritis	stridor
pyelonephritis	urinary hesitancy
pyelonephritis	ventricular extrasystoles
pyelonephritis	voice alteration
pyelonephritis	wheezing
rectal hemorrhage	toxic epidermal necrolysis
rectal hemorrhage	sepsis
rectal hemorrhage	ulcer
rectal hemorrhage	vasculitis
rectal hemorrhage	renal failure
rectal hemorrhage	stomatitis
rectal hemorrhage	tinnitus
rectal hemorrhage	ulcerative stomatitis
rectal hemorrhage	thrombocytopenia
renal failure	thrombocytopenia
renal failure	vasculitis
renal failure	toxic epidermal necrolysis
renal failure	sepsis
renal failure	ulcer
renal failure	stomatitis
renal failure	tinnitus
renal failure	vertigo
seizures	ventricular tachycardia
seizures	spasm
seizures	sweating
sepsis	toxic epidermal necrolysis
sepsis	stomatitis
sepsis	ulcer
sepsis	thrombocytopenia
sepsis	vasculitis
serotonin syndrome	supraventricular extrasystoles
serotonin syndrome	ventricular tachycardia
serotonin syndrome	sweating
serotonin syndrome	torticollis
serotonin syndrome	syncope

serotonin syndrome	somnolence
serotonin syndrome	tardive dyskinesia
serotonin syndrome	urinary retention
SGOT increased	interstitial pneumonitis
SIADH	hallucinations
SIADH	urinary incontinence
SIADH	confusion
SIADH	ataxia
SIADH	tachycardia
SIADH	breast pain
SIADH	hypoxia
SIADH	tremor
SIADH	laryngitis
SIADH	coma
SIADH	urinary frequency
SIADH	otitis media
SIADH	phlebitis
SIADH	sleep disorder
SIADH	ventricular fibrillation
SIADH	eructation
SIADH	hypotension
SIADH	amnesia
SIADH	hiccup
SIADH	neurosis
SIADH	albuminuria
SIADH	delirium
SIADH	glossitis
SIADH	diplopia
SIADH	herpes simplex
SIADH	somnolence
SIADH	hypothyroidism
SIADH	liver function tests abnormal
SIADH	menorrhagia
SIADH	migraine
SIADH	breast enlargement
SIADH	pulmonary embolism
Sinusitis	upper respiratory tract infection
Sinusitis	conjunctivitis
Sinusitis	bronchitis
Sinusitis	abdominal pain
Sinusitis	fever
skin ulcer	suicide attempt
sleep disorder	urinary incontinence
sleep disorder	urinary frequency
sleep disorder	tachycardia
sleep disorder	tremor
sleep disorder	ventricular fibrillation
sneezing	stridor
sneezing	urinary hesitancy
sneezing	ventricular extrasystoles
sneezing	voice alteration
sneezing	wheezing
somnolence	tremor
somnolence	tachycardia

somnolence	syncope
somnolence	urinary incontinence
somnolence	ventricular tachycardia
somnolence	urinary retention
somnolence	sweating
somnolence	torticollis
somnolence	supraventricular extrasystoles
somnolence	weight gain
somnolence	tardive dyskinesia
somnolence	urinary frequency
sore throat	spasm
sore throat	sweating
sore throat	weakness
spasm	tardive dyskinesia
spasm	tachycardia
spasm	sweating
spasm	torticollis
spasm	urinary retention
spasm	ventricular tachycardia
spasm	weakness
Stevens - Johnson syndrome	erythema multiforme
Stevens - Johnson syndrome	toxic epidermal necrolysis
Stevens - Johnson syndrome	hemolytic anemia
Stevens - Johnson syndrome	hematemesis
Stevens - Johnson syndrome	interstitial nephritis
Stevens - Johnson syndrome	rectal hemorrhage
Stevens - Johnson syndrome	renal failure
Stevens - Johnson syndrome	exfoliative dermatitis
Stevens - Johnson syndrome	vasculitis
Stevens - Johnson syndrome	gastrointestinal hemorrhage
Stevens - Johnson syndrome	aplastic anemia
Stevens - Johnson syndrome	hearing loss
Stevens - Johnson syndrome	melena
Stevens - Johnson syndrome	gastritis
Stevens - Johnson syndrome	colitis
Stevens - Johnson syndrome	ecchymosis
Stevens - Johnson syndrome	cystitis
Stevens - Johnson syndrome	peptic ulcer
Stevens - Johnson syndrome	sepsis
Stevens - Johnson syndrome	hepatic failure
Stevens - Johnson syndrome	proteinuria
Stevens - Johnson syndrome	pancreatitis
Stevens - Johnson syndrome	alopecia
Stevens - Johnson syndrome	eructation
Stevens - Johnson syndrome	tinnitus
Stevens - Johnson syndrome	congestive heart failure
Stevens - Johnson syndrome	thrombocytopenia
Stevens - Johnson syndrome	stomatitis
Stevens - Johnson syndrome	angioedema
Stevens - Johnson syndrome	photosensitivity
Stevens - Johnson syndrome	pulmonary edema
Stevens - Johnson syndrome	asthenia
Stevens - Johnson syndrome	hematuria
Stevens - Johnson syndrome	dysuria

Stevens - Johnson syndrome	flatulence
Stevens - Johnson syndrome	herpes simplex
Stevens - Johnson syndrome	ulcer
Stevens - Johnson syndrome	peripheral neuropathy
Stevens - Johnson syndrome	agranulocytosis
Stevens - Johnson syndrome	dry skin
Stevens - Johnson syndrome	esophagitis
Stevens - Johnson syndrome	lymphadenopathy
Stevens - Johnson syndrome	hepatitis
Stevens - Johnson syndrome	jaundice
Stevens - Johnson syndrome	constipation
Stevens - Johnson syndrome	leukopenia
stomatitis	toxic epidermal necrolysis
stomatitis	thrombocytopenia
stomatitis	weight loss
stridor	urinary hesitancy
stridor	ventricular extrasystoles
stridor	voice alteration
stridor	wheezing
supraventricular extrasystoles	ventricular tachycardia
supraventricular extrasystoles	sweating
supraventricular extrasystoles	torticollis
supraventricular extrasystoles	syncope
supraventricular extrasystoles	tardive dyskinesia
supraventricular extrasystoles	urinary retention
sweating	tardive dyskinesia
sweating	urinary retention
sweating	ventricular tachycardia
sweating	tachycardia
sweating	torticollis
sweating	syncope
sweating	weight gain
sweating	tremor
sweating	weakness
syncope	urinary retention
syncope	tremor
syncope	ventricular tachycardia
syncope	tachycardia
syncope	torticollis
syncope	urinary incontinence
syncope	weight gain
syncope	tardive dyskinesia
syncope	urinary frequency
tachycardia	urinary incontinence
tachycardia	tardive dyskinesia
tachycardia	urinary retention
tachycardia	torticollis
tachycardia	tremor
tachycardia	urinary frequency
tachycardia	ventricular fibrillation
tachycardia	ventricular tachycardia
tardive dyskinesia	torticollis
tardive dyskinesia	urinary retention
tardive dyskinesia	ventricular tachycardia

tardive dyskinesia	urinary urgency
tardive dyskinesia	weight gain
tenesmus	vaginal hemorrhage
tenesmus	vaginitis
thrombocytopenia	toxic epidermal necrolysis
thrombocytopenia	vasculitis
thrombocytopenia	ulcer
thrombophlebitis	urinary frequency
tinnitus	urinary frequency
tinnitus	vasculitis
Toothache	abuse
Toothache	drug dependence
Toothache	fecal impaction
Toothache	glaucoma
Toothache	hyperacusis
Toothache	hypothermia
Toothache	hypoventilation
Toothache	labyrinthitis
Toothache	pyelonephritis
Toothache	sneezing
Toothache	stridor
Toothache	urinary hesitancy
Toothache	ventricular extrasystoles
Toothache	voice alteration
Toothache	wheezing
torticollis	urinary retention
torticollis	ventricular tachycardia
torticollis	urinary urgency
torticollis	weight gain
toxic epidermal necrolysis	ulcer
toxic epidermal necrolysis	vasculitis
tremor	urinary incontinence
tremor	urinary frequency
tremor	urinary retention
tremor	ventricular fibrillation
tremor	ventricular tachycardia
ulcer	vasculitis
urinary frequency	urinary incontinence
urinary hesitancy	ventricular extrasystoles
urinary hesitancy	voice alteration
urinary hesitancy	wheezing
urinary incontinence	ventricular fibrillation
urinary incontinence	urinary retention
urinary incontinence	ventricular tachycardia
urinary retention	ventricular tachycardia
urinary retention	urinary urgency
urinary retention	weight gain
vaginal hemorrhage	vaginitis
Vascular Disorders	constitutional symptoms
Vascular Disorders	hemorrhage
Vascular Disorders	flushing
Vascular Disorders	peripheral edema
vasculitis	vertigo
ventricular extrasystoles	voice alteration

ventricular extrasystoles	wheezing
ventricular tachycardia	weight gain
voice alteration	wheezing

Table S1. All cliques in the ADR-ADR network

Clique ID	ADR	ADR	ADR	ADR	ADR	ADR
1	alopecia	hypersensitivity	infection	fever	stomatitis	anorexia
2	alopecia	infection	pancreatitis	fever	stomatitis	anorexia
3	delirium	hallucinations	tachycardia	urinary incontinence	SIADH	breast enlargement
4	galactorrhea	delirium	hallucinations	tachycardia	urinary incontinence	breast enlargement
5	cold extremities	fatigue	nightmares	syncope	weight gain	constipation
6	abdominal pain	alopecia	hypersensitivity	infection	anorexia	dermatitis
7	abdominal pain	alopecia	infection	pancreatitis	anorexia	dermatitis
8	abdominal pain	alopecia	hypersensitivity	infection	myalgia	dermatitis
9	infection	congestive heart failure	hematuria	anorexia	dyspnea	edema
10	exfoliative dermatitis	esophagitis	jaundice	peptic ulcer	pancytopenia	eosinophilia
11	esophagitis	jaundice	peptic ulcer	pruritus	pancytopenia	eosinophilia
12	congestive heart failure	stomatitis	constitutional symptoms	hemorrhage	anemia	erythema
13	abdominal pain	conjunctivitis	epistaxis	pancreatitis	upper respiratory tract infection	fever
14	alopecia	angioedema	hepatitis	vasculitis	vertigo	hematuria
15	angioedema	hepatitis	vasculitis	vertigo	hematuria	hemolytic anemia
16	angioedema	hepatitis	photosensitivity	vasculitis	vertigo	hemolytic anemia
17	angioedema	exfoliative dermatitis	flatulence	photosensitivity	purpura	jaundice
18	angioedema	exfoliative dermatitis	pancreatitis	photosensitivity	purpura	jaundice
19	abdominal pain	conjunctivitis	epistaxis	hepatitis	upper respiratory tract infection	muscle cramps
20	Dyspepsia	gastroenteritis	hepatitis	hyperkalemia	pneumonia	proteinuria
21	Dyspepsia	angioedema	exfoliative dermatitis	flatulence	photosensitivity	purpura
22	Dyspepsia	angioedema	exfoliative dermatitis	pancreatitis	photosensitivity	purpura
23	impotence	palpitations	postural hypotension	sweating	weakness	spasm
24	lightheadedness	mental depression	sore throat	sweating	weakness	spasm
25	Dyspepsia	alopecia	anaphylaxis	malaise	pancreatitis	toxic epidermal necrolysis
26	Dyspepsia	gastroenteritis	pneumonia	proteinuria	toxic epidermal necrolysis	ulcer
27	Dyspepsia	Influenza	abdominal pain	conjunctivitis	epistaxis	upper respiratory tract infection
28	Dyspepsia	hepatitis	hyperkalemia	pneumonia	proteinuria	vasculitis
29	AV block	impotence	palpitations	postural hypotension	sweating	weakness
30	AV block	lightheadedness	mental depression	sore throat	sweating	weakness
